# Divergence from the classical hydroboration reactivity; boron containing materials through a hydroboration cascade of small cyclic dienes†
†Electronic supplementary information (ESI) available. See DOI: 10.1039/c4sc02729a


**DOI:** 10.1039/c4sc02729a

**Published:** 2015-08-06

**Authors:** Anna Andreou, Michal Leskes, Pablo G. Jambrina, Gary J. Tustin, Clare P. Grey, Edina Rosta, Oren A. Scherman

**Affiliations:** a Melville Laboratory for Polymer Synthesis , Department of Chemistry , University of Cambridge , Lensfield Road , Cambridge CB2 1EW , UK . Email: oas23@cam.ac.uk; b Department of Chemistry , University of Cambridge , Lensfield Road , Cambridge CB2 1EW , UK; c Department of Chemistry , Kings College London , Strand , London , WC2R 2LS , UK; d Schlumberger Cambridge Research , High Cross, Madingley Road , Cambridge , CB3 0EL , UK

## Abstract

The hydroboration 1,3- and 1,4-cyclic dienes has been systematically investigated. The behavior of such dienes towards mono and dihydroboration was monitored directly by ^11^B NMR to identify the actual boron species formed, as opposed to the most common analysis of the resultant oxidation products. Quantitative dihydroboration was achieved for the full range of cyclic dienes investigated including dienes, which were previously reported to be resistant to dihydroboration, leading to the formation of new boron-containing polymeric materials. The conditions favoring dihydroboration are reported as well as full characterisation of the materials. Furthermore, a hydroboration cascade mechanism is proposed for the formation of such boron-containing polymers, supported by both experimental and theoretical data.

## Abstract 2

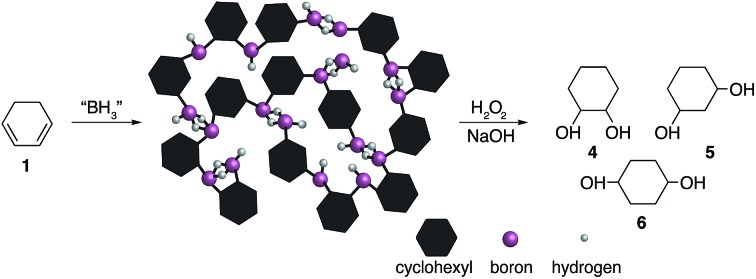
Quantitative dihydroboration of cyclic dienes leading to new, tunable boron-containing hydrocarbon polymeric materials.

## 1 Introduction

Hydroboration is undoubtedly a popular and widely used synthetic reaction.^1^,^2^ More specifically, hydroboration and subsequent oxidation of dienes to their corresponding diols has received much attention mainly on account of their wide usage in applications such as adhesives,^3^,^4^ polymeric materials,^5^,^6^ pharmaceuticals,^7^,^8^ boronic acid–diol sensors^9^,^10^ and as intermediates for further synthetic transformations such as in the synthesis of chiral ligands BINAP,^11^,^12^ DuPHOS^13^,^14^ amongst others. Despite the fact that dihydroboration of dienes followed by basic oxidation appears an attractive synthetic route to obtain diols, such compounds are typically prepared using epoxide hydrolysis,^15^,^16^ oxidation of alkenes,^17^,^18^ the Prévost reaction (*anti* diols)^19^–^21^ and the Woodward *cis*-hydroxylation (*cis* diols)^22^ giving rise to high stereo and regio control.

**Fig. 1:**
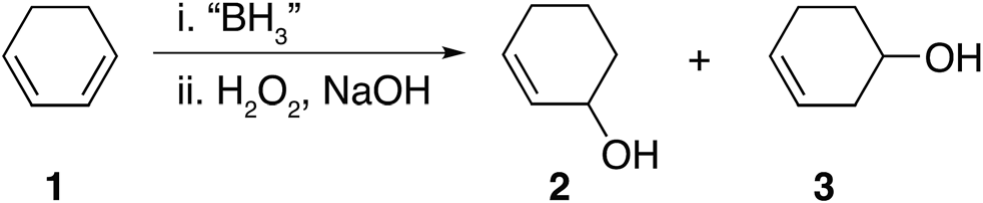
Hydroboration–oxidation of 1,3-cyclohexadiene **1** as reported by Brown *et al.*^24^,^25^

It is possible that dihydroboration of cyclic dienes to generate diols has not been thoroughly explored as a result of the strong and contradicting conclusions drawn in a series of published studies by H. C. Brown *et al.*^23^–^25^ In these publications, the hydroboration of a series of cyclic dienes was explored. Brown *et al.* concluded that smaller cyclic rings, such as 1,3-cyclohexadiene **1**, undergo dihydroboration only to a very small extent and were therefore concluded to be resistant based on the observation of selective formation of the monohydroboration products.^23^,^25^ In the case of **1**, basic oxidation and gas chromatography (GC) analysis of the hydroboration mixture was reported to yield the formation of 2-cylohexene-1-ol **2** and 3-cyclohexene-1-ol **3** as shown in Fig. 1.^25^ Contradictingly, in a singular older report, H. C. Brown *et al.* mentioned the possibility of polymer formation *via* dihyrdroboration of cyclic dienes but have never investigated the outcome of such reactions.^24^ Research conducted by contemporaries in the field reported improved yields, however, generally following the same trend that smaller rings appeared to be resistant to dihydroboration.^26^ For example, when K. J. Saegebarth used a large excess (80%) of diborane(6) with 1,3-cyclopentadiene, an increased yield of *cis*-1,3-cyclopentanediol was obtained after oxidation, with monohydroboration, however, still being the main reaction. We found the exceptional resistance of smaller rings towards dihydroboration, even in the cases where excess hydroborating agent was used, rather puzzling. Although a diene is certainly a more electron rich system when compared to an alkene, the non-conjugated nature of the C=C bond present in the formed monohydroboration products could potentially compel them to be more reactive than the starting diene, on account of the non-conjugated double bond compared to the initial system. Additionally, both the cyclic diene and the borane reagents (both the starting borane and the formed products) are capable of undergoing further hydroboration reactions. Thus, one might expect the formation of polymeric and/or network materials, as was previously reported in the case of straight-chain dienes^27^–^30^ or the formation of oligomers through ring opening polymerization.^31^

Unfortunately, almost all of these studies relied upon indirect observations of the subsequently isolated hydroxylated species formed after oxidation, rather than gaining direct evidence arising from the borane species. To date there have only been a few published hydroboration studies that analyze the reaction outcome directly, *i.e.* by identifying the boron species present.^32^,^33^ We describe herein a systematic investigation of the hydroboration of 1,3-cyclohexadiene **1** (for other cyclic dienes see ESI S10†) and its behavior towards mono and dihydroboration by direct observation of the boron species formed. The reaction conditions favoring dihydroboration lead to the formation of new boron-containing polymeric materials, and a mechanism for the formation of such materials is proposed and supported by both experimental and theoretical data.

## 2 Results and discussion

As a result of our interest in preparing substituted diboranes, we aimed to systematically investigate the hydroboration of cyclic dienes in order to identify the suitable conditions for dihydroboration of smaller rings. While the results and conclusions by Brown *et al.* were certainly a concern, we were encouraged by the singular literature report by Saegebarth, who reported up to 40% dihydroboration of 1,3-cyclopentadiene when a large excess of diborane(6) was used.^26^

**Fig. 2:**
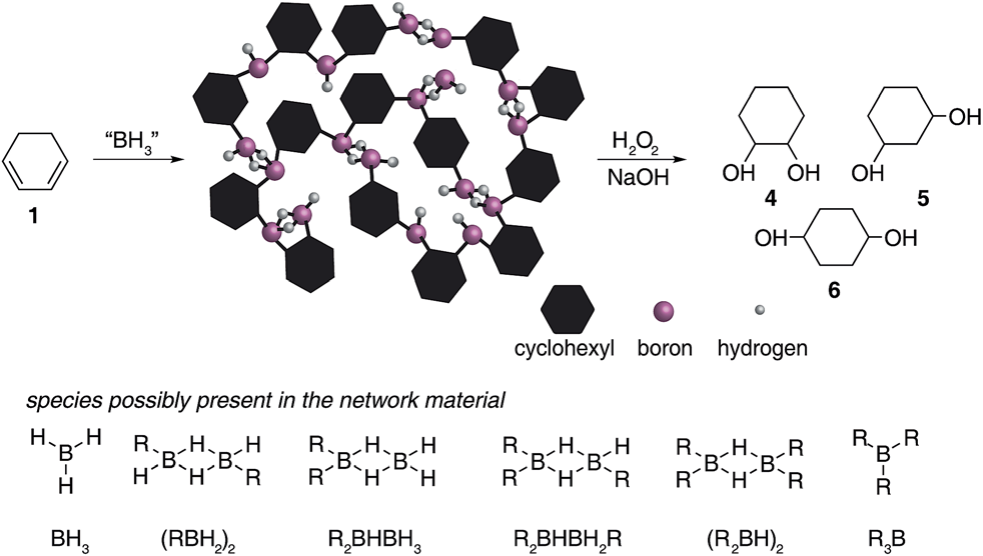
Hydroboration of 1,3-cyclohexadiene **1** leading to the formation of insoluble materials which contain a range of borane species.

We first focused our attention on the number of equivalents of diene present in the reaction, as the large excess of **1** utilized by H. C. Brown *et al.*^24^,^25^ seemed unnecessary and was possibly the determining factor for the selective monohydroboration observed in their system. The number of equivalents of diene used was kept constant (1 equivalent) while the amount of borane was explored. Additionally, we investigated two modes of reagent addition: borane to diene and diene to borane. As can be clearly seen in Table 1, irrespective of borane molar equivalents and the mode of reagent addition, all entries yielded a white, insoluble precipitate. When THF was used as the solvent, a glassy material was formed as shown in Fig. 2, which gave rise to complications in characterisation of the products; therefore, in an attempt to eliminate the possibility of side reactions such as THF ring opening (known to be initiated by borane),^34^ diglyme was chosen as the reaction solvent (see ESI S6†). Moreover, the use of diglyme allowed for direct comparison of the reactions performed when two different borane sources were explored (BH_3_·SMe_2_*vs.* B_2_H_6_ prepared from NaBH_4_ and BF_3_·OEt_2_).

**Table 1:** Hydroboration of 1,3-cyclohexadiene **1** in diglyme using different equivalents of borane originating from different sources

Entry	Borane (molar equiv.)	Mode of reagent addition	Observation
1	0.33	Borane : diene	White precipitate formed^*a*^
		Diene : borane	Unreacted C=C observed^*a*^
2	0.50	Borane : diene	White precipitate formed^*a*^
		Diene : borane	No C=C observed^*a*^
			Unreacted borane^*a*^
3	1.0	Borane : diene	White precipitate formed^*a*^
		Diene : borane	No C=C observed^*a*^
			Unreacted borane^*a*^
4	2.0	Borane : diene	White precipitate formed^*a*^
		Diene : borane	No C=C observed^*a*^
			Unreacted borane^*a*^
5	0.25	BF_3_·OEt_2_ to NaBH_4_	White precipitate formed^*b*^
		NaBH_4_ to BF_3_OEt_2_	Unreacted C=C observed^*c*^
			No C=C observed^*d*^
6	0.5	BF_3_·OEt_2_ to NaBH_4_	White precipitate formed^*b*^
		NaBH_4_ to BF_3_OEt_2_	No C=C observed^*c*^
			Unreacted C=C observed^*d*^
7	1.0	BF_3_·OEt_2_ to NaBH_4_	White precipitate formed^*b*^
		NaBH_4_ to BF_3_·OEt_2_	No C=C observed^*b*^

^*a*^The mode of reagent addition (borane : diene or diene : borane) yielded the same results.

^*b*^Observed during both modes of addition.

^*c*^Observed during BF_3_·OEt_2_ to NaBH_4_ addition.

^*d*^Observed during NaBH_4_ to BF_3_·OEt_2_.

In order to fully characterise the reaction, the precipitates were collected by filtration (under nitrogen) and the filtrates were analysed by NMR. All B–H bonds appeared to undergo hydroboration, as unsaturated C=C bonds were only observed in entry 1, while the borane starting reagent was fully consumed. Analysis of the filtrates obtained for entries 2–4 (molar borane equivalents of 0.5, 1.0 and 2.0, respectively), revealed large amounts of unreacted starting borane, seen as a quartet at –20 ppm in the ^11^B NMR. Furthermore, the absence of any C=C bonds in the ^1^H NMR for these entries suggested that full dihydroboration has taken place utilising all C=C bonds.

When the source of borane was changed to diborane(6), produced *in situ*, the same trend was observed (see Table 1). As diborane(6) was prepared *in situ*, two alternative modes of addition were employed, leading to either slow or fast diborane(6) gas production. This was achieved by either adding BF_3_·OEt_2_ to a solution containing both NaBH_4_ and **1**, resulting in a slow release of the desired gas (as the reaction proceeds *via* the formation of the NaBH_4_BH_3_ complex first), or by addition of NaBH_4_ to a solution containing both BF_3_·OEt_2_ and diene **1**, which resulted in the instantaneous release of diborane(6). As in the case of BH_3_·SMe_2_, all reactions yielded a white precipitate, with only NaBF_4_ being observed in the filtrate, appearing at approximately –0.4 ppm in the ^11^B NMR, indicating reaction completion.

As dienes can undergo cationic polymerisation initiated by BF_3_·Lewis base adducts,^35^ a control experiment was carried out to ensure that such a polymerisation was not the cause of the observed white precipitate. Thus, the reaction was performed in the absence of NaBH_4_ (see ESI S6.1†). The presence of BF_3_ alone did not lead to the formation of a white precipitate, confirming that the origin of these solid materials resulted indeed from the dihydroboration reactions.

### 2.1 Analysis and characterisation of the boron-containing precipitates

The solubility of the formed materials was investigated in a range of solvents. These materials, however, were found to be insoluble in acetic acid, acetone, acetonitrile, benzene, chloroform, cyclohexane, DCM, DMF, DMSO, 1,4-dioxane, 1,3-dioxane, ethyl acetate, diethyl ether, hexane and THF under a nitrogen atmosphere, even after being left for a week. Interestingly, these materials reacted with acetonitrile, methanol and triphenylphoshine (solution in diglyme) serving as indirect evidence that boron hydrides are present in these materials (see ESI S.7.1†).^36^

As a result of the insoluble and air-sensitive nature of these materials, only a limited number of characterisation techniques including solid state NMR and FT-IR could be employed. Additionally, basic oxidation of the materials was also carried out. It is worth noting that the mass of these isolated materials was consistently above the maximum expected value (based on a yield of 100%, even after a week under vacuum) indicating the presence of trapped solvents, which was confirmed by solid state NMR. Moreover, this strongly suggested the formation of a polymeric network. Addition of Lewis bases such as PPh_3_ did not fully dissolve the solid material. Future work will further investigate additional reactions that might take place and the possible role of solvent in subsequent reactions.

#### 2.1.1 Analysis by FT-IR

**Fig. 3:**
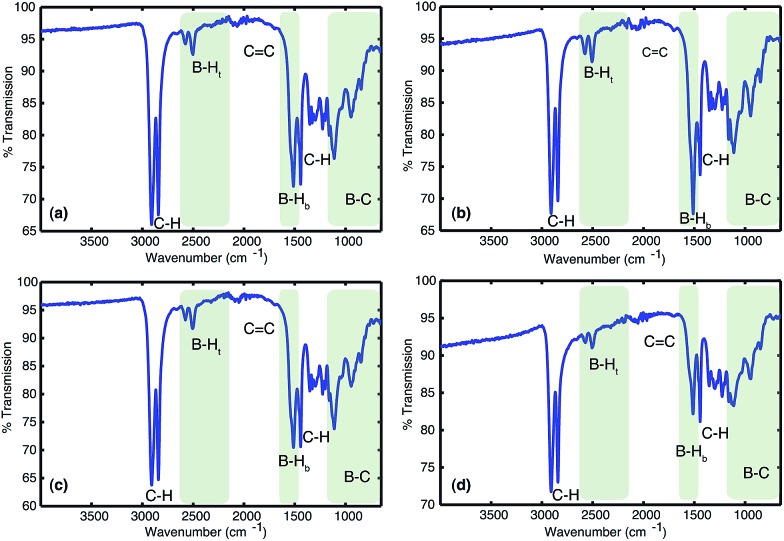
The FTIRs of the materials obtained from the reactions between (a) 1 : 2 molar equivalents of 1,3-cyclohexadiene **1** added to borane BH_3_·SMe_2_, (b) 1 : 1 molar equivalents of 1,3-cyclohexadiene **1** and diborane(6) B_2_H_6_ utilising slow gas release, (c) 2 : 1 molar equivalents of borane BH_3_·SMe_2_ added to 1,3-cyclohexadiene **1** and (d) 1 : 1 molar equivalents of 1,3-cyclohexadiene **1** and diborane(6) B_2_H_6_ utilising fast gas release.

Analysis of the materials by FT-IR under anaerobic conditions indicated the presence of C–H (2900–2800 cm^–1^), terminal B–H_t_ (2600–2500 cm^–1^), bridged B–H_b_ (1600–1500 cm^–1^) and B–C (1200–800 cm^–1^) bonds and the absence of any C=C bonds (1800–1700 cm^–1^). All solids analysed resulted in similar spectra despite originating from a number of different reaction conditions. The FT-IR spectra of four selected materials are shown in Fig. 3, which correspond to the materials also analysed by solid state NMR (*vide infra*). The B–H_t_ bond stretches could indicate either presence of a R_2_BHBH_3_ species (see Fig. 2), which was found to form during the reaction between a (R_2_BH)_2_ species and unreacted borane or diborane(6) starting material, due to R_2_BHBH_2_R or RBH_2_BH_2_R, or unbridged R_2_BH species or even due to BH_3_ trapped inside the material network. The latter option, however, could be dismissed based on solid state NMR data (see Fig. 5c–f). Finally, the absence of any C=C double bonds in both the filtrate and the solid confirmed that full dihydroboration had taken place.

#### 2.1.2 Hydroboration of cyclohexene

**Fig. 4:**
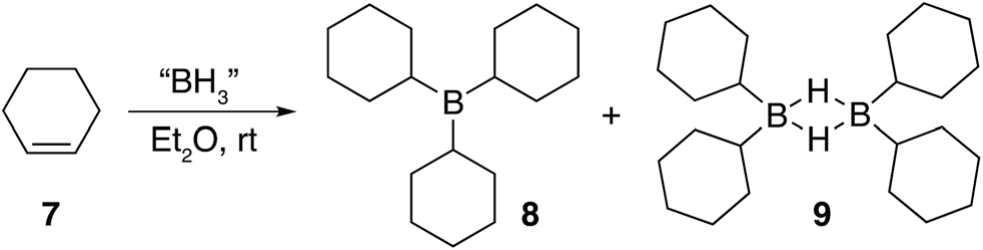
The main products formed during the hydroboration of **7**.

The preparation of several model compounds was required in order to unambiguously assign peaks in the solid state NMR; therefore, cyclohexene **7** was treated with borane resulting in soluble cyclohexyl derivatives **8** and **9** as shown in Fig. 4 (see ESI S.7.2.3†).

Three equivalents of cyclohexene **7** were reacted with a single equivalent of BH_3_·SMe_2_ leading to the formation tricyclohexylborane **8**. However, when the borane was added quickly to **7**, the product ratio significantly favoured the formation of the B–H–B bridged species **9** as confirmed by FT-IR (see ESI S.7.2.1†). Importantly, diethyl ether was chosen as the solvent to prepare model compounds **8** and **9** as opposed to diglyme as it enabled a straightforward method to isolate the two compounds. A white precipitate in ether was readily filtered and isolated to yield dicyclohexyl B–H–B bridged **9**, and confirmed by solution NMR spectroscopy (+29 ppm in ^11^B NMR).^37^ Conversely, tricyclohexylborane **8** remained soluble in ether and could be isolated upon concentration followed by recrystallisation (+81 ppm in solution ^11^B NMR). The dominant formation of dicyclohexylborane **9** (R_2_BH)_2_, especially at the earlier stages of hydroboration, suggested that it was the kinetic product, which could be converted to the thermodynamic tricyclohexylborane **8** over time (see ESI S7.2.3, Fig. S18†), further supported by our theoretical calculations. DFT calculations using the B3LYP/6-311++G** level of theory have shown that in the presence of excess cyclohexene, **9** could hydroborate a third cyclohexene to form tricyclohexylborane **8** although with a high barrier, likely due to the steric effect of the substituent (20.4 kcal mol^–1^, see ESI S11, Table S1†). Interestingly, the powerful stabilisation offered by the B–H–B bridges provides **9** with enough stability in air over short periods of time (*ca.* 5 min) as opposed to the instantly pyrophoric and highly unstable tricyclohexylborane **8**. The direct dependence on the rate of reagent addition towards the formation of kinetic product **9***vs.* thermodynamic product **8** led to materials with different physical properties and reactivities. For example, materials which are mainly consisted of the kinetic products bearing the boron hydride bridges (as opposed to the trialkylboranes, the thermodynamic products) are highly moisture sensitive but not pyrophoric. When these materials where exposed to moisture they transformed from solid to liquid clearly breaking the B–H–B bridges which hold the insoluble polymeric network together. We are currently investigating the applications of these materials especially as instant moisture scavengers.

#### 2.1.3 Analysis by NMR

**Fig. 5:**
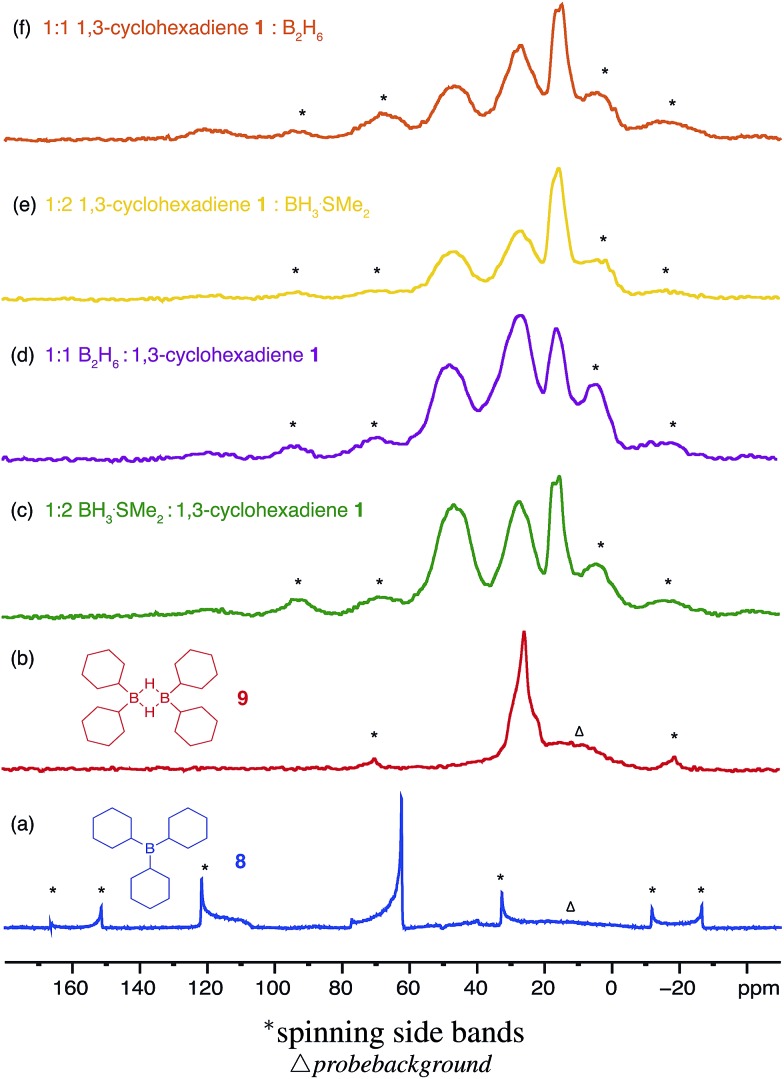
The solid state ^11^B NMR spectra of a range of different materials formed during the hydroboration of 1,3-cyclohexadiene **1** with 1 or 2 equivalents of borane BH_3_·SMe_2_ and diborane(6) B_2_H_6_ gas in diglyme against the model compounds (a) tricyclohexylborane **8** R_3_B and (b) dicyclohexylborane **9** (R_2_BH)_2_.

Solid state ^11^B NMR analysis of model compounds **8** and **9** indeed allowed for deconvolution and assignment of the NMR spectra obtained from the white insoluble materials prepared by hydroboration of cyclohexadiene **1**. As seen in Fig. 5a, the ^11^B resonance from tricyclohexylborane **8** has a relatively broad line shape due to large anisotropic interactions (quadrupolar coupling of 5 MHz and chemical shift anisotropy of –90 ppm determined from a simulation of the spectrum), which are not efficiently reduced by magic angle spinning at 10 kHz. The isotropic chemical shift of +82 ppm fits well with the value measured in solution ^11^B NMR (see ESI S7.3, Fig. S21†). On the other hand, the ^11^B resonance of dicyclohexylborane **9** shown in Fig. 5b, has a much narrower line shape, which can be fitted with quadrupolar coupling of about 2.7 MHz and an isotropic chemical shift of +30 ppm (a good fit with its solution resonance at +29 ppm). It is clear from their ^11^B NMR spectra (Fig. 5c–f) that the boron species present in the insoluble materials are similar, with the only difference being their relative abundance serving as additional evidence for the formation of a network polymer. The absence of any significant amount of a trisubstituted borane species was expected on account of the relatively fast reagent addition used during the preparation of these materials.

The remaining signals appearing at approximately +50 and +18 ppm most likely correspond to other boron hydride species such as R_2_BHBH_3_ and R_2_BHBH_2_R (Fig. 2). This is further supported by the effect on the NMR signal intensities when the solid state ^11^B NMR is acquired with and without ^1^H decoupling (see ESI S.7.3, Fig. S20†), as well as by FT-IR and circumstantially by the reactivity of these materials with Lewis bases. Additionally, the presence of trapped diglyme solvent observed in the solid state ^1^H and ^13^C NMR spectra (see ESI S.7.3, Fig. S22 and S23†), lends strong support to the formation of polymeric networks. Most importantly, the absence of any unreacted C=C bonds critically points to quantitative dihydroboration of **1** using a variety of borane sources and reaction conditions.

#### 2.1.4 Analysis of the oxidised products by GC-MS

**Fig. 6:**
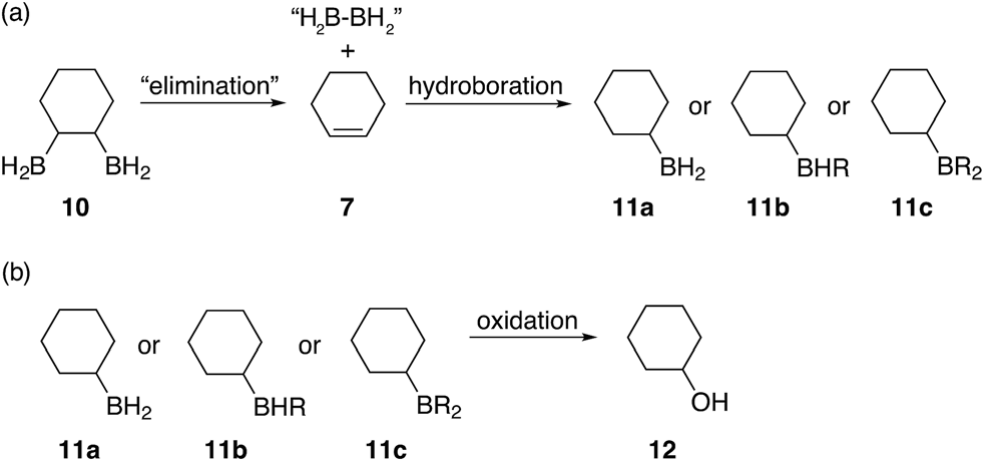
Proposed elimination mechanism for the formation of cyclohexanol when the boron containing polymers are oxidised.

Basic oxidation of these insoluble materials with hydrogen peroxide, adapted from literature procedures^1^ and subsequent analysis of the oxidation products by GC-MS revealed the statistical formation of all expected cyclohexane diols, shown in Fig. 2, as the main products. Interestingly, the formation of considerable amounts of cyclohexanol **12** was observed in the absence of any unsaturated C=C bonds. The formation of this alcohol could be attributed to a diborane elimination side reaction similar to what is shown in Fig. 6. However, contrary to what was proposed by A. Hassner and B. H. Braun,^38^ we believe that such an elimination of 1,2-diboranes, such as **10**, is highly unlikely in the absence of a driving force, such as a Lewis base. Perhaps, SMe_2_, THF (if present), diglyme or even a species formed during the reaction could mediate such an elimination. Protonolysis of the α-boron atom, in the 1,2-disubstituted diborane case, could also possibly lead to the formation of an oxidised alkylborane which resembles a carboxylic acid ([(RO)_2_B(OR)OH]^–^) thus eliminating one of the boron atoms and replacing it with a hydride. However, we believe that this is unlikely due to the large amounts of cyclohexanol **12** observed in some cases and the highly uncontrolled nature of this hydroboration system leading to a statistical mixture of 1,2-, 1,3- and 1,4-substitution patterns. Furthermore, during our studies we observed that β-hydride elimination of diboranes is possible, however, such an elimination followed by oxidation would not result in the formation of cyclohexanol but rather the unsaturated compound 2-cyclohexene-1-ol **2**. Finally, elimination reactions caused by oxidation would also produce alcohols with C=C double bonds rather than cyclohexanol and therefore must also be discounted. We are currently further investigating the mechanism of this elimination.

Several attempts were made to avoid the formation of the insoluble precipitates including lowering the reaction temperature (–78 °C), decreasing the concentration, or carrying out the reaction in non-ethereal solvents, known to considerably slow down the rate of hydroboration, such as DCM.^39^ Nevertheless, the insoluble materials formed in all cases, albeit at different rates, even when DCM was used as the solvent. The retarded rate of formation of these materials in DCM further suggested that a hydroboration cascade mechanism was indeed likely leading to chains and three dimensional networks as shown in Fig. 2.^39^ A number of additional observations further supported our hypothesis that the isolated materials were indeed borane-containing network polymers. The difunctional nature of **1** combined with the trifunctional nature of BH_3_ and the energetic stabilisation gained from the formation of B–H–B bridges is akin to an A_2_ + B_3_ step-growth polymerisation leading to insoluble, crosslinked networks. Moreover, the solids readily transform into liquids upon exposure to air, further suggesting that oxidation leads to facile disruption of the borane-hydrocarbon polymeric network. Finally, the dependence of the materials' physical appearance on the reaction solvent (glassy in THF and a white powder in diglyme) together with the high solvent content present in these materials are also indicative of the formation of a polymer network.

### 2.2 Proposed mechanism for the formation of the boron containing polymers

**Fig. 7:**
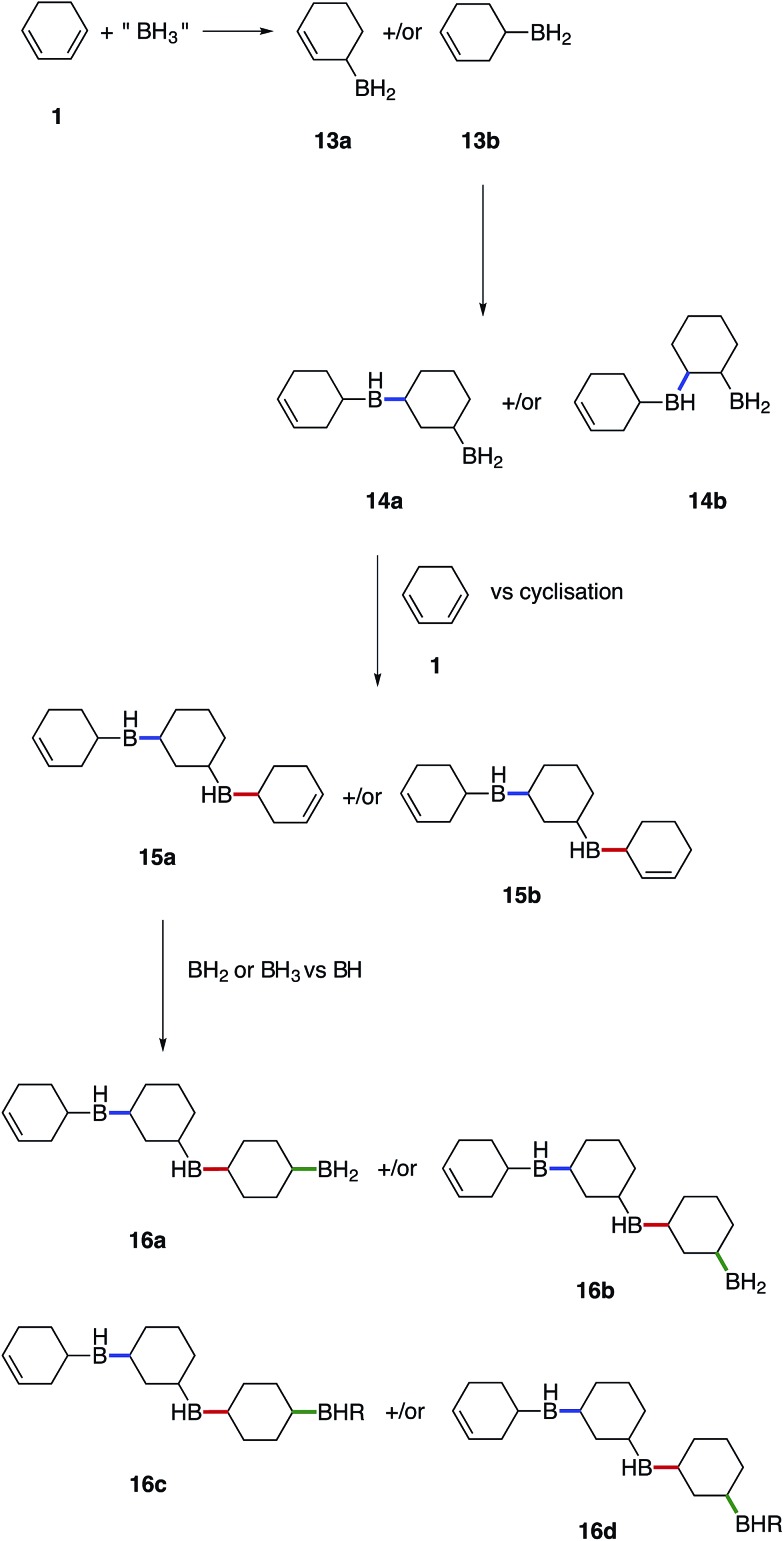
Proposed mechanism for the formation of insoluble boron containing hydrocarbons.

We believe that these materials are polymeric species formed *via* a cascade of hydroboration reactions. Detailed inspection of the boron species formed during hydroboration by NMR gave rise to the following proposed mechanism shown in Fig. 7. Initially, **1** undergoes monohydroboration to form either an allyl RBH_2_ species **13a** or homoallyl RBH_2_ species **13b**. The ratio of **13a** to **13b** is likely to be highly dependent on the particular diene structure and is beyond the scope of this study. However, it was expected that the allyl RBH_2_ species **13a** is slightly more stable compared to the homoallyl **13b** on account of conjugation with the C=C bond. Indeed, during our calculations we found that the BH_2_ group is stabilised in an axial position, when SMe_2_ is absent, by interactions with the adjacent double bond that partly delocalises to the boron 2p orbital (see ESI S11, S53†). However, the TS_2_ barrier towards both **13a** and **13b** is almost identical, which cannot explain the selectivity. It is possible that the overall dynamics may determine the product selectivity as reported previously for the hydroboration of propene.^40^ Alternatively, the hydroboration product can rearrange and the BH_2_ group can isomerise, forming the more stable **13a** axial product starting from the equatorial **13b** isomer. Once **13a** forms an adduct with SMe_2_ (or other electron donor molecules), the equatorial conformation is preferred. It is worth noting, that the formed RBH_2_ species most likely exist as dimers, which contain B–H–B bridges providing substantial stabilisation,^41^ however, for simplicity all borane species depicted in the proposed mechanism are drawn with terminal B–H bonds.

Once hydroboration occurs, both the monohydroboration and dihydroboration species are much more reactive than the starting materials, conjugate diene **1** and borane. This was especially evident on account of the unreacted borane observed in solution after the consumption of all C=C bonds, entries 2–4 in Table 1. Additionally, monohydroboration of **1** leads to the formation of an isolated C=C bond, which was observed to be highly reactive (at least under our conditions) compared to the conjugated starting material **1**, leading to a cascade of hydroboration reactions and concomitant formation of a cross-linked polymeric network as the monohydroboration species continue to react preferentially.

In general terms, hydroboration on double bonds is known to follow a two-step process.^40^ The first and usually rate-limiting step is the activation of BH_3_ (or substituted RBH_2_ species) by breaking the solvent–borane adduct or the borane dimer. The second step is the addition of the BH_3_ to the double bond and usually has a smaller barrier.^40^ Our calculations suggest that the formation of the solvent-free BH_3_ intermediate is stabilized by short-lived intermediate complexes formed with the C=C bond containing reaction partner. We found that the barrier of the first step is reduced from +26.1 kcal mol^–1^ to +14.6 kcal mol^–1^ using BH_2_-cyclohexene, to +15.0 kcal mol^–1^ using 1,3-cyclohexadiene **1**, and to +13.8 kcal mol^–1^ using cyclohexene (see ESI S11, Table S1†). It was evident that the energy difference between mono and dihydroboration is very small and therefore, the determining factor for the preferred reaction pathway is most likely influenced by the experimental conditions. The barrier of the first step to form solvent-free boranes is also significantly decreased when substituted boranes (RBH_2_) are used with electron-donating R groups, even when the free RBH_2_ is not further stabilised by adduct formation with a solvent molecule or a C=C bond. With R = cyclohexyl (or cyclohexenyl), the barrier to generate a free BRH_2_ is +13.3 kcal mol^–1^ (or +13.1 kcal mol^–1^), which is +12.7 kcal mol^–1^ (or +13.0 kcal mol^–1^) smaller than for the dissociation of the BH_3_·SMe_2_ complex (see ESI S11 Table S2†). As a result, the formed monohydroboration products are expected to react further.

Subsequent hydroboration of **13a** or **13b** can take place *via* several different pathways, depending on the reaction conditions. The newly formed, solvent-free monoalkylboranes (**13a** or **13b**) can further react rapidly and hydroborate another cyclohexadiene molecule (Δ*E*^‡^ = 1.0–2.2 kcal mol^–1^, see ESI S11 Table S3†), or other monocyclohexylboranes (Δ*E*^‡^ = 4.0–4.3 kcal mol^–1^, see ESI S11 Table S3†) leading to polymer formation. When excess borane is still available, BH_3_ can also hydroborate the remaining double bond on **13a** or **13b**, although with a somewhat higher energy barrier. For example, the barrier for the hydroboration of **13a** is 17.7–18.3 kcal mol^–1^ (see ESI S11, Table S1†). Interestingly, this second hydroboration can also take place *via* an intramolecular mechanism through a RH_2_B–BH_3_ complex (ESI S11, Fig. S54†). The hydroboration reaction within this complex has some of the lowest barriers, 15.5 kcal mol^–1^ to form the kinetically favoured *cis*-1,2-diborane cyclohexane isomer (or 18.0 kcal mol^–1^ to form the thermodynamically favored *cis*-1,3-diborane cyclohexane, see ESI S11, Table S1†). The formed diboranes are further stabilised *via* B–H–B bridges. Potentially, this barrier is further reduced by forming a complex with another electron donating molecule, such as SMe_2_, that facilitates the release of an active BH_3_ (results are not shown). It is worth noting that the second hydroboration reaction can produce eight distinct diborane cyclohexane isomers in general if the chair-boat conformational change is not hindered, and many more in the conformationally restrained polymers. The formation of each isomer has different potential reaction mechanisms and corresponding rates. Interestingly, the intermolecular hydroboration, with the bridged *cis*-1,2-cyclohexane (see Fig. 6), is the kinetically favoured mechanism to form diborane over most of the other possibilities listed above (see ESI S11, Table S1†). The thermodynamically most stable product is the axial *cis*-1,3-cyclohexane, due to the favourable positions of the BH_2_ groups of this isomer to form B–H–B bridges. This intermolecular mechanism may provide an explanation of the relative abundance of cyclohexanol after basic oxidation of these materials, assuming a potential subsequent elimination reaction from the bridged diborane species (see Fig. 6).

Boron containing polymer formation from dienes has been reported previously by Brown *et al.* during the hydroboration of 1,3-butadiene.^27^–^29^,^42^ However, these polymers were found to form *via* cyclic hydroboration, which is not expected in the case of cyclic diene **1**, as this would require the initial formation of highly unfavoured ring-in-ring structures (see ESI S8, Fig. S48†). As a result, cyclic hydroboration mechanisms and their products are not considered here.

### 2.3 Dihydroboration of other cyclic dienes

Intrigued by the results of our study on 1,3-cyclohexadiene, we expanded the substrate scope to include a range of different cyclic dienes including: α-terpinene, 1,3-cycloheptadiene, 1,3-cyclooctadiene, 1,3,5,5-tetramethyl-1,3-cyclohexadiene, γ-terpinene, 1,4-cyclohexadiene, 1,2,4,5-tetramethyl-1,4-cyclohexadiene, 1,5-cyclooctadiene and the acyclic 2,3-dimethyl-1,3-butadiene as a control and direct comparison to the reported polymer structures (see ESI S9, Fig. S49†). In a similar fashion to our observation with **1**, all of the dienes listed above, including the acyclic diene, were susceptible to dihydroboration. Not all dienes above led to the formation of crosslinked polymeric networks, several resulted in a clear solution, which contained a mixture of boron hydride species and unreacted borane (see ESI S9, Fig. S50 and S51†). A clear pattern, however, was observed with simple cyclic dienes, such as **1**, 1,3-cycloheptadiene, 1,3-cyclooctadiene, and 1,4-cyclohexadiene leading to the formation of the boron-containing crosslinked polymers whereas more sterically demanding dienes such as α-terpinene yielded a clear solution. Interestingly, γ-terpinene also led to the formation of a cross-linked material. Following this trend, hydroboration of 1,2,4,5-tetramethyl-1,4-cyclohexadiene yielded clear solutions while the reaction with 1,3,5,5-tetramethyl-1,3-cyclohexadiene first produced a clear solution, but observation of an insoluble material occurred after 2 h. Despite the absence of sterically demanding substituents, hydroboration of 1,5-cyclooctadiene resulted in a clear solution, yielding the commonly used reagent 9-BBN on account of the formation of a stable 6-member boron-containing cyclic structure. Finally, when acyclic diene, 2,3-dimethyl-1,3-butadiene was used in the reaction, a clear solution was obtained as previously reported.^27^–^29^ Therefore, it was clear that the position of the C=C bond, in relation to the sterically demanding group, was crucial in the formation of these materials, which were not limited by ring size.

## 3 Conclusions

To conclude, boron containing polymers form when cyclic 1,3- or 1,4-dienes, free of sterically demanding groups are fully hydroborated, irrespective of the cyclic diene size, borane equivalents and mode of addition. When steric hindrance is present or cyclic borane species are favourably formed, as for example when straight chain of 1,5-dienes are utilised, clear solutions are observed. These materials were characterised by solid state FT-IR and NMR and were found to consist of boron hydride bridged species due to stabilisation reasons and the synthetic time scale utilised. Further supported by oxidation analysis, it was clear that hydroboration proceeds further than the monosubstituted borane adducts to form higher substituted, more stable, bridged borane species. Therefore, our results are in disagreement with the published conclusions on the study of dihydroboration of cyclic dienes by H. C. Brown *et al.*^24^,^25^ as we observed that dihydroboration of cyclic rings proceeds quantitatively irrespective of ring size or structure.

## Supplementary Material

Supplementary information

Supplementary information

Supplementary information

Supplementary information

Supplementary information

Supplementary information

Supplementary information
