# Supplementary figures and images for: Divergence from the classical hydroboration reactivity; boron containing materials through a hydroboration cascade of small cyclic dienes
Source: Chem Sci. 2015 Aug 6;6(11):6262–9. doi: 10.1039/c4sc02729a (PMC6054143; doi:10.1039/c4sc02729a)

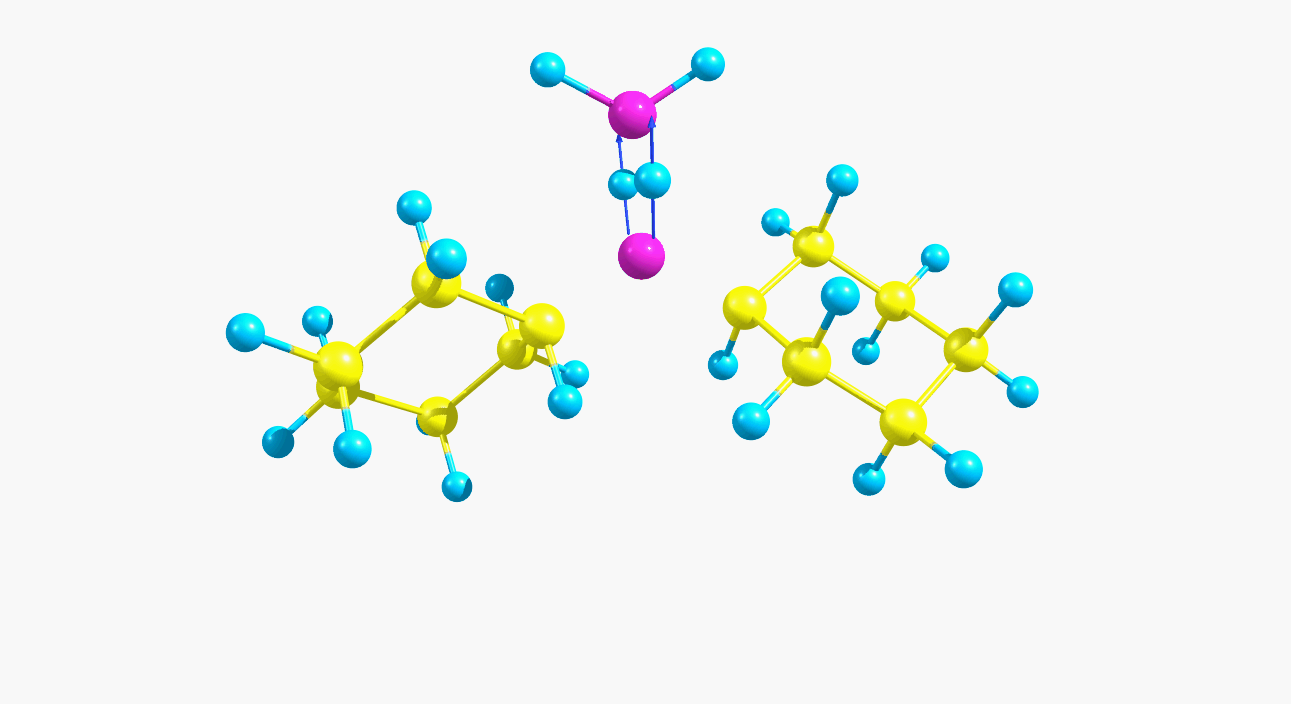

Supplement: Supplementary file 1 [file SC-006-C4SC02729A-s001.gif]

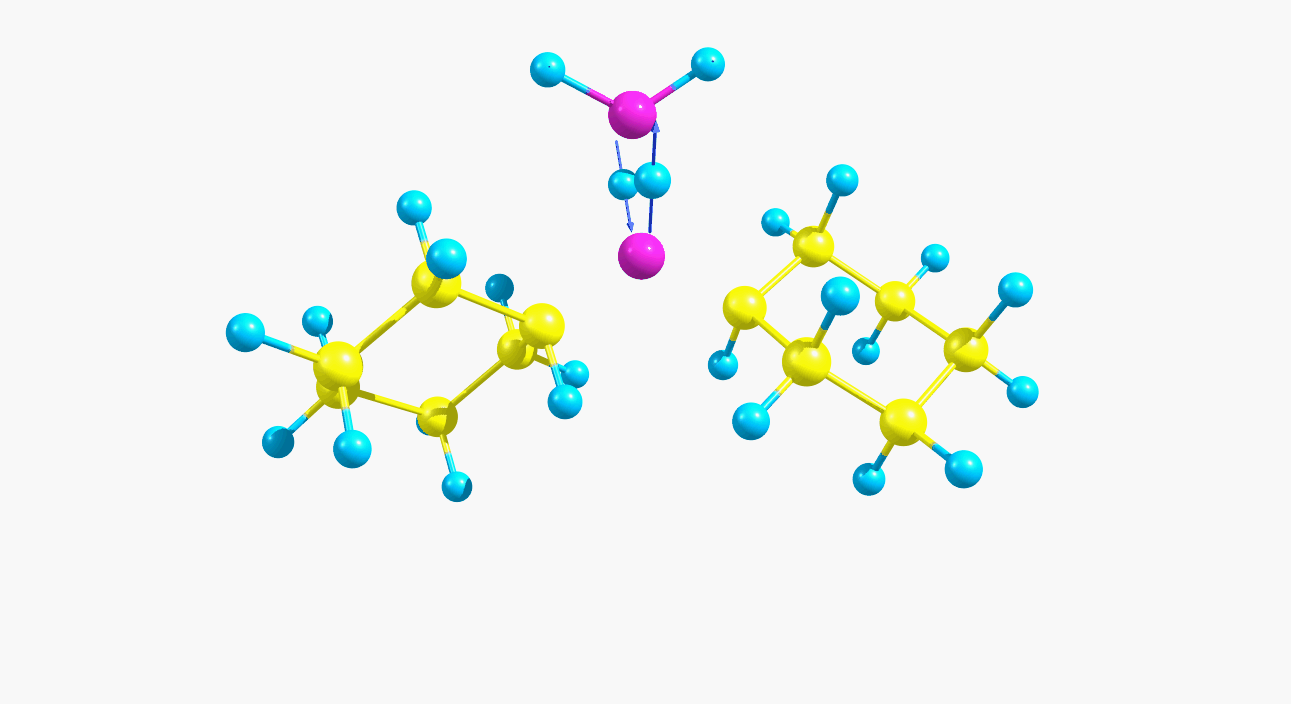

Supplement: Supplementary file 2 [file SC-006-C4SC02729A-s002.gif]

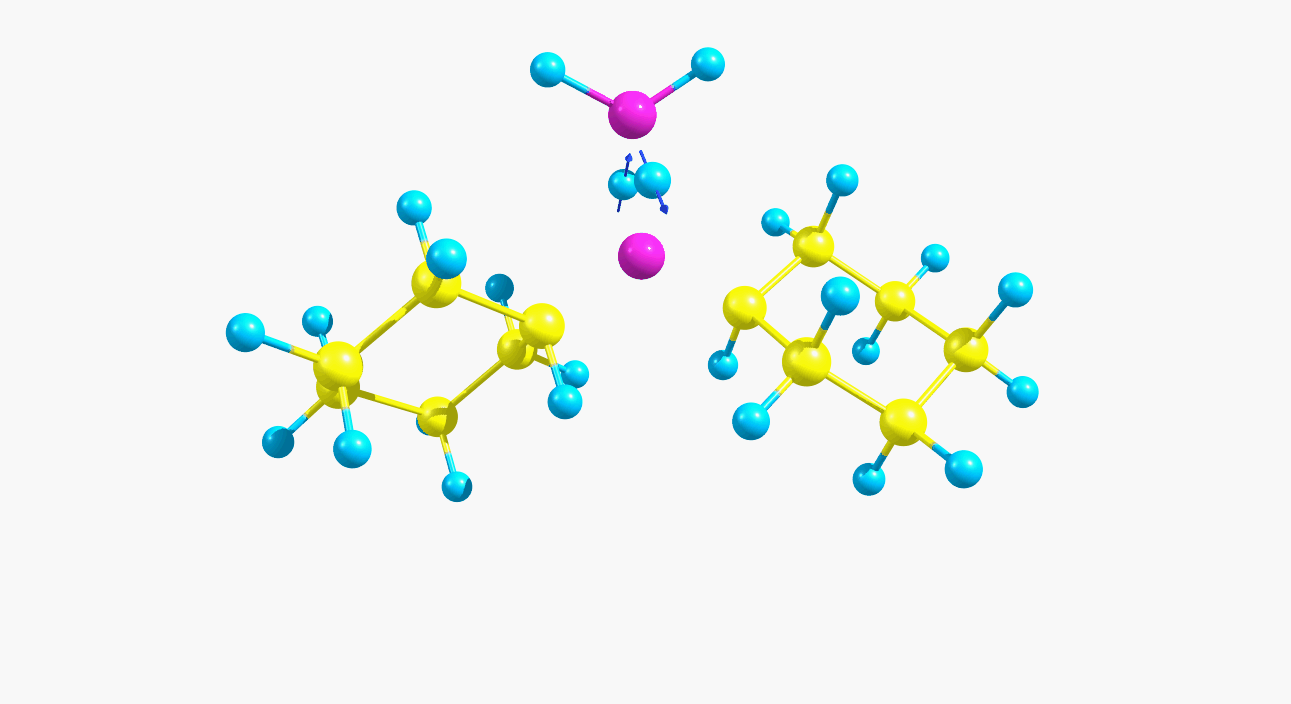

Supplement: Supplementary file 3 [file SC-006-C4SC02729A-s003.gif]

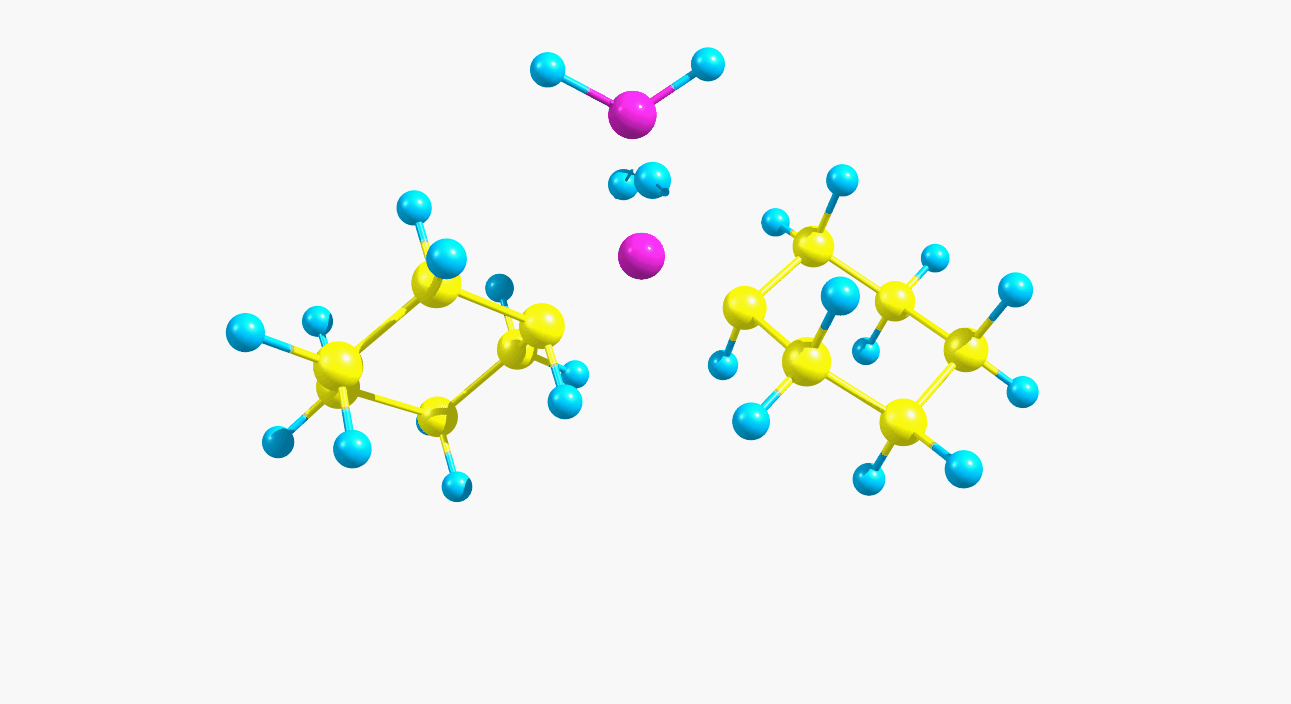

Supplement: Supplementary file 4 [file SC-006-C4SC02729A-s004.gif]

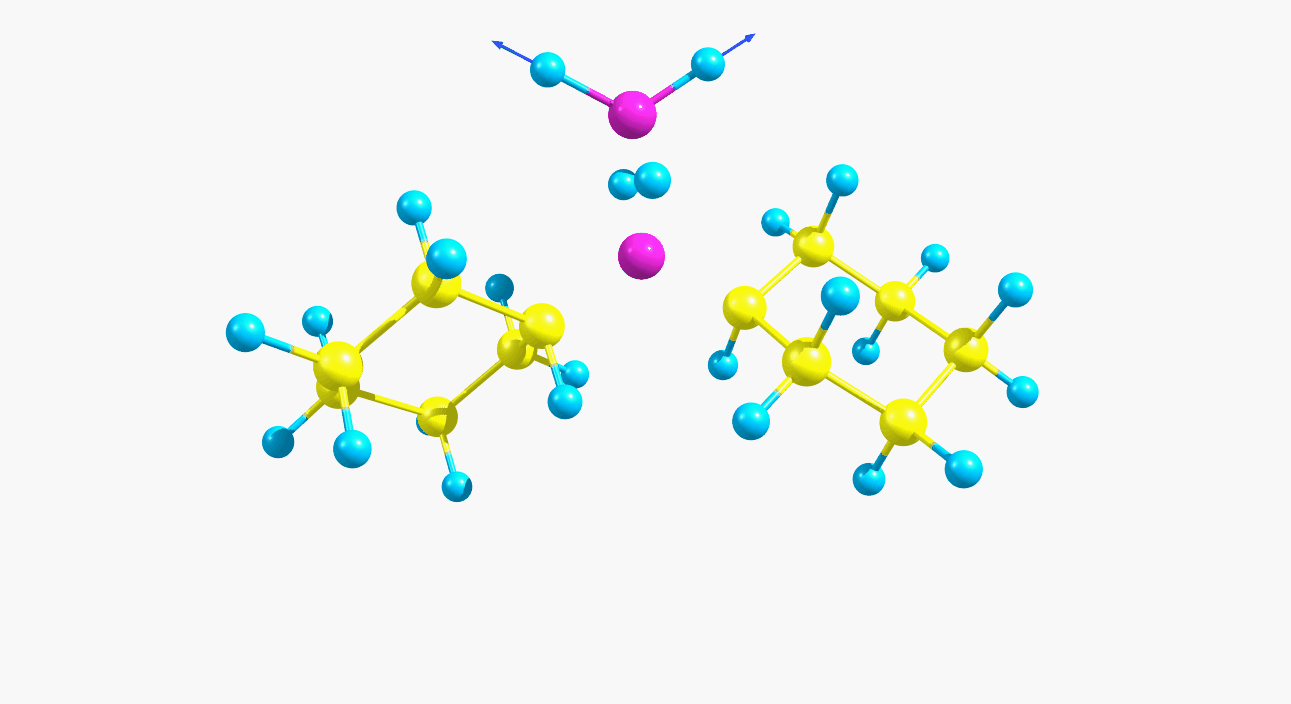

Supplement: Supplementary file 5 [file SC-006-C4SC02729A-s005.gif]

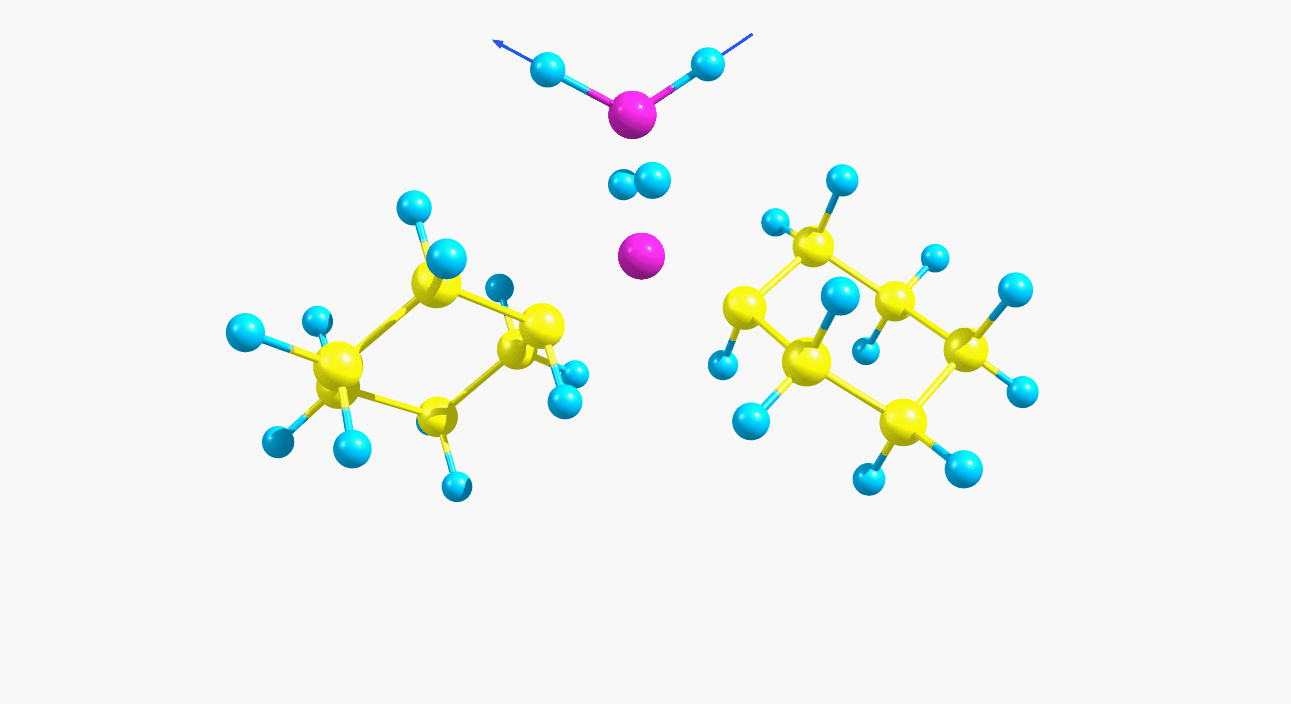

Supplement: Supplementary file 6 [file SC-006-C4SC02729A-s006.gif]
