# Supplementary material for: Divergence from the classical hydroboration reactivity; boron containing materials through a hydroboration cascade of small cyclic dienes
Source: Chem Sci. 2015 Aug 6;6(11):6262–9. doi: 10.1039/c4sc02729a (PMC6054143; doi:10.1039/c4sc02729a)
Supplement: Supplementary file 7 [file SC-006-C4SC02729A-s007.pdf]

Electronic Supplementary Material (ESI) for Chemical Science.
This journal is © The Royal Society of Chemistry 2015

Divergence from the classical hydroboration reactivity; boron containing materials through a hydroboration cascade of small cyclic dienes

Anna Andreou[1], Michal Leskes[2], Pablo G. Jambrina[3], Gary J. Tustin[4], Clare P. Grey[2], Edina Rosta[3], and Oren A. Scherman*[1]

[1]Melville Laboratory for Polymer Synthesis, Department of Chemistry, University of Cambridge, Lensfield Road, Cambridge CB2 1EW, UK. Email: oas23@cam.ac.uk
[2]Department of Chemistry, University of Cambridge, Lensfield Road, Cambridge CB2 1EW, UK
[3]Department of Chemistry, Kings College London, Strand, London, WC2R 2LS, UK
[4]Schlumberger Cambridge Research, High Cross, Madingley Road, Cambridge UK, CB3 0EL

S.1 Instrumentation and Materials

All experiments were carried out under nitrogen, using oven dry glassware and dry solvents, unless stated otherwise. Air sensitive chemistry was carried out in a Braun Technology Glovebox (MBraun UniLab) under a nitrogen atmosphere.

Solvents THF, acetonitrile, DCM, diethyl ether, 1,4-dioxane, acetone, DMF, chloroform, DMSO, hexane and methanol were purchased from Acros. Diglyme, 1,3-dioxane, dimethyl sulfide, acetic acid were purchased to their highest purity from Sigma Aldrich. Glyme and benzene were purchased from Alfa Aesar. All were purchased to the highest available purity (extra dry when available) and were used as received. Sodium borohydride, boron trifluoride, cyclohexene, dicyclopentadiene, 1,3-cyclooctadiene, 1,3-dimethyl-1,3-butadiene and 1,3,5,5-tetramethyl-1,3-cyclohexadiene were purchased from Alfa Aesar to the highest available purity and were used as received. α-terpinene, 1,3-cyclohexadiene, 1,4-cyclohexadiene, 1,2,4,5-tetramethyl-1,4-cyclohexadiene, γ-terpinene, 1,3-cycloheptadiene, triphenylphosphine and sodium borodeuteride 98% D atom were purchased from Sigma Aldrich to the highest available purity and were used as received 1,5-cyclooctadiene was purchased from Acros and cyclohexane was purchased from Breckland Scientific. Both were obtained to the highest available purity and used as received. 1,3-cyclopentadiene was synthesised, and utilised immediately, by cracking of dicyclopenatadiene as reported in the literature.[1] Commercially available borane solutions, $BH_3{\cdot}THF$ and $BH_3{\cdot}SMe_2$, of different concentrations, were trialled from a range of different commercial companies including Sigma Aldrich and Alfa Aesar. Deuterated NMR solvents were purchased from Euriso-top as ampoules and were used as received.

The ^{1}H, ^{11}B, ^{13}C and ^{31}P NMR spectra were recorded on a Bruker Advance BB-ATM-500 MHz NMR spectrometer. ^{1}H and ^{13}C spectra were recorded using TMS as a reference. ^{11}B spectra were recorded using $BF_3{\cdot}OEt_2$ as a reference and ^{31}P NMR spectra were recorded using 85% H_3PO_4 in D_2O as a reference. Solid state NMR experiments were performed on a 16.4 T Bruker 700 AVANCE III spectrometer using a 4 mm triple resonance probe. All experiments were performed with a spinning frequency of 10 kHz. ^{11}B spectra were referenced with respect to 0.1M boric acid (19.6 ppm). A rotor synchronized echo sequence was used with a non-selective 90° pulse excitation (nutation frequency equal to 95 kHz) and a selective 180° pulse (nutation

frequency equal to 12 kHz). Proton decoupling was applied during signal acquisition using SPINAL64[2] with a nutation frequency of 70 kHz. The relaxation delay was optimized for each sample and varied between 2-5s. ^{1}H spectra were referenced to adamantane (1.8 ppm) and acquired with a rotor synchronized Hahn echo using pulse amplitudes equal to 85 kHz. ^{13}C spectra were references to adamantane (CH at 38.5 ppm). ^{1}H-^{13}C cross polarization experiments were acquired with a contact time of 500 μs and 2 ms and a relaxation delay of 5s and 10s for the model compounds and insoluble reaction products respectively. ^{11}B line shape simulations were performed using the SOLA tool in TOPSPIN. NMR data are reported as follows: chemical shift, integration, multiplicity (s = singlet, d = doublet, t = triplet, q = quartet, br = broad, m = multiplet), coupling constants (Hz) and assignment. All chemical shift values are reported in parts per million (ppm).

Infrared spectra were recorded on a Bruker ALPHA FT-IR instrument placed inside a nitrogen filled glove box. Controlled additions were performed using a Precidor 5003 infusion syringe pump.

S.2 Experimental Protocols

S.2.1 Hydroboration of alkenes

Representative procedure for the hydroboration of cyclohexene: Borane dimethylsulfide complex (0.07 mL, 1.0507 mmol) was added dropwise to a solution of cyclohexene (0.07 mL, 1.0507 mmol) dissolved in pre-cooled, to -40 ^{o}C, THF (1 mL). The reaction mixture was left to stir under nitrogen, at rt (or 0 ^{o}C), for 1h to give a clear solution.

Representative procedure for the hydroboration of cyclohexene in THF utilising the borane to alkene addition mode: Borane dimethylsulfide complex (various amounts) was added dropwise to a solution of cyclohexene (0.07 mL, 1.0507 mmol) dissolved in pre-cooled, to -40 ^{o}C, THF (1 mL). The reaction mixture was left to stir under nitrogen, at rt, for 1h to give a clear solution containing a white precipitate.

Borane dimethylsulfide complex various amounts = (0.02 mL, 0.3467 mmol), (0.04 mL, 0.5254 mmol), (0.07 mL, 1.0507 mmol), (0.14 mL, 2.1014 mmol).

Representative procedure for the hydroboration of cyclohexene in THF utilising the alkene to borane addition mode: Cyclohexene (0.07 mL, 1.0507 mmol) was added dropwise to a solution of borane dimethylsulfide complex (various amounts) dissolved in pre-cooled, to -40 ^{o}C, THF (1 mL). The reaction mixture was left to stir under nitrogen, at rt, for 1h to give a clear solution containing a white precipitate.

Borane dimethylsulfide complex various amounts = (0.02 mL, 0.3467 mmol), (0.04 mL, 0.5254 mmol), (0.07 mL, 1.0507 mmol), (0.14 mL, 2.1014 mmol).

Representative procedure for the hydroboration of cyclohexene with diborane(6) gas, in diglyme: Cyclohexene (0.07 mL, 1.0507 mmol) and sodium borohydride (0.1569g, 4.1475 mmol) were dissolved in pre-cooled, to -40 ^{o}C, diglyme (3.2 mL). Boron trifluoride diethyl ether complex (0.69 mL, 5.4936 mmol) was then added dropwise and the resulting reaction mixture was left to stir under nitrogen, at rt, for 1h to give a clear solution containing a white precipitate.

S.2.2 Kinetic study

Representative procedure for the kinetic study of hydroboration of cyclohexene in diethyl ether, utilising the borane to alkene addition mode: Borane dimethylsulfide complex (0.77 mL, 8.1197 mmol) was added dropwise over x minutes to a solution of cyclohexene (2.47 mL, 0.02438 mol) dissolved in diethyl ether (5 mL). The reaction mixture was left to stir under nitrogen, at rt, for 1h to give a clear solution containing a white precipitate.

Time x in minutes = 0, 1, 5, 10.

Representative procedure for the kinetic study of hydroboration of cyclohexene in diethyl ether, utilising the alkene to borane addition mode: Cyclohexene (2.47 mL, 0.02438 mol) was added dropwise over x minutes to a solution of borane dimethylsulfide complex (0.77 mL, 8.1197 mmol) dissolved in diethyl ether (5 mL). The reaction mixture was left to stir under nitrogen, at rt, for 1h to give a clear solution containing a white precipitate.

Time x in minutes = 0, 1, 5, 10.

S.2.3 Hydroboration of dienes

Representative procedure for the hydroboration of dienes using borane tetrahydrofuran complex utilising the borane to diene addition mode: Borane tetrahydrofuran complex (0.74 mL, 1M, 0.7340 mmol or 1.47 mL, 1M, 1.4680 mmol or 2.22 mL, 1M, 2.2020 mmol) was added dropwise to a solution of a diene dissolved in pre-cooled, to -40 ^{o}C, THF (1 mL). The reaction mixture was left to stir under nitrogen, at rt (or 0 ^{o}C), for 1h to give a clear solution which depending on the diene used, contained a white precipitate.

Diene = α-terpinene (0.12 mL, 0.7340 mmol), 1,3-cyclohexadiene (0.07 mL, 0.7340 mmol), 1,3-cyclopentadiene (0.06 mL, 0.7340 mmol), 1,3-cyclooctadiene (0.09 mL, 0.7340 mmol), 1,4-cyclohexadiene (0.07 mL, 0.7340 mmol), 2,3-dimethyl-1,3-butadiene (0.09 mL, 0.7340 mmol).

Representative procedure for the hydroboration of dienes using borane dimethyl sulfide complex utilising the borane to diene addition mode in THF: Borane dimethylsulfide complex (0.14 mL, 1.4680 mmol) was added dropwise to a solution of a diene dissolved in pre-cooled, to -40 ^{o}C, THF (1 mL). The reaction mixture was left to stir under nitrogen, at rt (or 0 ^{o}C), for 1h to give a clear solution which depending on the diene used, contained a white precipitate.

Diene = α-terpinene (0.12 mL, 0.7340 mmol), γ-terpinene (0.12 mL, 0.7340 mmol), 1,3-cyclohexadiene (0.07 mL, 0.7340 mmol), 1,3-cyclooctadiene (0.09 mL, 0.7340 mmol), 1,3,5,5-tetramethyl-1,3-cyclohexadiene (0.13 mL, 0.7340 mmol), 1,4-cyclohexadiene (0.07 mL, 0.7340 mmol), 1,2,4,5-tetramethyl-1,4-cyclohexadiene (0.1g, 0.7340 mmol), 1,5-cyclooctadiene (0.12 mL, 0.7340 mmol), 2,3-dimethyl-1,3-butadiene (0.09 mL, 0.7340 mmol).

Representative procedure for the hydroboration of dienes using borane dimethyl sulfide complex utilising the borane to diene addition mode in diglyme: Borane dimethylsulfide complex (0.07 mL, 0.7340 mmol or 0.14 mL, 1.4680 mmol) was added dropwise to a solution of a diene dissolved in diglyme (3.2 mL). The reaction mixture was left to stir under nitrogen, at rt (or 0 ^{o}C), for 1h to give a clear solution which depending on the diene used, contained a white precipitate.

Diene = α-terpinene (0.12 mL, 0.7340 mmol), γ-terpinene (0.12 mL, 0.7340 mmol), 1,3-cyclohexadiene (0.07 mL, 0.7340 mmol), 1,3-cycloheptadiene (0.10 mL, 0.7340 mmol), 1,3-cyclooctadiene (0.09 mL, 0.7340 mmol), 1,3,5,5-tetramethyl-1,3-cyclohexadiene (0.13 mL, 0.7340 mmol), 1,4-cyclohexadiene (0.07 mL, 0.7340 mmol), 1,2,4,5-tetramethyl-1,4-cyclohexadiene (0.1g, 0.7340 mmol), 1,5-cyclooctadiene (0.12 mL, 0.7340 mmol), 2,3-dimethyl-1,3-butadiene (0.09 mL, 0.7340 mmol).

Representative procedure for the hydroboration of α-terpinene in THF utilising the borane to diene addition mode: Borane dimethylsulfide complex (various amounts) was added dropwise to a solution of α-terpinene (0.12 mL, 0.7372 mmol) dissolved in pre-cooled, to -40 ^{o}C, THF (1 mL). The reaction mixture was left to stir under nitrogen, at rt, for 1h to give a clear solution.

Borane dimethylsulfide complex various amounts = (0.04 mL, 0.3686 mmol), (0.07 mL, 0.7372 mmol), (0.14 mL, 1.4744 mmol).

Representative procedure for the hydroboration of α-terpinene in THF utilising the diene to borane addition mode: α-terpinene (0.12 mL, 0.7372 mmol) was added dropwise to a solution of borane dimethylsulfide com-

plex (various amounts) dissolved in pre-cooled, to -40 °C, THF (1 mL). The reaction mixture was left to stir under nitrogen, at rt, for 1h to give a clear solution.

Borane dimethylsulfide complex various amounts = (0.04 mL, 0.3686 mmol), (0.07 mL, 0.7372 mmol), (0.14 mL, 1.4744 mmol).

Representative procedure for the hydroboration of α-terpinene in diglyme utilising the borane to diene addition mode: Borane dimethylsulfide complex (various amounts) was added dropwise to a solution of α-terpinene (0.12 mL, 0.7372 mmol) dissolved in pre-cooled, to -40 °C, diglyme (3.2 mL). The reaction mixture was left to stir under nitrogen, at rt, for 1h to give a clear solution.

Borane dimethylsulfide complex various amounts = (0.04 mL, 0.3686 mmol), (0.07 mL, 0.7372 mmol), (0.14 mL, 1.4744 mmol).

Representative procedure for the hydroboration of α-terpinene in diglyme utilising the diene to borane addition mode: α-terpinene (0.12 mL, 0.7372 mmol) was added dropwise to a solution of borane dimethylsulfide complex (various amounts) dissolved in pre-cooled, to -40 °C, diglyme (3.2 mL). The reaction mixture was left to stir under nitrogen, at rt, for 1h to give a clear solution.

Borane dimethylsulfide complex various amounts = (0.04 mL, 0.3686 mmol), (0.07 mL, 0.7372 mmol), (0.14 mL, 1.4744 mmol).

Representative procedure for the hydroboration of α-terpinene in different solvents utilising the borane to diene addition mode: Borane dimethylsulfide complex (0.14 mL, 1.4744 mmol) was added dropwise to a solution of α-terpinene (0.12 mL, 0.7372 mmol) dissolved in solvent (1 mL). The reaction mixture was left to stir under nitrogen, at rt, for 1h to give a clear solution.

Solvent = THF, diglyme, glyme, diethyl ether, 1,3-dioxane, 1,4-dioxane.

Representative procedure for the hydroboration of 1,3-cyclohexadiene in THF utilising the borane to diene addition mode: Borane dimethylsulfide complex (various amounts) was added dropwise to a solution of 1,3-cyclohexadiene (0.07 mL, 0.7372 mmol) dissolved in pre-cooled, to -40 °C, THF (1 mL). The reaction mixture was left to stir under nitrogen, at rt, for 1h to give a clear solution and a white precipitate.

Borane dimethylsulfide complex various amounts = (0.04 mL, 0.3686 mmol), (0.04 mL, 0.4866 mmol), (0.07 mL, 0.7372 mmol), (0.14 mL, 1.4744 mmol).

Representative procedure for the hydroboration of 1,3-cyclohexadiene in THF utilising the diene to borane addition mode: 1,3-cyclohexadiene (0.07 mL, 0.7372 mmol) was added dropwise to a solution of borane dimethylsulfide complex (various amounts) dissolved in pre-cooled, to -40 °C, THF (1 mL). The reaction mixture was left to stir under nitrogen, at rt, for 1h to give a clear solution and a white precipitate.

Borane dimethylsulfide complex various amounts = (0.04 mL, 0.3686 mmol), (0.04 mL, 0.4866 mmol), (0.07 mL, 0.7372 mmol), (0.14 mL, 1.4744 mmol).

Representative procedure for the hydroboration of 1,3-cyclohexadiene in diglyme utilising the borane to diene addition mode: Borane dimethylsulfide complex (various amounts) was added dropwise to a solution of 1,3-cyclohexadiene (0.07 mL, 0.7372 mmol) dissolved in pre-cooled, to -40 °C, diglyme (3.2 mL). The reaction mixture was left to stir under nitrogen, at rt, for 1h to give a clear solution and a white precipitate.

Borane dimethylsulfide complex various amounts = (0.04 mL, 0.3686 mmol), (0.04 mL, 0.4866 mmol), (0.07 mL, 0.7372 mmol), (0.14 mL, 1.4744 mmol).

White precipitate formed with 2 molar equivalent of borane dimethylsulfide complex; $\bar{\nu}_{max}$ cm^{-1} 2909 (C-H), 2842 (C-H), 2576 (B-H_t), 2502 (B-H_t), 1513 (B-H_b); ^{1}H ssNMR (700 MHz) +5.20 - +0.7 (xH, b); ^{13}C ssNMR (176 MHz) +36 - +15 (xC, br); ^{11}B ssNMR (224 MHz) +110 - -18 (xB, m).

Representative procedure for the hydroboration of 1,3-cyclohexadiene in diglyme utilising the diene to borane addition mode: 1,3-cyclohexadiene (0.07 mL, 0.7372 mmol) was added dropwise to a solution of borane dimethylsulfide complex (various amounts) dissolved in pre-cooled, to -40 °C, diglyme (3.2 mL). The reaction mixture was left to stir under nitrogen, at rt, for 1h to give a clear solution and a white precipitate.

Borane dimethylsulfide complex various amounts = (0.04 mL, 0.3686 mmol), (0.04 mL, 0.4866 mmol), (0.07 mL, 0.7372 mmol), (0.14 mL, 1.4744 mmol).

White precipitate formed with 2 molar equivalent of borane dimethylsulfide complex; $\bar{\nu}_{max}$ cm^{-1} 2908 (C-H), 2843 (C-H), 2575 (B-H_t), 2507 (B-H_t), 1511 (B-H_b); ^{1}H ssNMR (700 MHz) +5.20 - +0.7 (xH, b); ^{13}C ssNMR (176 MHz) +36 - +15 (xC, br); ^{11}B ssNMR (224 MHz) +110 - -18 (xB, m).

More diluted hydroboration of 1,3-cyclohexadiene in diglyme utilising the borane to diene addition mode: Borane dimethylsulfide complex (0.14 mL, 1.4744 mmol) was added dropwise to a solution of 1,3-cyclohexadiene (0.07 mL, 0.7372 mmol) dissolved in pre-cooled, to -40 °C, diglyme (16 mL). The reaction mixture was left to stir under nitrogen, at rt, for 1h to give a clear solution and a white precipitate.

Hydroboration of 1,3-cyclohexadiene in DCM utilising the borane to diene addition mode: Borane dimethylsulfide complex (0.14 mL, 1.4744 mmol) was added dropwise to a solution of 1,3-cyclohexadiene (0.07 mL, 0.7372 mmol) dissolved in DCM (3.2 mL). The reaction mixture was left to stir under nitrogen, at rt, for 1h to give a clear solution and traces of a white precipitate.

Hydroboration of 1,3-cyclohexadiene in diglyme utilising the borane to diene addition mode at -78 °C: Borane dimethylsulfide complex (0.14 mL, 1.4744 mmol) was added dropwise at -78 °C to a solution of 1,3-cyclohexadiene (0.07 mL, 0.7372 mmol) im THF (1 mL). The reaction mixture was left to stir under nitrogen for 1h to give a clear solution and a white precipitate.

Representative procedure for the hydroboration of 1,3-cyclohexadiene utilising slow diborane(6) gas release, in diglyme: 1,3-cyclohexadiene (0.09 mL, 0.9446 mmol) and sodium borohydride (0.0268g, 0.7085 mmol or 0.05360g, 1.4169 mmol or 1.0720g, 2.8338 mmol) were dissolved in pre-cooled, to -40 °C, diglyme (3.2 mL). Boron trifluoride diethyl ether complex (0.12 mL, 0.9446 mmol or 0.24 mL, 1.8892 mmol or 0.48 mL, 3.7784 mmol) was then added dropwise and the resulting reaction mixture was left to stir under nitrogen, at rt, for 1h to give a clear solution containing a white precipitate.

White precipitate formed from with 1 molar equivalent of diborane(6) gas; $\bar{\nu}_{max}$ cm^{-1} 2909 (C-H), 2843 (C-H), 2575 (B-H_t), 2507 (B-H_t), 1511 (B-H_b); ^{1}H ssNMR (700 MHz) +5.20 - +0.7 (xH, b); ^{13}C ssNMR (176 MHz) +36 - +15 (xC, br); ^{11}B ssNMR (224 MHz) +110 - -18 (xB, m).

Representative procedure for the hydroboration of 1,3-cyclohexadiene utilising fast diborane(6) gas release, in diglyme: 1,3-cyclohexadiene (0.09 mL, 0.9446 mmol) and boron trifluoride diethyl ether complex (0.12 mL, 0.9446 mmol or 0.24 mL, 1.8892 mmol or 0.48 mL, 3.7784 mmol) were dissolved in pre-cooled, to -40 °C, diglyme (3.2 mL). Sodium borohydride (0.0268g, 0.7085 mmol or 0.05360g, 1.4169 mmol or 1.0720g, 2.8338 mmol) was then added slowly and the resulting reaction mixture was left to stir under nitrogen, at rt, for 1h to give a clear solution containing a white precipitate.

White precipitate formed with 1 molar equivalent of diborane(6) gas; $\bar{\nu}_{max}$ cm^{-1} 2909 (C-H), 2843 (C-H), 2573 (B-H_t), 2506 (B-H_t), 1513 (B-H_b); ^{1}H ssNMR (700 MHz) +5.20 - +0.7 (xH, b); ^{13}C ssNMR (176 MHz) +36 - +15 (xC, br); ^{11}B ssNMR (224 MHz) +110 - -18 (xB, m).

Representative procedure for the hydroboration of 1,3-cyclohexadiene utilising slow diborane(6) gas release, in diglyme to yield deuterated precipitates: 1,3-cyclohexadiene (0.09 mL, 0.9446 mmol) and sodium deutereo borohydride (0.06145g, 1.4169 mmol) were dissolved in pre-cooled, to -40 °C, diglyme (3.2 mL). Boron tri-

fluoride diethyl ether complex (0.24 mL, 1.8892 mmol) was then added dropwise and the resulting reaction mixture was left to stir under nitrogen, at rt, for 1h to give a clear solution containing a deuterated white precipitate.

S.2.4 Control experiments

Boron trifluoride diethyl ether complex (0.12 mL, 0.9446 mmol or 0.24 mL, 1.8892 mmol or 0.48 mL, 3.7784 mmol) was added dropwise to a solution of 1,3-cyclohexadiene (0.09 mL, 0.9446 mmol) in pre-cooled, to -40 °C, diglyme (3.2 mL). The resulting reaction mixture was left to stir under nitrogen, at rt, for 1h to give a coloured solution (pink-orange to blue-green and eventually to black).

Boron trifluoride diethyl ether complex (0.05 mL, 0.3813 mmol) was added dropwise to a solution of phenylbis(4-vinylphenyl)phosphine (0.1098g, 0.3813 mmol) in diethyl ether (5 mL). The resulting reaction mixture was left to stir under nitrogen, at rt, for 1h to give a clear solution.

Hydroboration of 1,3-cyclohexadiene using the published procedure:[3] Borane dimethylsulfide (0.32 mL, 3.33 mmol) was added dropwise to a solution of 1,3-cyclohexadiene (0.95 mL, 10 mmol) in THF (10 mL) at 0 °C and was left to stir at this temperature for 1h to give a clear solution.

S.2.5 Solubility study of the precipitates formed during hydroboration of 1,3-cyclohexadiene

Solvent (2 mL) was added to a solid (0.050g) and the mixture was left at rt for one week with occasional shaking.

Solvent = THF, DCM, acetonitrile, diglyme, diethyl ether, 1,3-dioxane, 1,4-dioxane, glyme, diglyme, dimethylsulfide, acetic acid, acetone, benzene, DMF, chloroform, cyclohexane, DMSO, hexane, methanol.

Solid = precipitate formed during the reaction of 1,3-cyclohexadiene and: 0.5 equivalent of borane dimethylsulfide in diglyme utilising borane to diene addition, 1 equivalent of borane dimethylsulfide in diglyme utilising borane to diene addition, 2 equivalent of borane dimethylsulfide in diglyme utilising borane to diene addition, 0.5 equivalent of borane dimethylsulfide in diglyme utilising diene to borane addition, 1 equivalent of borane dimethylsulfide in diglyme utilising diene to borane addition, 2 equivalent of borane dimethylsulfide in diglyme utilising diene to borane addition, 0.5 equivalent of diborane(6) gas in diglyme utilising the slow release method, 1 equivalent of diborane(6) gas in diglyme utilising the slow release method, 2 equivalent of diborane(6) gas in diglyme utilising the slow release method, 0.5 equivalent of diborane(6) gas in diglyme utilising the fast release method, 1 equivalent of diborane(6) gas in diglyme utilising the fast release method, 2 equivalent of diborane(6) gas in diglyme utilising the fast release method.

S.2.6 Basic oxidation of the precipitates formed during the hydroboration of various dienes

Representative procedure for the oxidation of the precipitates formed during the hydroboration of specific dienes: A solid (various) (0.0500g, unknown mmol) was placed in THF (5 mL) at 0 °C. Sodium hydroxide (2 mL, 3M) was added followed by the dropwise addition of hydrogen peroxide (2 mL, 30% in H_2O) and the reaction mixture was left to stir at rt for 3h. After the oxidation was complete, the reaction was cooled to 0 °C and quenched with sodium hydroxide (2 mL, 1M) and a saturated solution of sodium thiosulfate (2 mL). The organic layer was extracted with CH_2Cl_2 (3 $\times$ 5 mL) and evaporated under reduced pressure to give a mixture of alcohols and diols.

Solid = all precipitates formed during all hydroborations of dienes which yielded such materials.

S.2.7 Synthesis and reactions of the model compounds dicyclohexylborane 9 and tricyclohexylborane 8

Synthesis of dicyclohexylborane **9**: Borane dimethylsulfide complex (0.77 mL, 8.1197 mmol) was added instantly, in one portion, to a solution of cyclohexene (2.47 mL, 0.02438 mol) dissolved in diethyl ether (5 mL). The reaction mixture was left to stir under nitrogen, at rt, for 1h to give a clear solution containing a white precipitate. The white precipitate was filtered under nitrogen and washed with cold diethyl ether (3 × 5 mL) to give dicyclohexylborane **9** (1.1601g, 3.2552 mmol, 80%) as a white powder; $\bar{\nu}_{max}$ cm^{-1} 2915 (C-H; BC_6H_{11}), 2843 (C-H; BC_6H_{11}), 1521 (B-H_b; $(R_2BH)_2$); ^1H NMR (500 MHz; $CDCl_3$); 1.75-1.70 (16H, m, 16 × CH_2; BC_6H_{11}), 1.53-1.48 (4H, m, 4 × CH; BC_6H_{11}), 1.42-1.35 (8H, 8 × CH_2; BC_6H_{11}), 1.35-1.24 (16H, 16 × CH_2; BC_6H_{11}); ^1H NMR (700 MHz) +2.11 - +0.80 (33H, br, 3 × CH, 15 × CH_2, 2 × B-H; $(R_2BH)_2$); ^{13}C NMR (125 MHz; $CDCl_3$) 29.88 (8C, 8 × CH_2; BC_6H_{11}), 27.98 (8C, 8 × CH_2; BC_6H_{11}), 27.92 (4C, 4 × CH_2; BC_6H_{11}), 26.65 (4C, 4 × CH_2; BC_6H_{11}; ^{13}C ssNMR (176 MHz) +33.50 - +31.00 (4C, br, 4 × CH; BC_6H_{11}), +31.00 - +21.10 (20C, m, 20 × CH_2; BC_6H_{11}); ^{11}B NMR (160 MHz; $CDCl_3$) +29.64 (2B, br, B; $(R_2BH)_2$); ^{11}B ssNMR (224 MHz) +22.50 (2B, br, B; $(R_2BH)_2$).

Synthesis of tricyclohexylborane **8**: Cyclohexene (0.3 mL, 2.8060 mmol) was added dropwise to a solution of dicyclohexylborane **9** (0.5000g, 1.4030 mmol) in diethyl ether (20 mL). The reaction mixture was left to stir for 1h at rt. The solution was then cooled slowly to -40 °C to yield needle-like crystals which were filtered under nitrogen and washed with cold diethyl ether (3 × 5 mL) to give tricyclohexylborane **8** (0.3469g, 1.3328 mmol, 95%) as a white needle-like solid; $\bar{\nu}_{max}$ cm^{-1} 2913 (C-H; BC_6H_{11}), 2844 (C-H; BC_6H_{11}); ^1H NMR (500 MHz; $CDCl_3$) 1.73-1.71 (9H, m, 3 × CH_2 and 3 × CH; BC_6H_{11}), 1.53-1.47 (3H, m, 3 × CH; BC_6H_{11}), 1.47-1.38 (6H, m, 3 × CH_2; BC_6H_{11}), 1.28-1.16 (15H, m, 6 × CH_2 and 3 × CH; BC_6H_{11}); ^1H NMR (700 MHz) +2.20 - +0.70 (33H, br, 3 × CH, 15 × CH_2; R_3B); ^{13}C NMR (125 MHz; $CDCl_3$) 34.98 (3C, br, 3 × CH; BC_6H_{11}), 27.55 (6C, s, 6 × CH_2; BC_6H_{11}), 27.28 (3C, s, 3 × CH_2; BC_6H_{11}), 26.47 (6C, s, 6 × CH_2; BC_6H_{11}); ^{13}C ssNMR (176 MHz) +36.01 - 33.22 (3C, br, 3 × CH; BC_6H_{11}), +31.14 - +25.00 (15C, m, 15 × CH_2; BC_6H_{11}); ^{11}B NMR (160 MHz; $CDCl_3$) +81.16 (1B, br, B; R_3B); ^{11}B ssNMR (224 MHz) +69.40 (1B, s, B; R_3B).

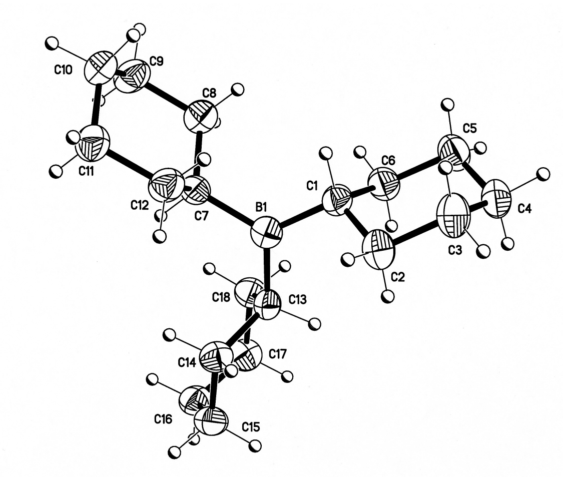

Figure S1: The molecular structure of tricyclohexylborane **8**.

Molecular structure distances (Å) and angles (deg): B1-C1 1.575(3), B1-C7 1.578(3), B1-C13 1.582(3), C1-C2 1.535(3), C1-C6 1.537(3), C1-H1A 1.0000, C2-C3 1.526(3), C2-H2A 0.9900, C2-H2B 0.9900, C3-C4 1.516(3), C3-H3A 0.9900, C3-H3B 0.9900, C4-C5 1.512(3), C4-H4A 0.9900, C4-H4B 0.9900, C5-C6 1.521(3), C5-H5A 0.9900, C5-H5B 0.9900, C6-H6A 0.9900, C6-H6B 0.9900, C7-C8 1.531(3), C7-C12 1.534 (3), C7-H7A 1.0000, C8-C9 1.527(3), C8-H8A 0.9900, C8-H8B 0.9900, C9-C10 1.510(3), C9-H9A 0.9900, C9-H9B 0.9900, C10-C11 1.513(3), C10-H10A 0.9900, C10-H10B 0.9900, C11-C12 1.522(3), C11-H11A 0.9900, C11-H11B 0.9900, C12-H12A 0.9900, C12-H12B 0.9900, C13-C14 1.534(3), C13-C18 1.542(3), C13-H13A 1.0000, C14-C15 1.525(3), C14-H14A 0.9900, C14-H14B 0.9900, C15-C16 1.514(3), C15-H15A 0.9900, C15-H15B 0.9900, C16-C17 1.510(3), C16-H16A 0.9900, C16-H16B 0.9900, C17-C18 1.527(3), C17-H17A 0.9900, C17-H17B 0.9900, C18-H18A 0.9900, C18-H18B 0.9900, C1-B1-C7 119.95(18), C1-B1-C13 120.34(19), C7-B1-C13 119.71(19), C2-C1-C6 109.42(17), C2-C1-B1 113.69(18), C6-C1-B1 109.78(17), C2-C1-H1A 107.9, C6-C1-H1A 107.9, B1-C1-H1A 107.9, C3-C2-C1 111.76(19), C3-C2-H2A 109.3, C1-C2-H2A 109.3, C3-C2-H2B 109.3, C1-C2-H2B 109.3, H2A-C2-H2B 107.9, C4-C3-C2 110.95(18), C4-C3-H3A 109.4, C2-C3-H3A 109.4, C4-C3-H3B 109.4, C2-C3-H3B 109.4, H3A-C3-H3B 108.0, C5-C4-C3 111.49(19), C5-C4-H4A 109.3, C3-C4-H4A 109.3, C5-C4-H4B 109.3, C3-C4-H4B 109.3, H4A-C4-H4B 108.0, C4-C5-C6 111.66(19), C4-C5-H5A 109.3, C6-C5-H5A 109.3, C4-C5-H5B 109.3, C6-C5-H5B 109.3, H5A-C5-H5B 107.9, C5-C6-C1 112.48(18), C5 C6 H6A 109.1, C1-C6-H6A 109.1, C5-C6-H6B 109.1, C1-C6-H6B 109.1, H6A-C6-6B 107.8, C8-C7-C12 109.02(16), C8-C7-B1 113.98(17), C12-C7-B1 109.62(17), C8-C7-H7A 108.0, C12-C7-H7A 108.0, B1-C7-H7A 108.0, C9-C8-C7 112.24(18), C9-C8-H8A 109.2, C7-C8-H8A 109.2, C9-C8-H8B 109.2, C7-C8-H8B 109.2, H8A-C8-H8B 107.9, C10-C9-C8 111.43(18), C10-C9-H9A 109.3, C8-C9-H9A 109.3, C10-C9-H9B 109.3, C8-C9-H9B 109.3, H9A-C9-H9B 108.0, C9-C10-C11 111.74(18), C9-C10-H10A 109.3, C11-C10-H10A 109.3, C9-C10-H10B 109.3, C11-C10-H10B 109.3, H10A-C10-H10B 107.9, C10-C11-C12 111.05(19), C10-C11-H11A 109.4, C12-C11-H11A 109.4, C10-C11-H11B 109.4, C12-C11-H11B 109.4, H11A-C11-H11B 108.0, C11-C12-C7 112.32(18), C11-C12-H12A 109.1, C7-C12-H12A 109.1, C11-C12-H12B 109.1, C7-C12-H12B 109.1, H12A-C12-H12B 107.9, C14-C13-C18 110.26(17), C14-C13-B1 111.75(17), C18-C13-B1 109.56(17), C14-C13-H13A 108.4, C18-C13-H13A 108.4, B1-C13-H13A 108.4, C15-C14-C13 113.03(18), C15-C14-H14A 109.0, C13-C14-H14A 109.0, C15-C14-H14B 109.0, C13-C14-H14B 109.0, H14A-C14-H14B 107.8, C16-C15-C14 110.59(18), C16-C15-H15A 109.5, C14-C15-H15A 109.5, C16-C15-H15B 109.5, C14-C15-H15B 109.5, H15A-C15-H15B 108.1, C17-C16-C15 111.26(18), C17-C16-H16A 109.4, C15-C16-H16A 109.4, C17-C16-H16B 109.4, C15-C16-H16B 109.4, H16A-C16-H16B 108.0, C16-C17-C18 110.43(18), C16-C17-H17A 109.6, C18-C17-H17A 109.6, C16-C17-H17B 109.6, C18-C17-H17B 109.6, H17A-C17-H17B 108.1, C17-C18-C13 112.62(18), C17-C18-H18A 109.1, C13-C18-H18A 109.1, C17-C18-H18B 109.1, C13-C18-H18B 109.1, H18A-C18-H18B 107.8.

Reaction of dicyclohexylborane **9** with borane dimethylsulfide: Borane dimethylsufide (0.07 mL, 0.7372 mmol) was added dropwise to a solution of dicyclohexylborane **9** (0.1314g, 0.3686 mmol) in diethyl ether (5 mL). The reaction mixture was left to stir for 1h at rt to give a clear solution.

Reaction of tricyclohexylborane **8** with borane dimethylsulfide: Borane dimethylsufide (0.07 mL, 0.7372 mmol) was added dropwise to a solution of tricyclohexylborane **8** (0.09591g, 0.3686 mmol) in diethyl ether (5 mL). The reaction mixture was left to stir for 1h at rt to give a clear solution.

Reaction of tricyclohexylborane **8** with dimethylsulfide: Dimethylsulfide (0.16 mL) was added dropwise to a solution of tricyclohexylborane **8** (0.1098g, 0.42187 mmol) in diethyl ether (5 mL). The reaction mixture was left to stir for 1h at rt to give a clear solution.

S.3 Purity of commercially available borane solutions

During our studies we analysed various different commercially available $BH_3 \cdot THF$ and $BH_3 \cdot SMe_2$ solutions of which most were found to contain high amounts of impurities. These are most commonly hydrolysis products and/or products from borane-THF ring opening side reactions. We observed trace amounts of impurities present even in the purest commercially available solutions at -10 and -29 ppm in the ^{11}B NMR spectrum, which were not found to participate in the hydroboration reaction as they were consistently isolated in the filtrate after dihydroboration was complete and the same hydroboration materials were obtained in their absence.

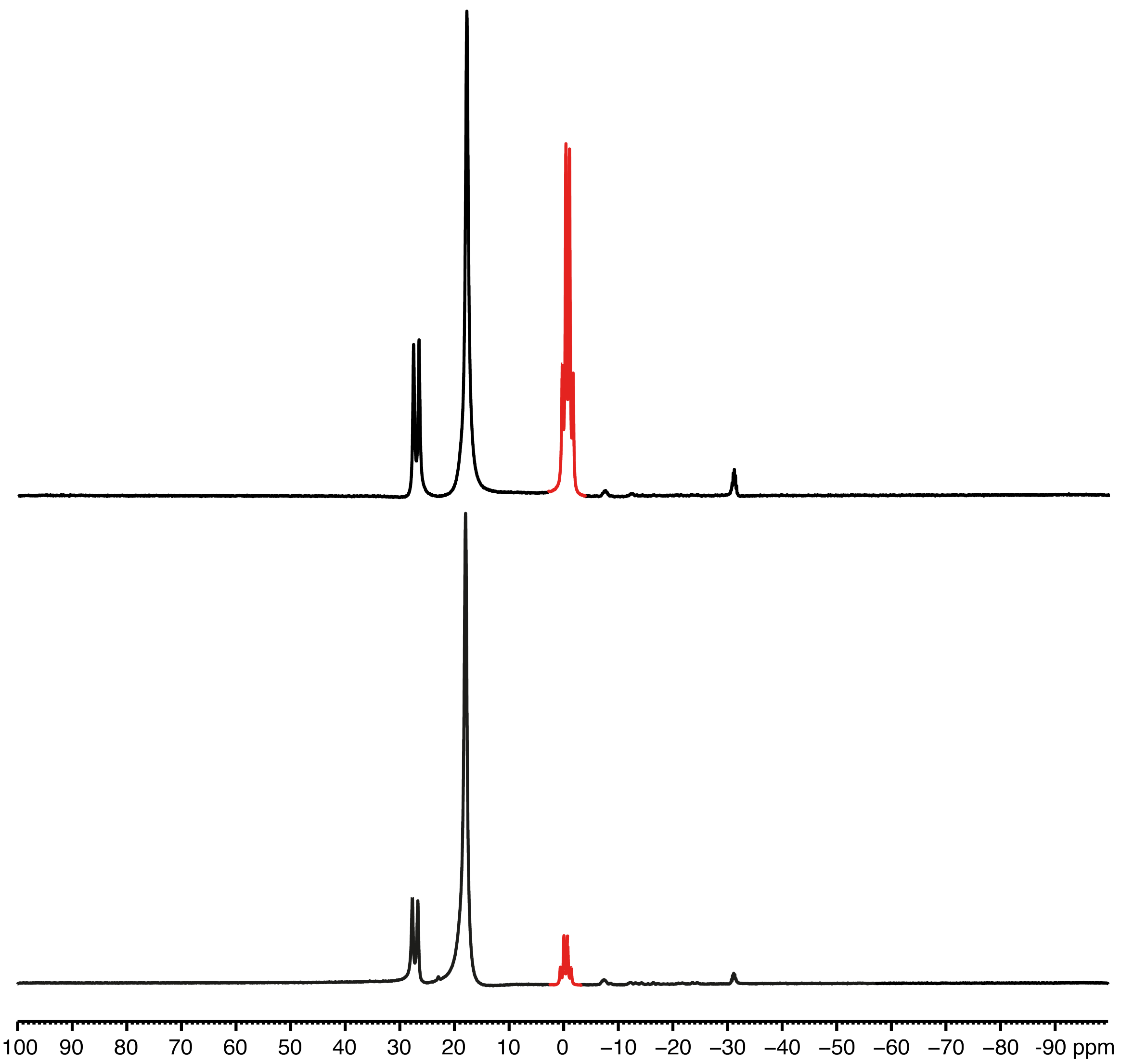

Figure S2: The ^{11}B NMR spectra of two commercial $BH_3 \cdot THF$ solutions, where the $BH_3 \cdot THF$ species is labelled in red.

S.4 Synthesis of borane solutions

Alternatively, borane solutions can be freshly prepared. We found that the reaction between $NaBH_4$ and $BF_3{\cdot}OEt_2$ yielded much purer solutions in comparison to the more commonly used reaction between $NaBH_4$ and I_2. We devised a simple setup for the synthesis of such solutions in a glovebox shown in Figure S3.

Although simple, repeating the reaction using this setup yielded the desired $BH_3{\cdot}THF$ and $BH_3{\cdot}SMe_2$ in high purity ($\sim$ 99% estimated by ^{11}B and ^{1}H NMR shown in Figure S4). It was vital to include an empty second vial between the reaction and isolation vials, as diborane(6) gas was found to carry solvents (such as ether and diglyme) causing contamination of the final borane solutions, a problem which was also observed by G.F. Freeguard and L.H. Long.[4] Despite a literature report by J.V.B. Kanth and H.C. Brown describing an improved procedure of this reaction, by utilising other ether solvents such as triglyme and tetraglyme,[5] this reaction was found to be highly successful while diglyme was used in this three vial setup.

The molarity of these solutions was determined through hydrolysis by measuring hydrogen release and by triphenylphosphine complexation as this reaction proceeds quantitatively.

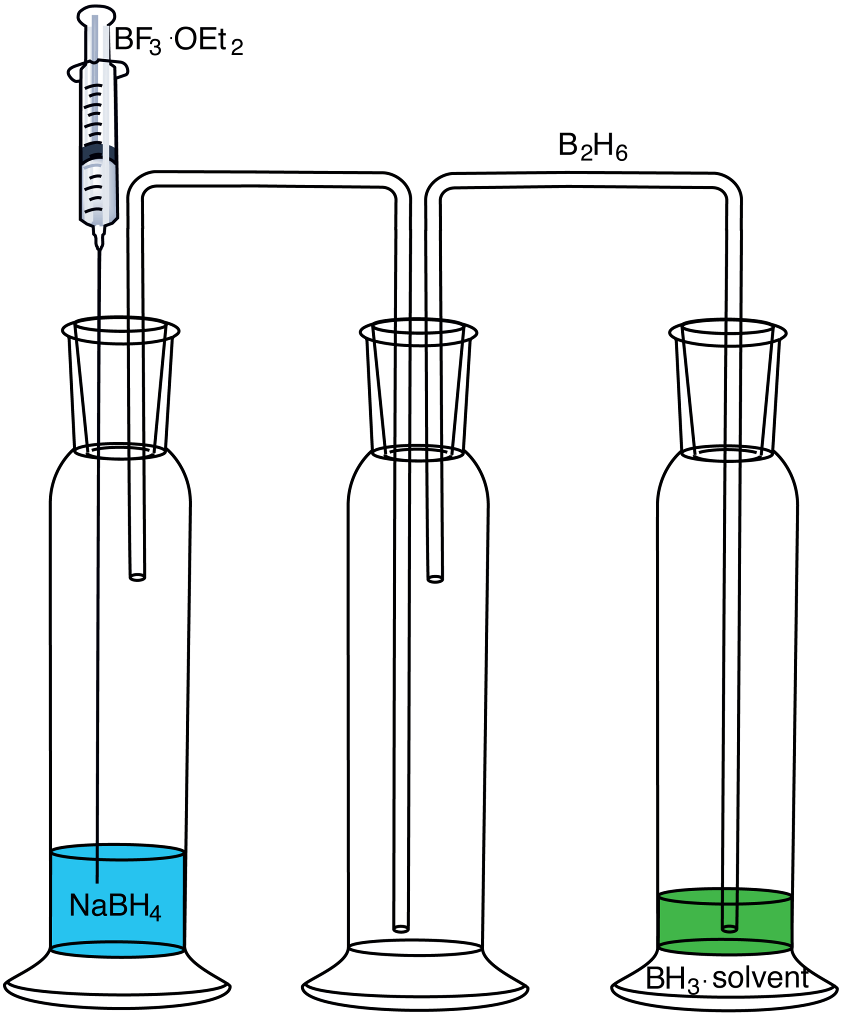

Figure S3: The three vial setup for the synthesis of B_2H_6 using $NaBH_4$ and $BF_3{\cdot}OEt_2$.

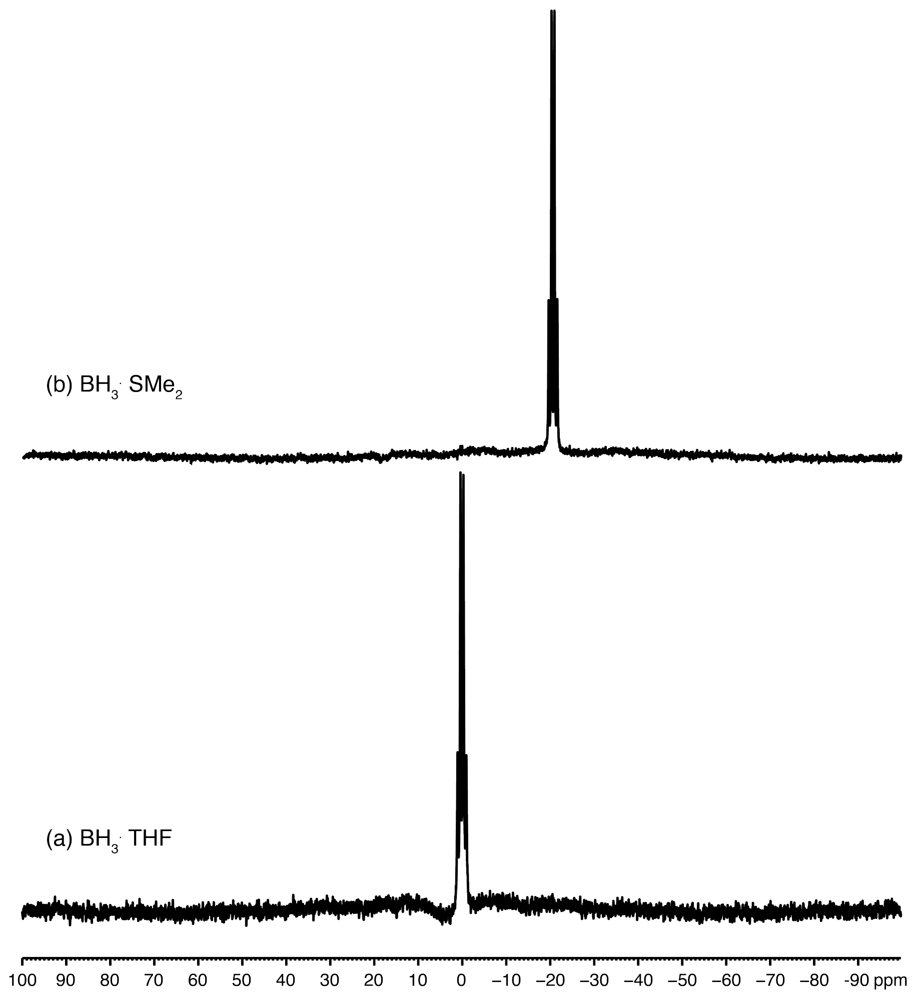

Figure S4: The ^{11}B NMR spectra of (a) BH_3·THF and (b) BH_3·SMe_2 synthesized by utilising the three vial setup while employing the $NaBH_4$ and BF_3·OEt_2 reaction.

S.5 Hydroboration of 1,3-cyclohexadiene following the literature methods

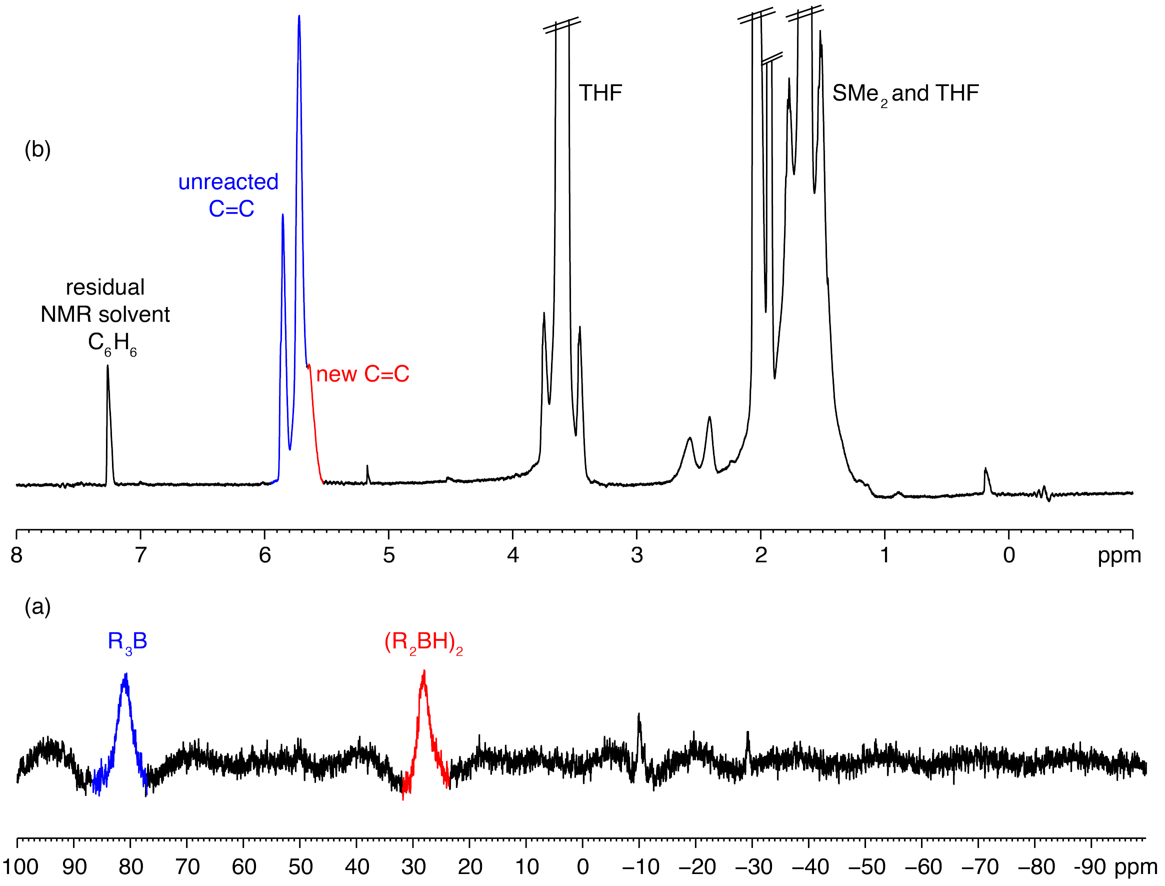

Figure S5: The (a) ^{11}B NMR and (b) ^{1}H NMR spectrum of the hydroboration of 1,3-cyclohexadiene **1** with borane $BH_3{\cdot}SMe_2$ using the published by H.C. Brown and K. Bhat,[3] hydroboration conditions.

Analysis of the oxidised hydroboration mixture by GC-MS revealed the formation of 2-cyclohexene-1-ol **2** and 3-cyclohexene-1-ol **3** in a 60:40 ratio, in agreement with the published report.[3] Interstingly, the reaction mixture also contained a considerable amount of hydrocarbon rearrangement products (evident by GC-MS) which were not observed when our conditions were utilised.

S.6 Hydroboration of 1,3-cyclohexadiene

Although the reactions presented in the main paper were performed in diglyme and at different concentrations (which allowed direct comparison to the diborane(6) reactions) the hydroboration species observed when THF was used were similar with the only difference being the physical properties of the materials formed (diglyme = powder, THF= glassy). As in the case of diglyme, all reactions irrespective of borane equivalents or mode of reagent addition, yielded an insoluble material.

Similarly to the case of diglyme, the same trends were observed.

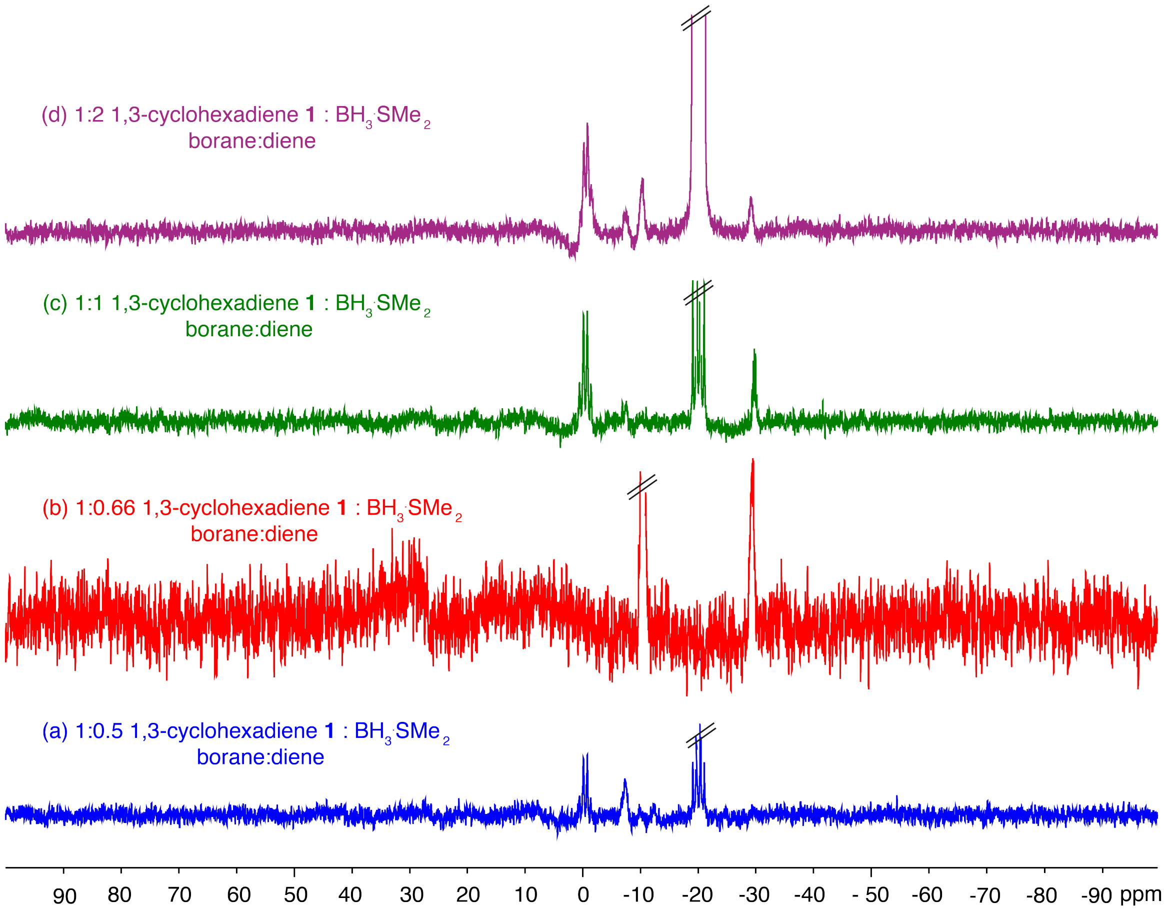

Figure S6: The ^{11}B NMR spectra of the filtrates from the hydroboration of 1,3-cyclohexadiene **1** using different equivalents of borane $BH_3{\cdot}SMe_2$ while adding borane to diene in diglyme.

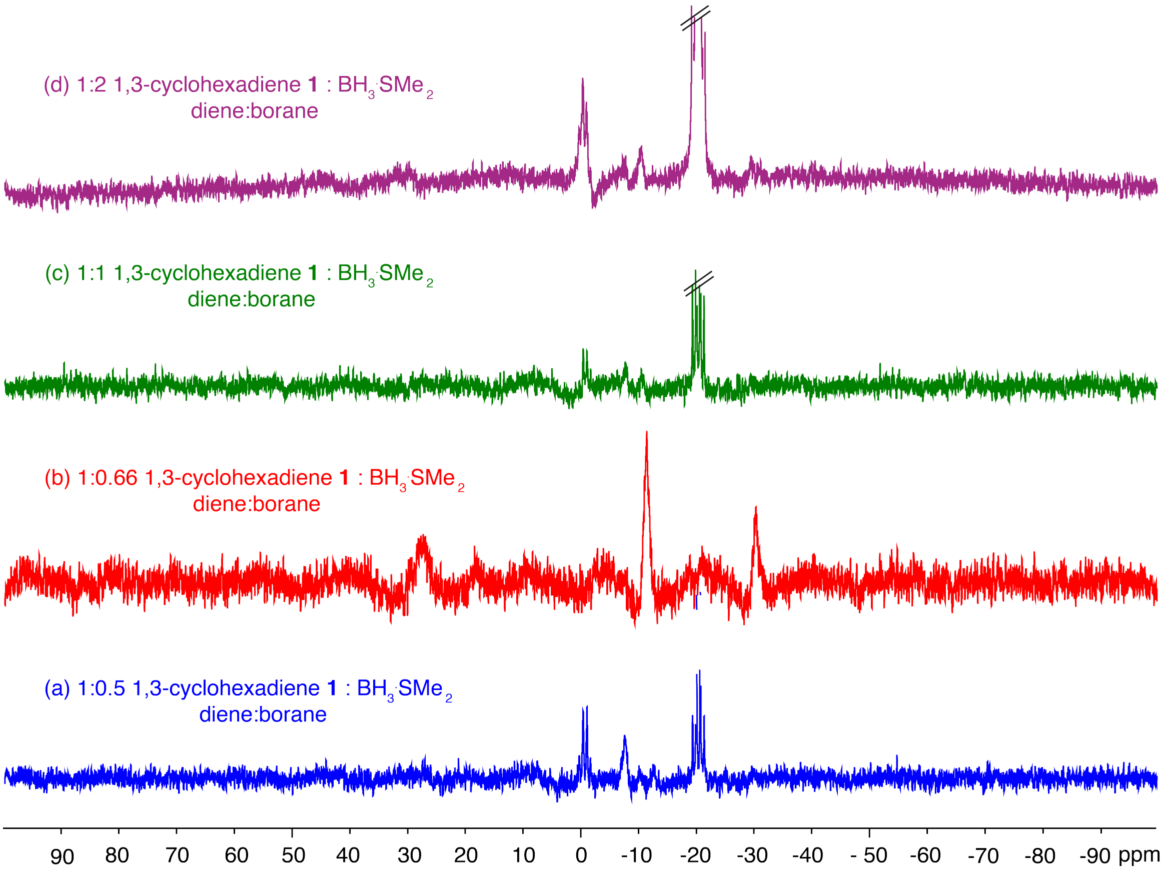

Figure S7: The ^{11}B NMR spectra of the filtrates from the hydroboration of 1,3-cyclohexadiene **1** using different equivalents of borane $BH_3{\cdot}SMe_2$ while adding diene to borane in diglyme.

S.6.1 Control experiments for diborane(6)

On addition of $BF_3 \cdot OEt_2$ to 1,3-cyclohexadiene **1** in diglyme, a pink-orange color was observed which turned darker over time to blue-green and eventually black. These colour changes are not observed when the reaction is performed in the presence of $NaBH_4$ and therefore, the possibility that this process is due to cationic polymerisation can be eliminated (also these materials are very similar, by solid state NMR and FT-IR to the ones formed under the same conditions when borane $BH_3 \cdot SMe$ is used). Analysis of the black solution by ^{11}B NMR showed the presence of only the $BF_3 \cdot OEt_2$ used (0.00 ppm). Similarly, there were no new peaks observed in the ^{1}H NMR spectrum. The observed colour change must rise from an interaction of the C=C bonds with BF_3.

S.7 Analysis and characterisation of the materials

S.7.1 Solubility of the materials and products formed

The solutions obtained from the reactions of these materials with acetonitrile, dimethyl sulfide and triphenylphosphine, were analysed by NMR and found to contain pure $BH_3 \cdot$Lewis base complexes in all three cases. These experiments were also performed while using materials which were synthesised with deuterated diborane(6) gas. The incorporation of C-D and B-D bonds was confirmed by lower frequency stretches in the FT-IR, resulting in vibrations to the right of the C-H or B-H ones, due to the heavier deuterium atom. Similarly to before, addition of a solution of triphenylphosphine in THF, resulted in elimination of a borane species that contained atleast one B-D bond. A borane species was observed possessing a slightly upfield shifted signal in the ^{11}B NMR spectrum (-39 ppm), but identical in the ^{31}P spectrum, to the non deuterated complex, $PPh_3.BH_3$ (+21.37 ppm), however, appearing as a broad signal with no multiplicity. This could be due to low resolution or the presence of both B-H and B-D bonds.

Analysis of the solution by ^{2}D NMR showed only the presence of the deuterated solvent used, possibly due to fast hydrogen-deuterium exchange. As a result, the source of borane is most likely due to the materials containing a R_2BHBH_3 species, of which symmetrical cleavage by triphenylphosphine would be excepted to yield such complex with the remaining R_2BH still attached on the polymer network. This would also explain the presence of insoluble material even after the complexation reaction has taken place. Alternatively, retro-hydroboration could be considered, however, this is most probably not the case, as it would be expected to result complete dissolution of the material's network (unless this elimination is selective) to form soluble species with no precipitate left over. Similar observations were made when acetonitrile and dimethyl sulfide were used.

S.7.2 FT-IR

Unfortunately, traditional methods for the preparation and manipulation of air sensitive samples for IR were found to be inadequate for boron hydride species as the inert oils commonly used give rise to bands present in the B-H regions (both terminal and bridging depending on oil used). As a result, the FT-IRs of all solids mentioned in this manuscript were obtained from measurements of the neat solids by an instrument placed inside a nitrogen filled glove box. In this way, all B-H bonds were easily identified and distinguished, as terminal B-H_t bonds which are characteristically different than the bridged B-H_b, with resonances centred around +2400 cm^{-1} and +1500 cm^{-1} respectively.

S.7.2.1 FTIR of model compounds

Despite several attempts to recrystallise **9** including co-crystallisation with the more crystalline tricyclohexylborane **8** R_3B, a crystal suitable for x-ray crystallography was not obtained. The identity of dicyclohexylborane **9** $(R_2BH)_2$ was further confirmed by addition of 2 equivalents of cyclohexene **7** to the isolated species in hand, clearly forming the further substituted or hydroborated tricyclohexylborane **8** R_3B as confirmed by NMR. FT-IR analysis of tricyclohexylborane **8** R_3B under anaerobic conditions, proved the absence of any B-H bonds.

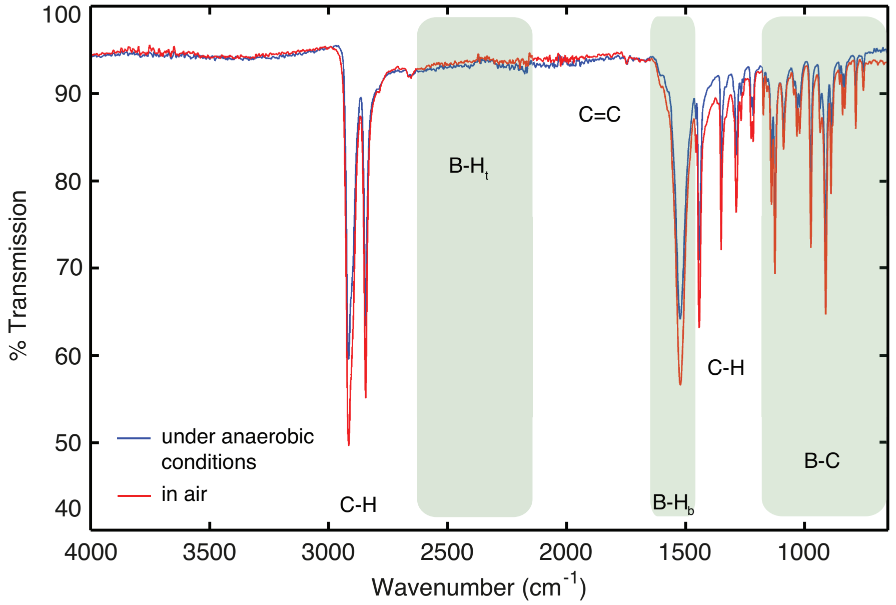

Figure S8: The FT-IR of dicyclohexylborane **9** $(R_2BH)_2$ (a) under aerobic conditions (blue) and (b) in air (red).

Recrystallisation of this compound in cold diethyl ether, provided needle-like crystals suitable for x-ray crystallography with B-C bonds of 1.58Å. (for bond lengths and angles see experimental).

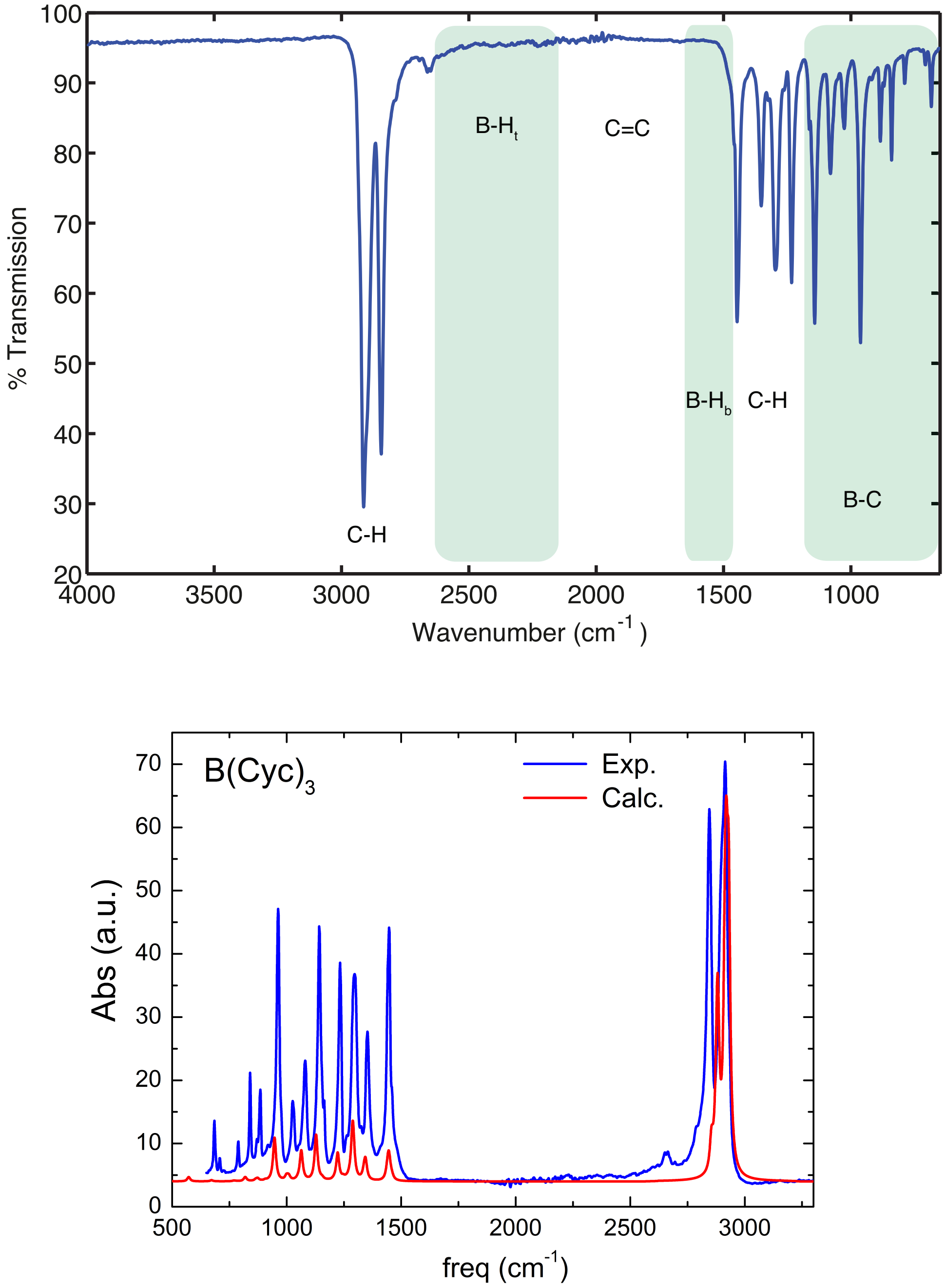

Figure S9: Top panel: The FT-IR of tricyclohexylborane **8** R_3B under aerobic conditions. Bottom panel: Comparison of the experimental and calculated IR spectra of tricyclohexylborane. The IR calculations were carried out at the B3LYP/6-311++G(d,p) level of theory on structures optimized at using B3LYP/6-31G(d). The frequencies were scaled using the recommended 0.967 factor (NIST Computational Chemistry Comparison and Benchmark Database, Release 16a, August 2013).

S.7.2.2 FTIR of materials

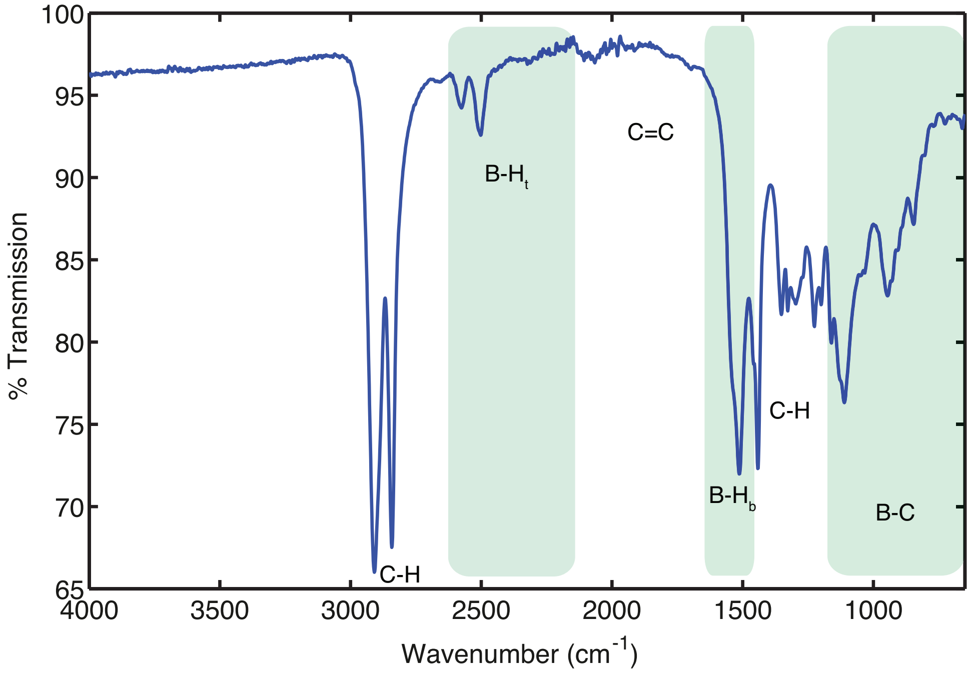

Figure S10: The FT-IR of the solid obtained from the reaction between 1:2 molar equivalents of 1,3-cyclohexadiene **1** added to borane $BH_3{\cdot}SMe_2$.

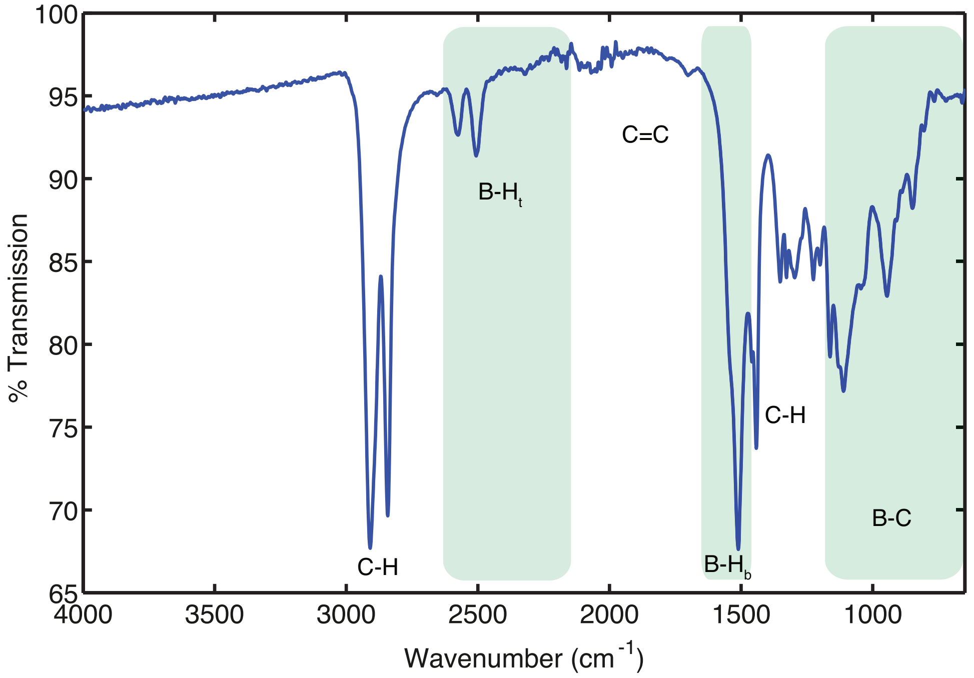

Figure S11: The FT-IR of the solid obtained from the reaction between 1:1 molar equivalents of 1,3-cyclohexadiene **1** and diborane(6) B_2H_6 utilising slow gas release.

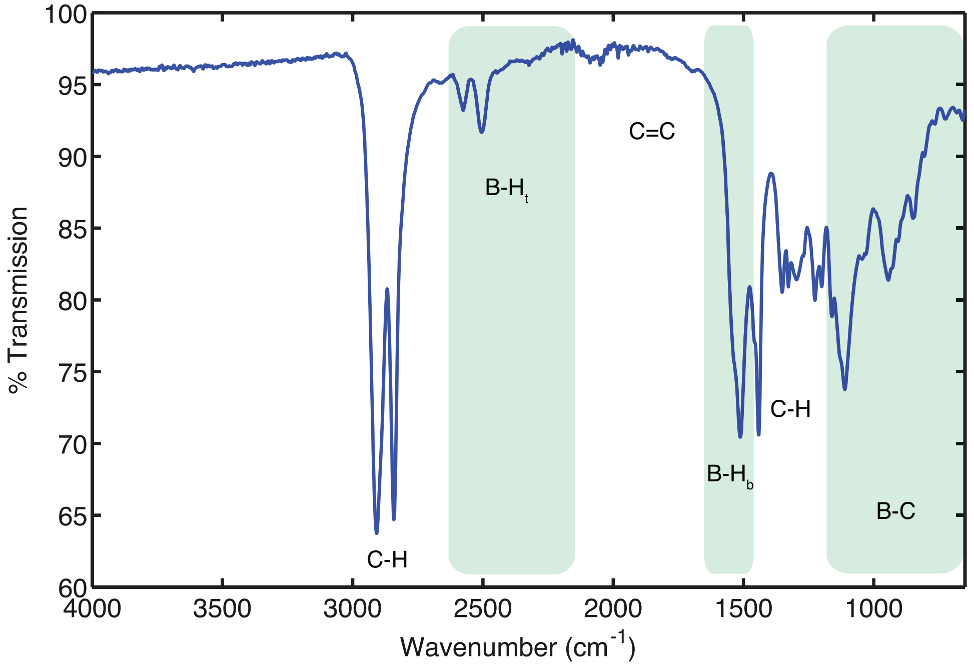

Figure S12: The FT-IR of the solid obtained from the reaction between 2:1 molar equivalents of borane $BH_3{\cdot}SMe_2$ added to 1,3-cyclohexadiene **1**.

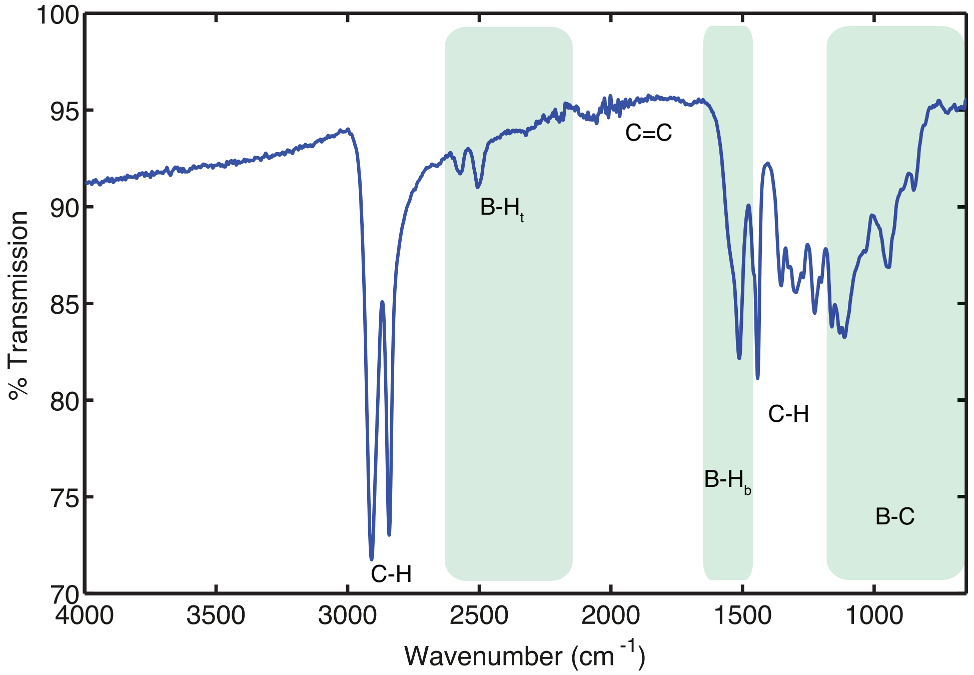

Figure S13: The FT-IR of the solid obtained from the reaction between 1:1 molar equivalents of 1,3-cyclohexadiene **1** and diborane(6) B_2H_6 utilising fast gas release.

S.7.2.3 Hydroboration of cyclohexene

An equivalent and mode of addition study was performed on cyclohexene **7** (^{11}B NMR spectra shown in Figure S14) in order to understand the species formed in this simplified reaction and identify which of these species are possibly present in the insoluble solids. These experiments also allowed for the determination of the best conditions for the synthesis and isolation of the model $(R_2BH)_2$ **9** and R_3B **8** species.

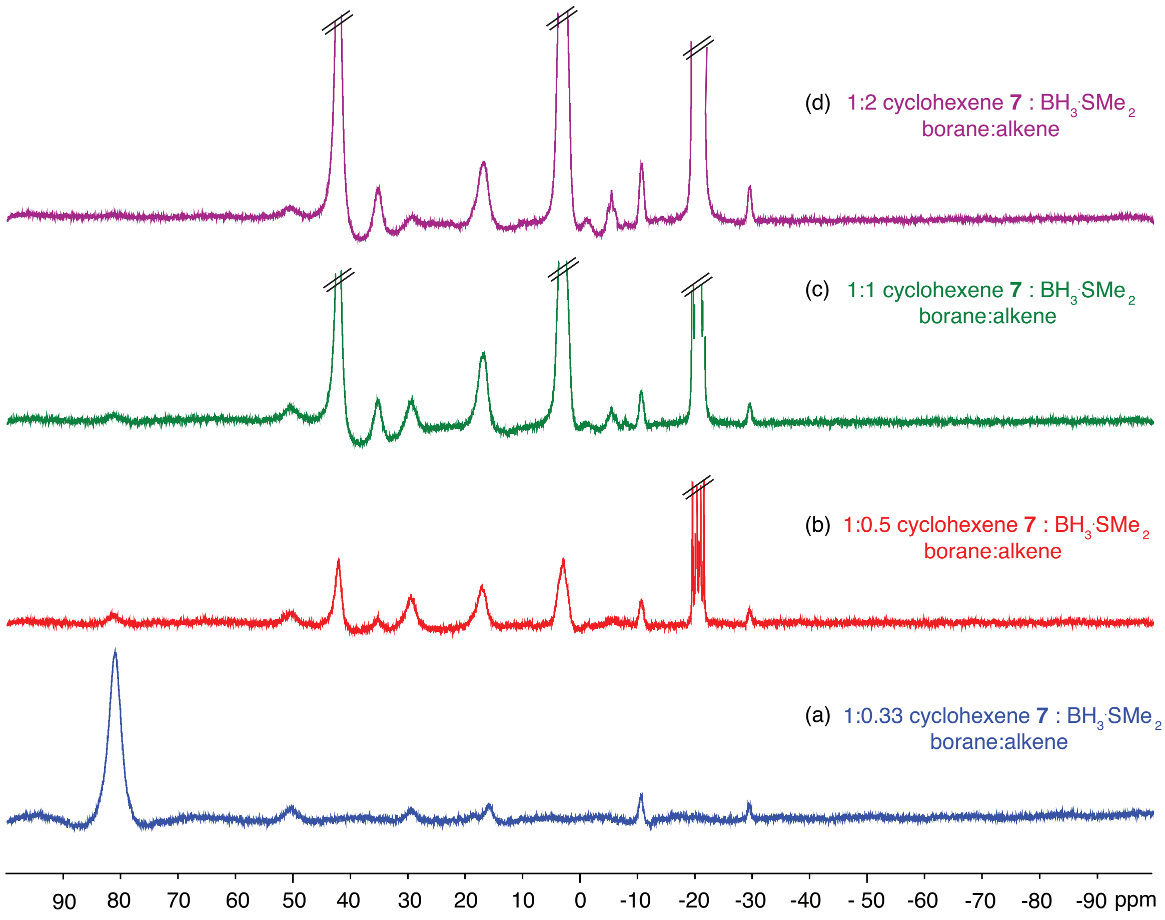

Figure S14: The ^{11}B NMR spectra of the boron to alkene hydroborations of cyclohexene **7** with (a) 0.33 (b) 0.5 (c) 1 and (d) 2 equivalents of borane $BH_3{\cdot}SMe_2$ in THF.

When 0.33 equivalents of the borane were added to the alkene, tricyclohexylborane **8** R_3B was formed, almost exclusively at +81 ppm as observed by ^{11}B NMR. Small amounts of an unknown species at +50 ppm were also observed together with traces of the dicyclohexylborane **9** $(R_2BH)_2$ at +29 ppm and an unknown species at +16 ppm. Addition of 0.5, 1 and 2 equivalents of borane $BH_3{\cdot}SMe_2$ leaded to the formation of almost identical boron species including R_2BHBH_3 (+42 and +3 ppm), R_2BHBH_2R (+35 and +17 ppm) and $(R_2BH)_2$ (+29 ppm) with the major difference being the intensity of the main product, R_2BHBH_3. The amount of this species formed was found to be directly proportional to the amount of unreacted borane present in solution. Small amounts of $BH_3{\cdot}THF$ were also observed (-0.90 ppm) as well as $RBH_2{\cdot}SMe_2$ appearing as an unresolved triplet (-5 ppm). The absence of tricyclohexylborane **8** R_3B, when higher equivalents of borane are used, is probably due to further reactions of this borane with unreacted borane $BH_3{\cdot}SMe_2$. The presence of the latter indicates, as before, that higher levels of hydroboration have occurred leading to products beyond monohydroboration. The results were almost identical when the opposite mode of reagent addition (alkene to borane) was explored (see Figure S15), suggesting again that the effect of the mode of reagent addition is negligible when drop wise addition is employed. The same species were observed when cyclohexene **7** was hydroborated with various equivalents of diborane(6) gas, released *in situ*.

It is worth noting that although the $(RBH_2)_2$ species have been found to be highly reactive by undergoing further hydroboration, it was observed that they can be obtained over time *via* disproportionation or scrambling reactions.

Dihydroboration of cyclohexene **7** with diborane(6) gas in diglyme yielded selectively the corresponding R_2BHBH_3 species (peak at -1 ppm due $NaBF_4$, by-product from the *in situ* diborane(6) gas release) as seen in Figure S16 part (a). Incredibly, after 1 month, the solution contained a range of different species including R_2BHBH_3, R_2BH_2R, $(R_2BH)_2$ and $(RBH_2)_2$ as the main product. Further monitoring of the solution (up to 2 months), showed no observable change indicating that the $(RBH_2)_2$ species is stable in the absence of C=C bonds.

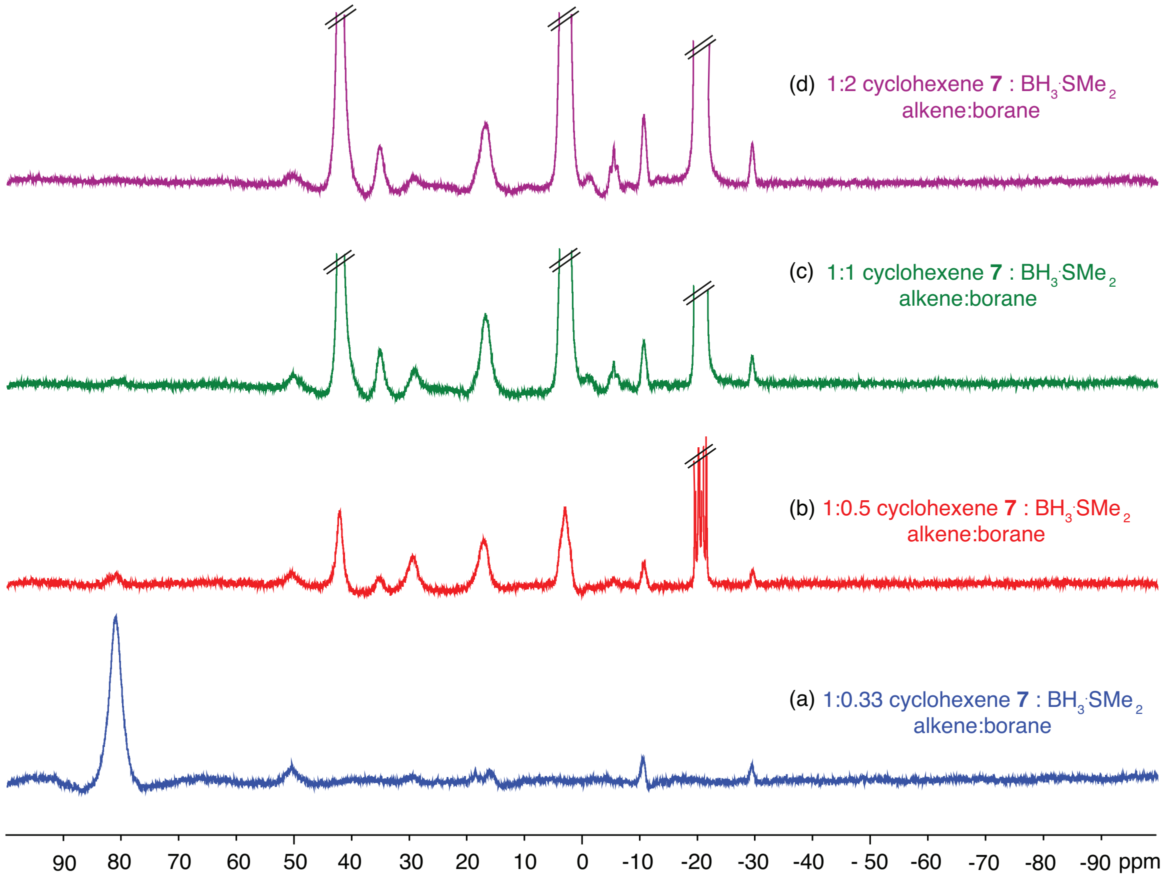

Figure S15: The ^{11}B NMR spectra of the alkene to boron hydroborations of cyclohexene **7** with (a) 0.33 (b) 0.5 (c) 1 and (d) 2 equivalents of borane $BH_3{\cdot}SMe_2$ in THF.

Based on the data obtained throughout this study, the stable and preferably formed during hydroboration $(R_2BH)_2$ species, reacts with borane $BH_3{\cdot}SMe_2$ to form R_2BHBH_3 species. The latter disproportionates or scrambles (or even further hydroborates) in short periods of time, to form R_2BHBH_2R which in turn disproportionates or scrambles to form $(RBH_2)_2$. The $(RBH_2)_2$ can be converted back to the initial $(R_2BH)_2$ species by further hydroboration (see Figure S17). Although the synthesis and isolation of $(RBH_2)_2$ species appeared impossible through simple uncontrolled hydroboration, a careful synthetic strategy can be devised using the above cycle, in order to obtain such species.

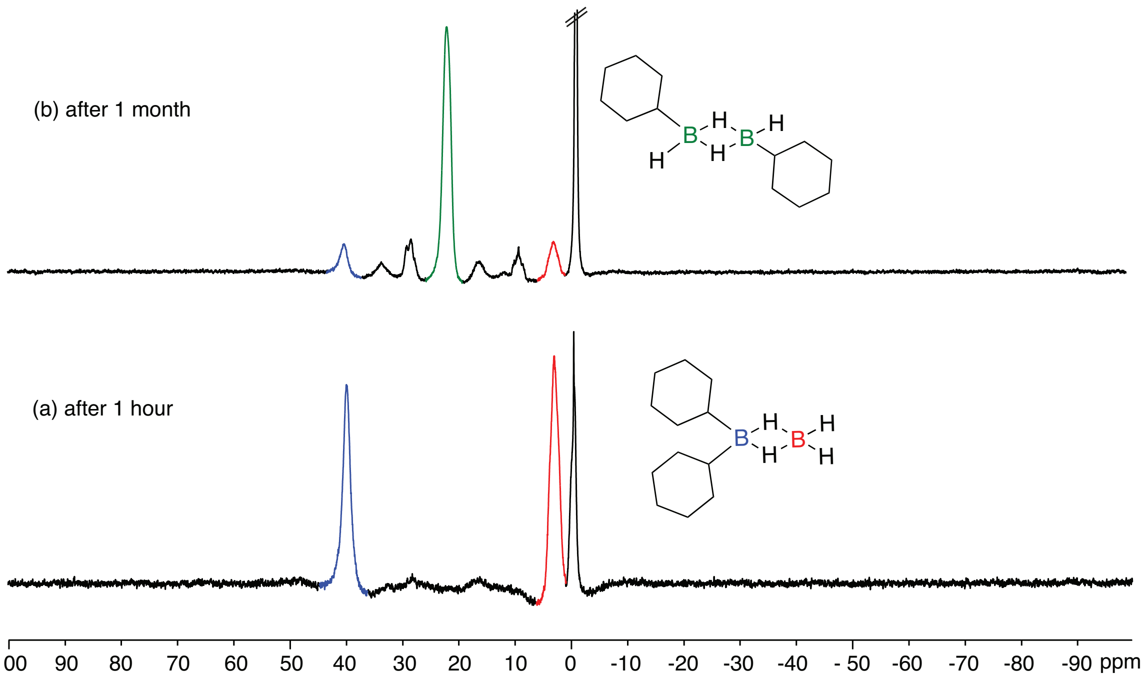

Figure S16: The ^{11}B NMR spectra of the hydroboration of cyclohexene **7** with diborane(6) gas in diglyme at (a) 1h and (b) after 1 month.

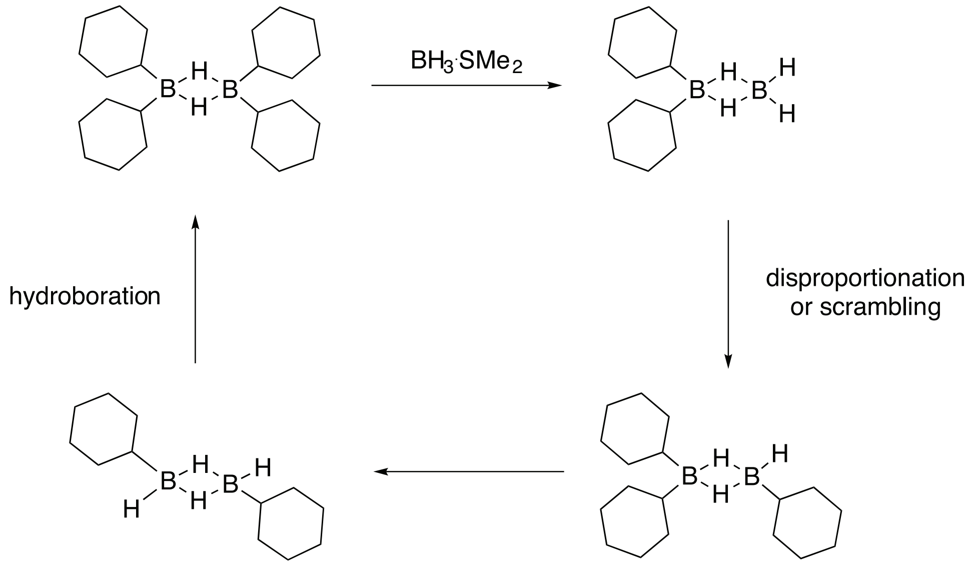

Figure S17: The inter converting cycle of the different boranes formed during hydroboration, based on the data obtained in this study.

To illustrate the kinetic *vs* thermodynamic nature of **9** and **8**, a kinetic study was performed, altering the rate of addition of either the borane or alkene reagent. Due to the already fast reaction time scales of hydroboration, only four different addition times were explored, using a controlled syringe pump and adding, depending on the mode of addition, the reagent: instantly, over one, five or ten minutes (in diethyl ether), while keeping the total reaction time constant. Dicyclohexyl borane **9** precipitated out of solution as a white powder and the remaining filtrate contained mainly tricyclohexylborane **8** R_3B with a signal at +81 ppm and small amounts of an unknown species at +50 ppm (for more details see below).

As seen in Figure S18 during slower than dropwise additions, the mode of reagent addition was highly influential on the stoichiometry of the products formed. When the borane $BH_3{\cdot}SMe_2$ was added to the alkene, the yield of dicyclohexylborane **9** $(R_2BH)_2$ decreased dramatically (80% at instant addition, 71% at 1 minute, 44% at 5 minutes and 17% at 10 minutes) while the addition time of borane was increased. On the other hand, when the alkene was added to the borane, the reaction was proved to be more complicated with an unclear trend of an initial yield decrease, followed by a plateau and a second increase of the dicyclohexylborane **9** $(R_2BH)_2$ species formed (43% at instant addition, 38% at 1 minute, 39% at 5 minutes and 53% at 10 minutes). This clearly indicates that controlled reagent addition leads to the formation of insoluble materials with different borane species and therefore, different chemical and physical properties.

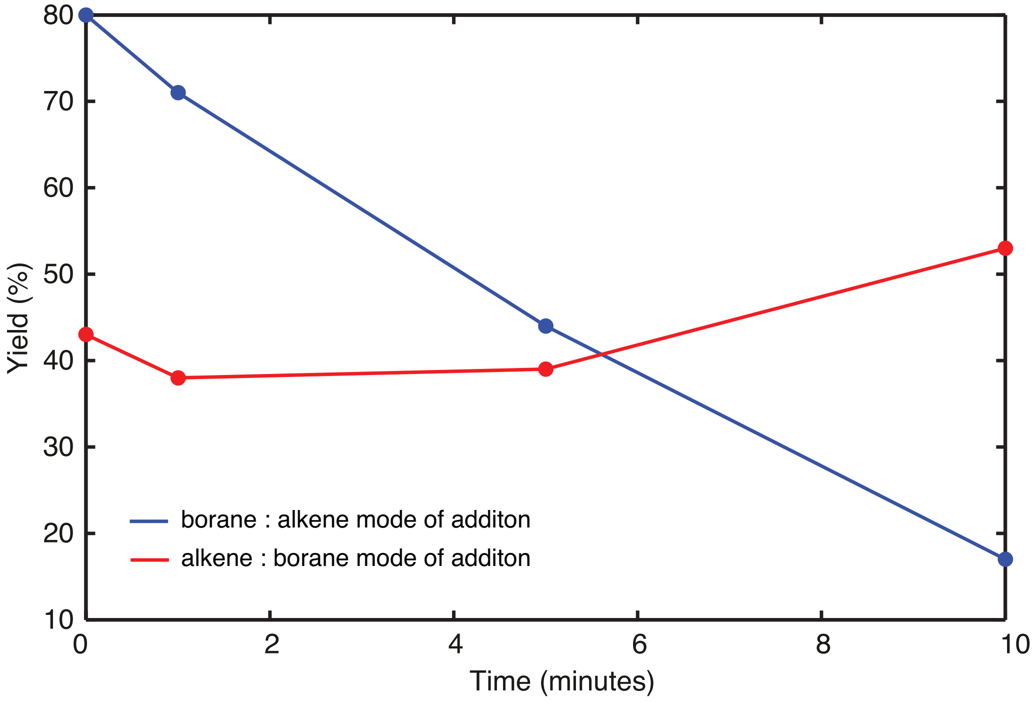

Figure S18: The yield of dicyclohexylborane **9** $(R_2BH)_2$ formed over the different addition times of (a) in blue, borane $BH_3{\cdot}SMe_2$ to cyclohexene **7** and (b) in red, cyclohexene **7** to borane $BH_3{\cdot}SMe_2$ in diethyl ether.

It was suspected that the species observed at approximately +50 ppm in the ^{11}B NMR in various reactions, could be the $R_3B{\cdot}SMe_2$ complex as the ppm difference between this signal and the free R_3B is similar to the difference between $(R_2BH)_2$ and $R_2BH{\cdot}SMe_2$ and $(RBH_2)_2$ and $RBH_2{\cdot}SMe_2$. Unfortunately, the results were inconclusive as addition of SMe_2 to R_3B, as seen in Figure S19, resulted in the formation of two species at around +50 ppm. It is unclear whether both signals correspond to such $R_3B{\cdot}SMe_2$ species or whether one of them corresponds to another unknown species. Additionally, and supporting the $R_3B{\cdot}SMe_2$ complex hypothe-

sis, the presence of this species was greatly reduced when a vacuum was applied to the sample, indicating that perhaps when the right conditions for the volatile SMe_2 are introduced, the species reverts back to the free R_3B or alternatively that this species is volatile. Moreover, addition of triphenylphosphine to solutions containing this species, did not yield any phosphorous containing complexes, suggesting that this species is most probably not a boron hydride.[6] Finally, analysis of a ^{11}B NMR spectrum, containing this species, with ^{1}H decoupling showed no effect on the size or the half width of the signal suggesting, as before, the absence of a B-H bond. The exact nature of this species remains unknown.

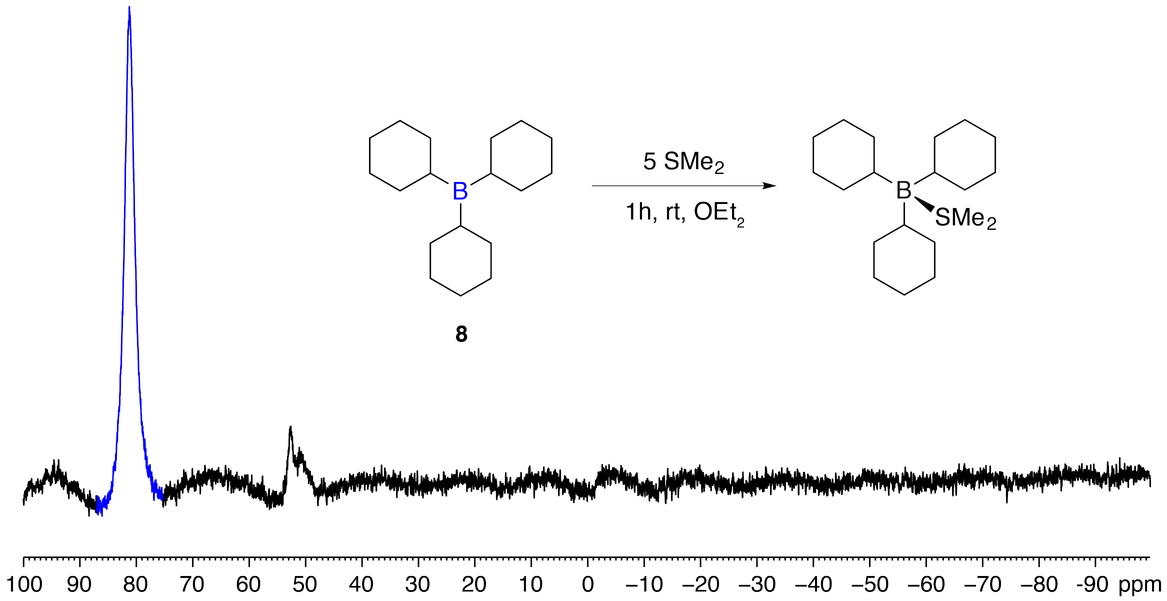

Figure S19: The reaction and suspected products formed upon addition of SMe_2 to a R_3B species and the ^{11}B NMR spectrum of the reaction.

S.7.3 Solid state NMR

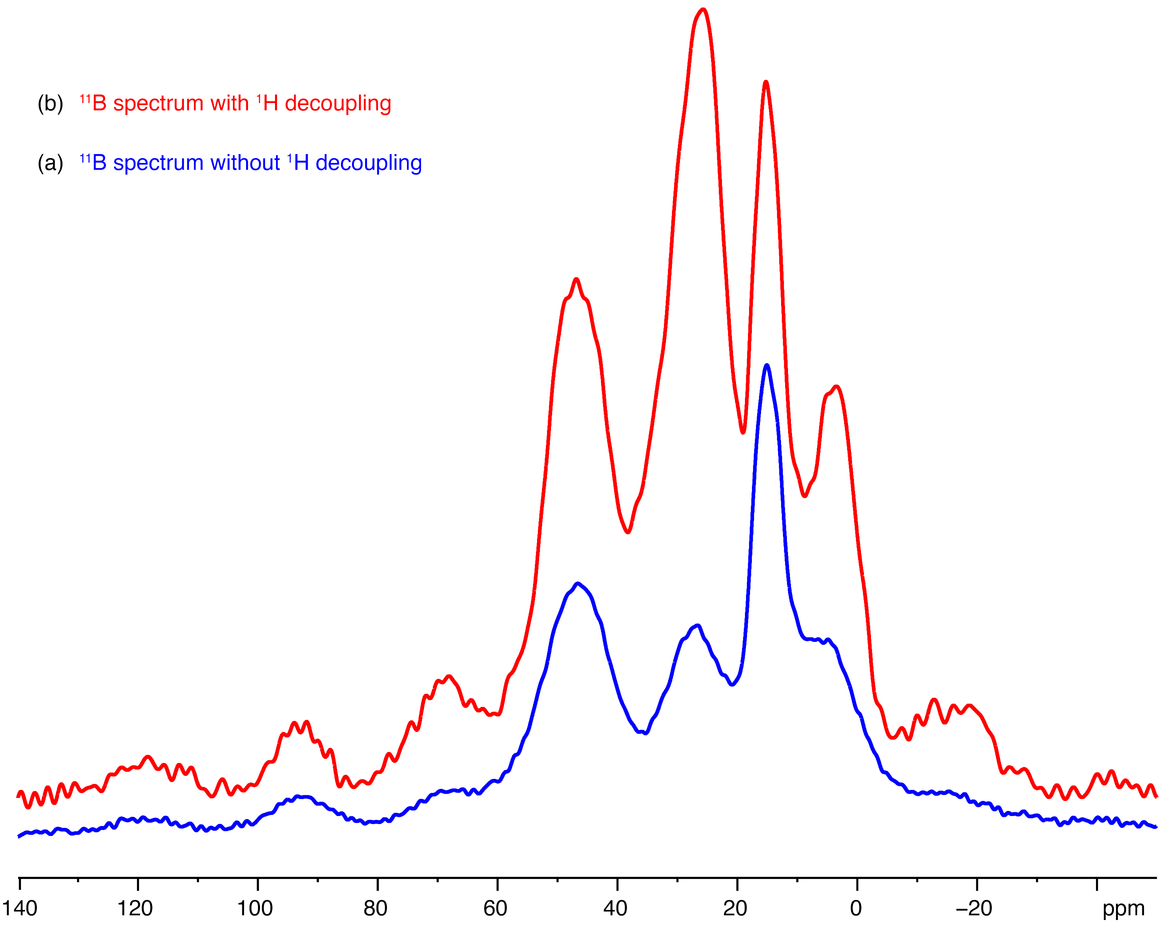

Figure S20: The solid state ^{11}B NMR of the solid formed from the reaction between 1:1 B_2H_6 and 1,3-cyclohexadiene **1** (a) without (blue) and (b) with ^{1}H decoupling (red).

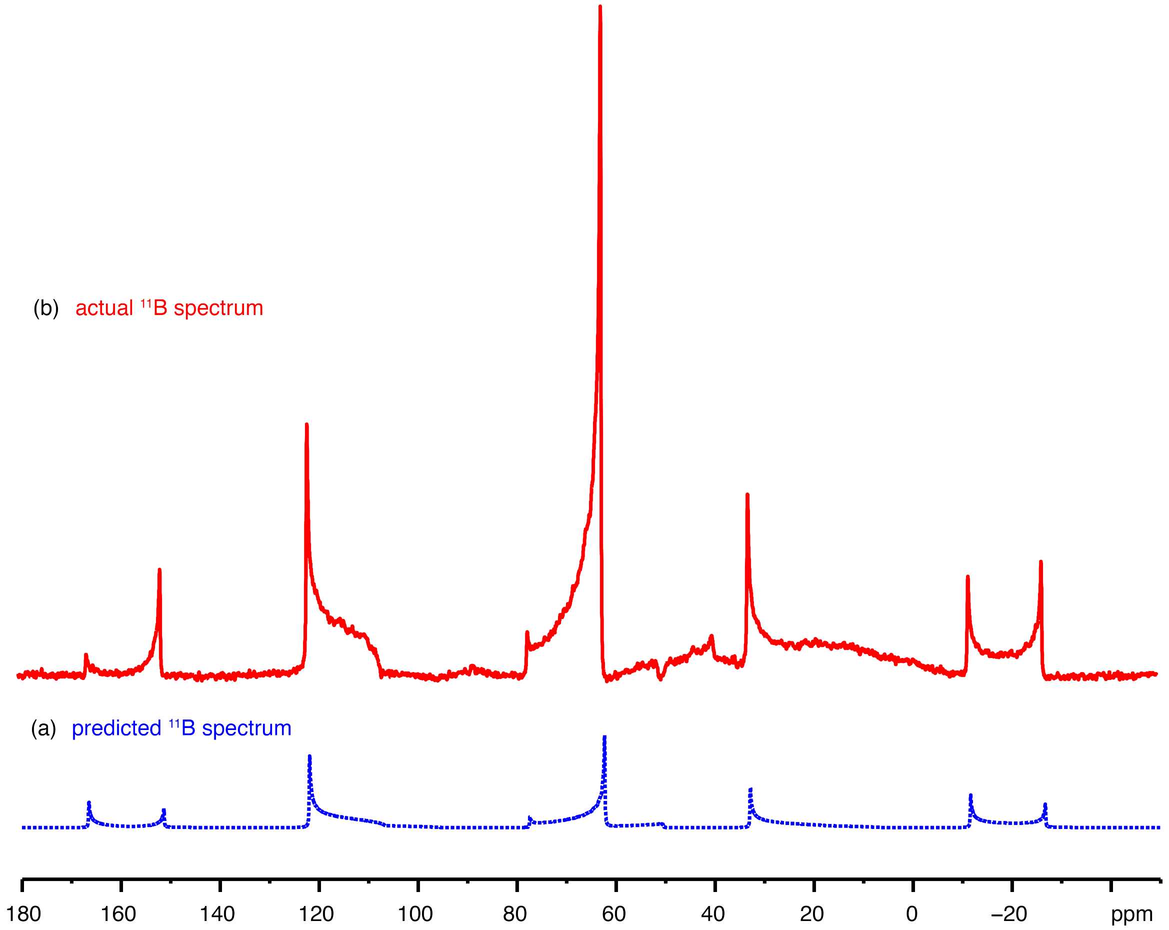

Figure S21: The (a) simulated (blue) and (b) experimental (red) solid state ^{11}B NMR of the model compound tricyclohexylborane **8** R_3B.

The ^{11}B spectrum of the trigonal compound can be fitted with a relatively large quadrupolar (the coupling parameter Cq = 5.05 MHz with asymmetry of 0) and chemical shift anisotropy (anisotropy of -90 ppm and asymmetry of 0). At a field of 16.4 T the second order quadrupolar broadening is about 28 kHz and the chemical shift anisotropy is 20.7 kHz, both of which cannot be efficiently reduced at a moderate spinning frequency of 10 kHz. The isotropic resonance (corresponding to the centre of gravity of the central transition in the spectrum) is shifted from the isotropic chemical shift position by the second order isotropic quadrupolar shift which is equal to -12.6 ppm, resulting in a total isotropic shift of +69.4 ppm.

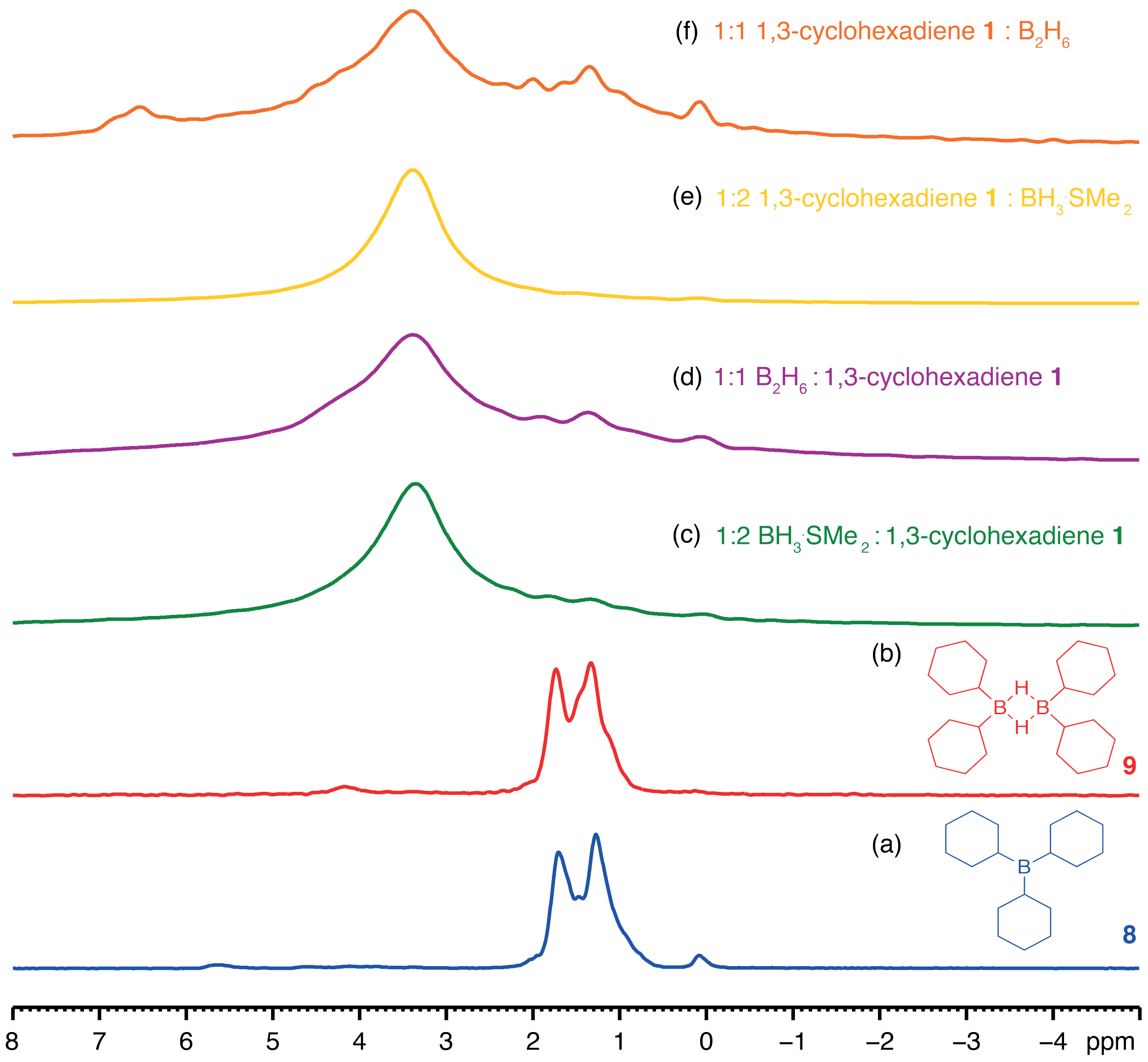

Figure S22: The solid state ^{1}H NMR spectra of a range of different materials formed during the hydroboration of 1,3-cyclohexadiene **1** with 1 or 2 equivalents of borane $BH_3{\cdot}SMe_2$ and diborane(6) B_2H_6 gas in diglyme.

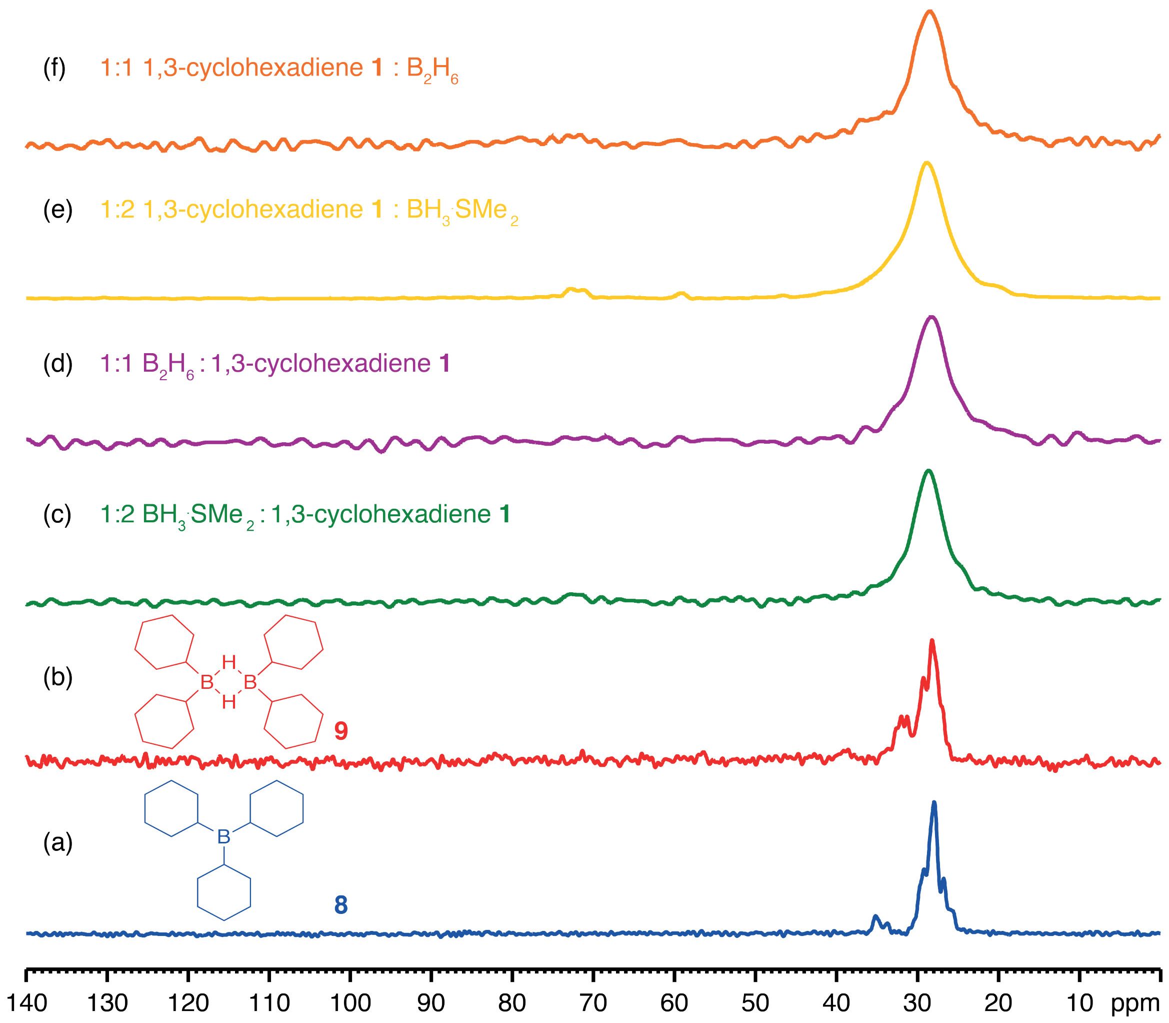

Figure S23: The solid state NMR ^{1}H-^{13}C CP MAS spectra of a range of different materials formed during the hydroboration of 1,3-cyclohexadiene **1** with 1 or 2 equivalents of borane $BH_3{\cdot}SMe_2$ and diborane(6) B_2H_6 gas in diglyme.

S.7.4 Analysis of the oxidised products by GC-MS

GC-MS data obtained upon oxidation of the insoluble materials obtained during the dihydroboration of 1,3-cyclohexadiene following our conditions.

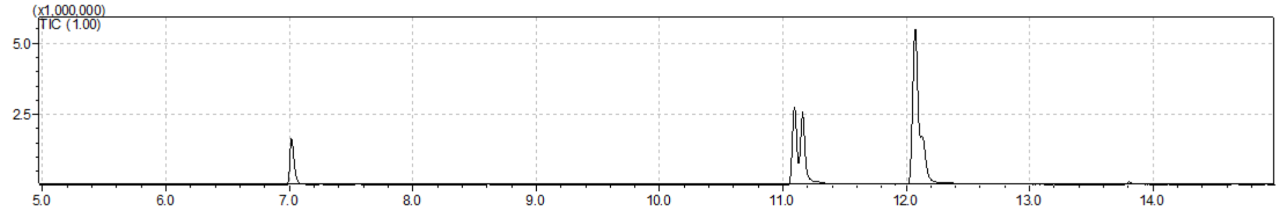

Figure S24: Oxidation products of the insoluble material obtained from the hydroboration of 1 equiv. of 1,3-cyclohexadiene **1** with 2 equivs. of $BH_3 \cdot SMe_2$ using the borane to diene reagent addition mode in THF.

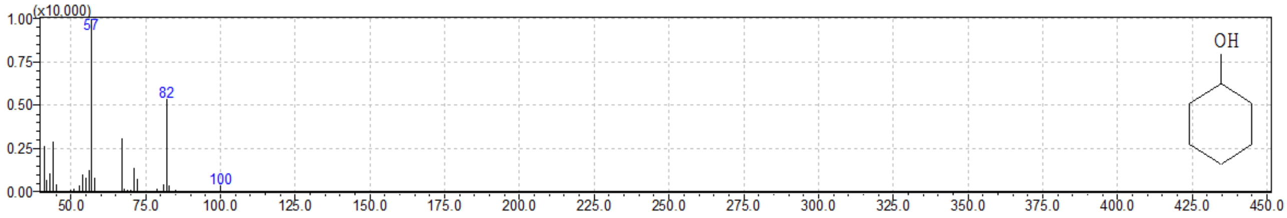

Figure S25: Peak 1 identification.

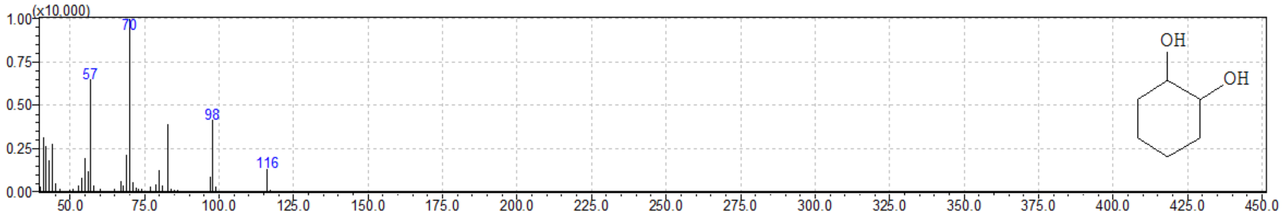

Figure S26: Peak 2 identification.

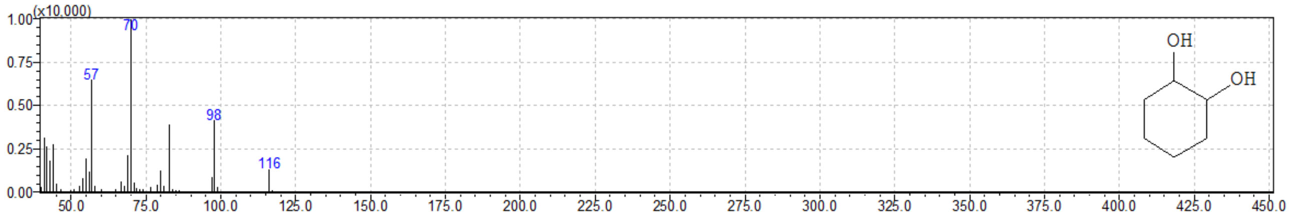

Figure S27: Peak 3 identification.

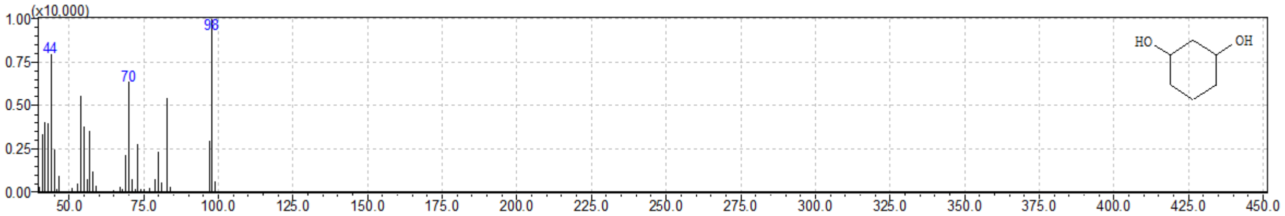

Figure S28: Peak 4 identification.

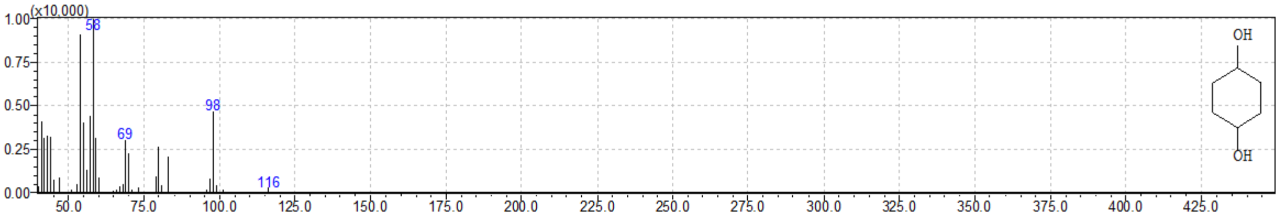

Figure S29: Peak 5 identification.

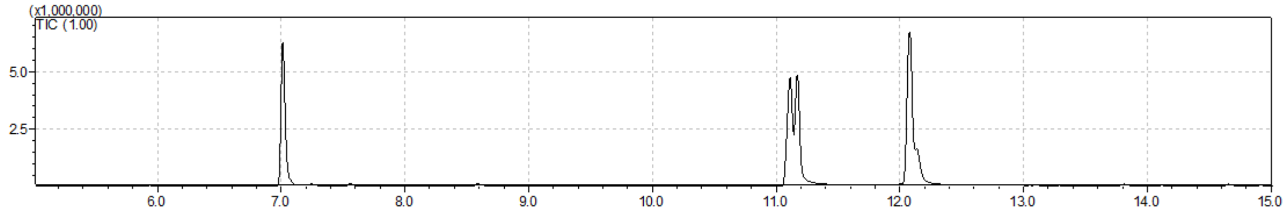

Figure S30: Oxidation products of the insoluble material obtained from the hydroboration of 1 equiv. of 1,3-cyclohexadiene **1** with 2 equivs. of $BH_3{\cdot}SMe_2$ using the diene to borane reagent addition mode in THF.

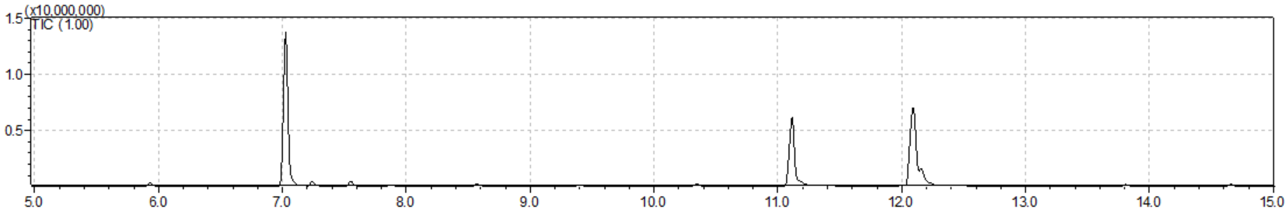

Figure S31: Oxidation products of the insoluble material obtained from the hydroboration of 1 equiv. of 1,3-cyclohexadiene **1** with 1 equiv. of $BH_3{\cdot}SMe_2$ using the borane to diene reagent addition mode in THF.

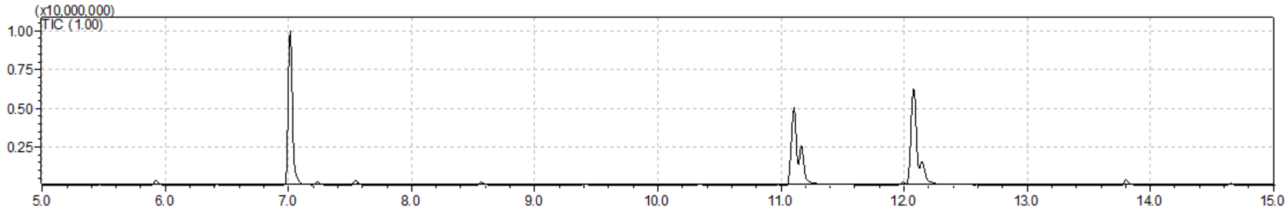

Figure S32: Oxidation products of the insoluble material obtained from the hydroboration of 1 equiv. of 1,3-cyclohexadiene **1** with1 equiv. of $BH_3{\cdot}SMe_2$ using the diene to borane reagent addition mode in THF.

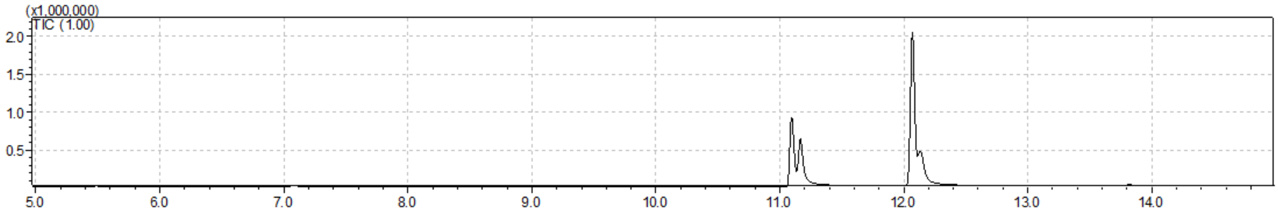

Figure S33: Oxidation products of the insoluble material obtained from the hydroboration of 1 equiv. of 1,3-cyclohexadiene **1** with 2 equivs. of $BH_3 \cdot SMe_2$ using the borane to diene reagent addition mode in diglyme.

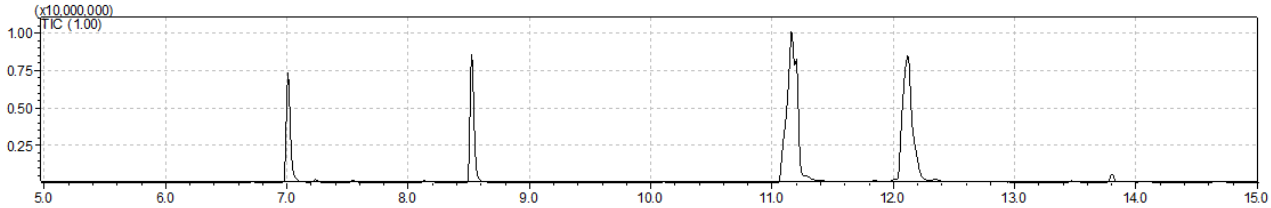

Figure S34: Oxidation products of the insoluble material obtained from the hydroboration of 1 equiv. of 1,3-cyclohexadiene **1** with 2 equivs. of $BH_3 \cdot SMe_2$ using the borane to diene reagent addition mode in diglyme.

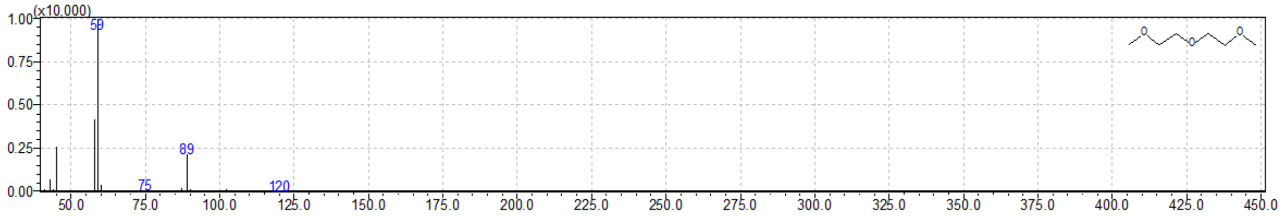

Figure S35: Peak 2 identification.

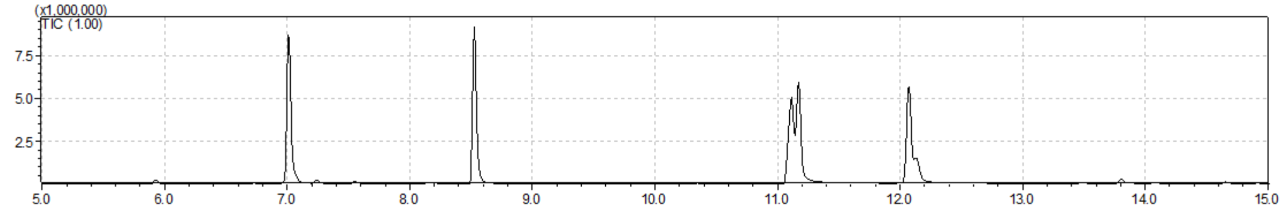

Figure S36: Oxidation products of the insoluble material obtained from the hydroboration of 1 equiv. of 1,3-cyclohexadiene **1** with1 equivs. of $BH_3 \cdot SMe_2$ using the borane to diene reagent addition mode in diglyme.

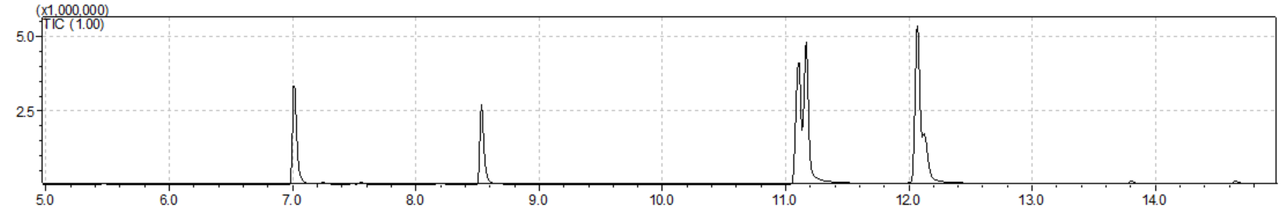

Figure S37: Oxidation products of the insoluble material obtained from the hydroboration of 1 equiv. of 1,3-cyclohexadiene **1** with1 equivs. of $BH_3 \cdot SMe_2$ using the diene to borane reagent addition mode in diglyme.

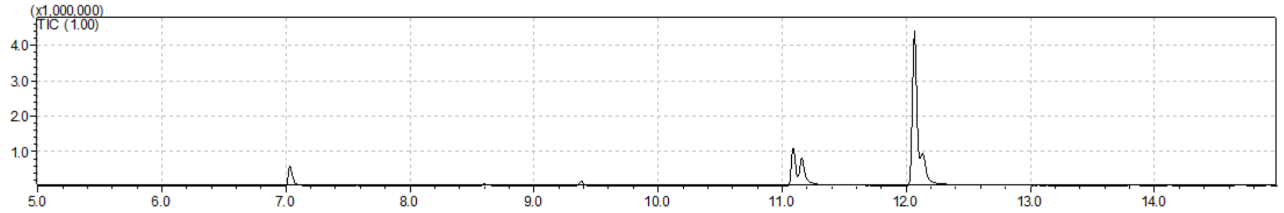

Figure S38: Oxidation products of the insoluble material obtained from the hydroboration of 1 equiv. of 1,3-cyclohexadiene **1** with 1 equiv. of diborane gas using the slow diborane release mode in diglyme.

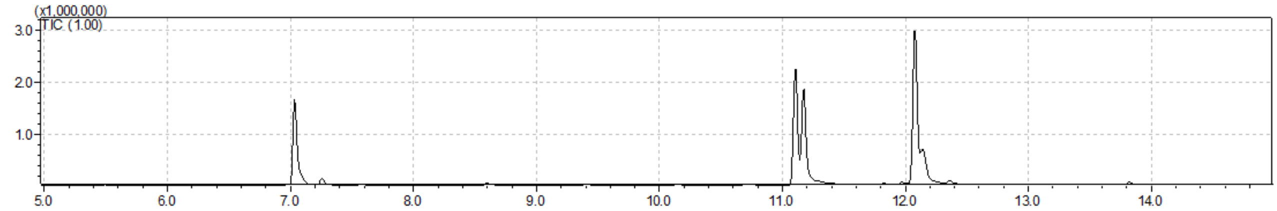

Figure S39: Oxidation products of the insoluble material obtained from the hydroboration of 1 equiv. of 1,3-cyclohexadiene **1** with 1 equiv. of diborane gas using the fast diborane release mode in diglyme.

Oxidation products formed using the literature reported conditions as described by H.C. Brown *et al.*[3]

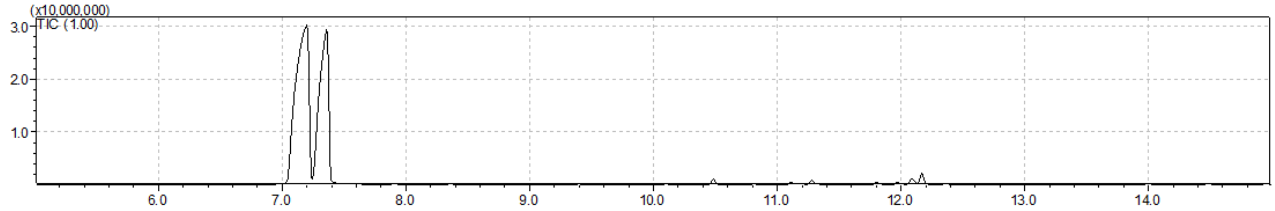

Figure S40: Oxidation products formed upon oxidation of the hydroboration mixture obtained while following the reported hydroboration conditions and utilising the borane to diene addition mode.

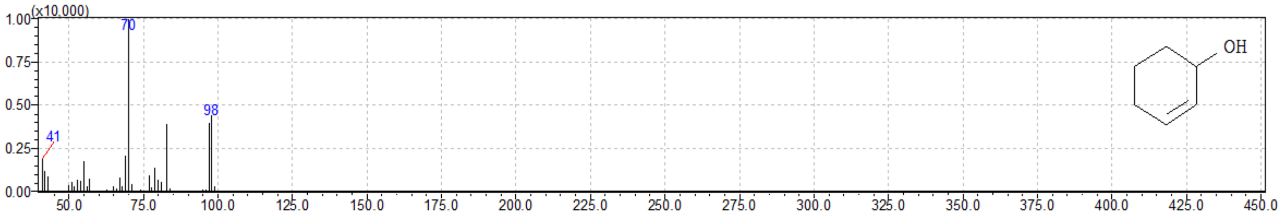

Figure S41: Peak 1 identification.

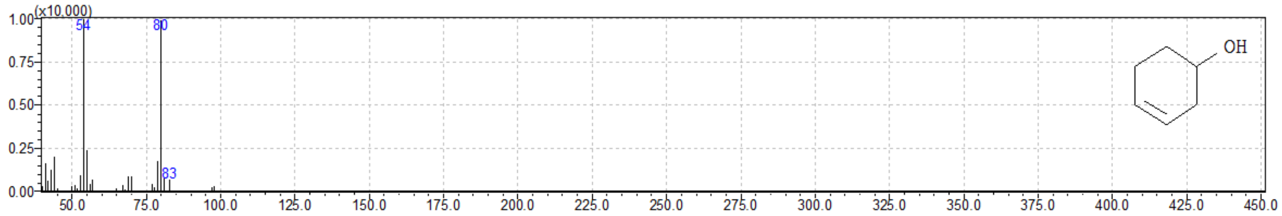

Figure S42: Peak 2 identification.

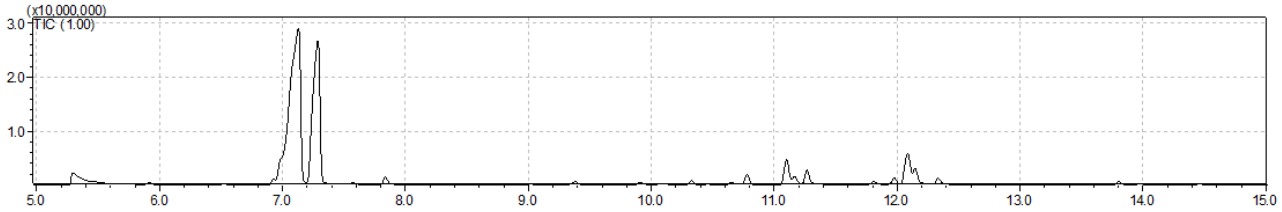

Figure S43: Oxidation products formed upon oxidation of the hydroboration mixture obtained while following the reported hydroboration conditions and utilising the diene to borane addition mode.

Control oxidation reactions.

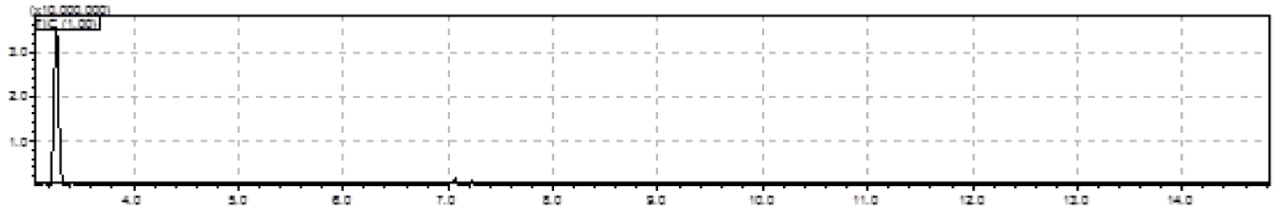

Figure S44: Products obtained upon oxidation of 1,3-cyclohexadiene **1** (=1,3-cyclohexadiene **1**).

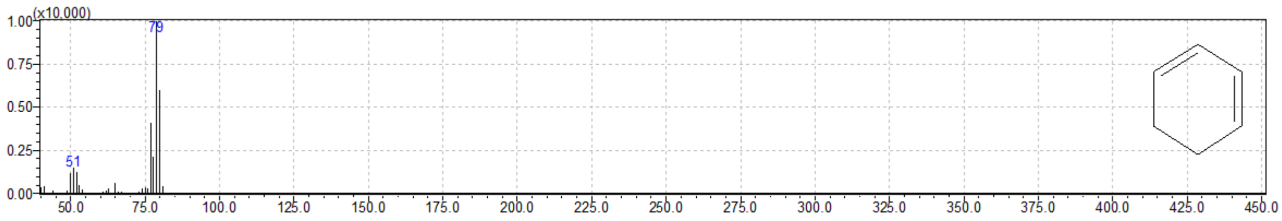

Figure S45: Peak 1 identification.

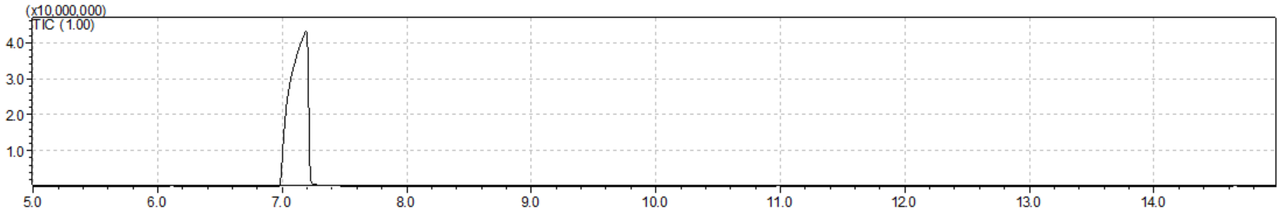

Figure S46: Oxidation products obtained upon oxidation of the model compound dicyclohexylborane **9**.

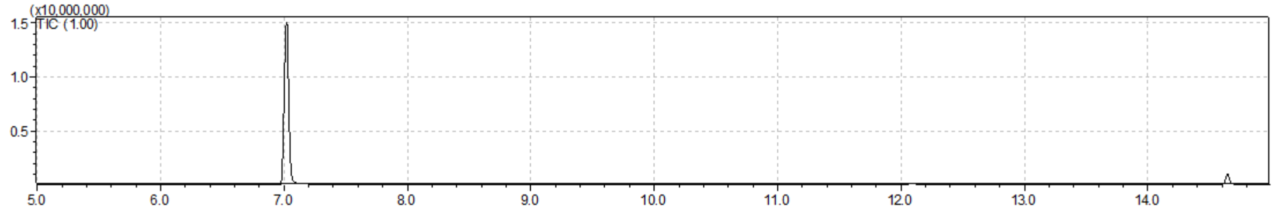

Figure S47: Oxidation products obtained upon oxidation of the model compound tricyclohexylborane **8**.

S.8 Proposed mechanism

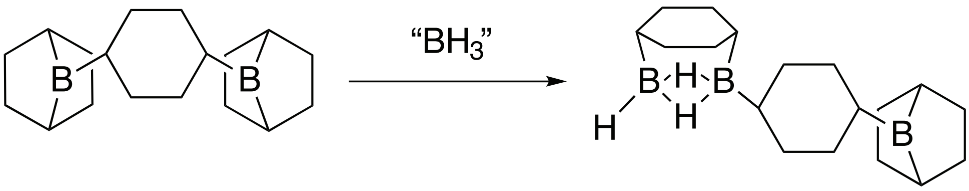

Figure S48: Highly unfavourable ring-in-ring structures *via* cyclic hydroboration of 1,3-cyclohexadiene **1**.

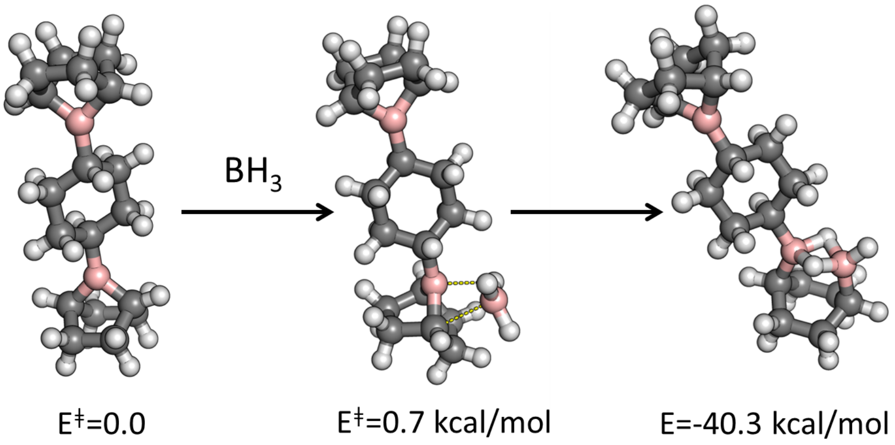

Figure S49: Activation energies and exothermicities of the hydroboration of the ring-in-ring structures. Energies are given in kcal/mol relative to the isolated reactants. Stationary points were optimized at the B3LYP/6-31G* level of theory and the energies are reported at the B3LYP/6-311++G** level of theory. Vibrational frequencies were calculated to ensure that each minimum is a true local minimum and that each transition state has a single imaginary frequency.

S.9 Hydroboration of other dienes

In order to determine if all cyclic dienes yield the formation of these materials we also investigated the hydroboration of a range of different 1,3- ,1,4- and 1,5- cyclic dienes as well as 1,3-butadiene, as seen in Figure S50, while using borane $BH_3{\cdot}SMe_2$ in diglyme in the exact concentration and conditions as used in the diborane(6) experiments. The different dienes shown in Figure S50 were reacted with one and two equivalents of borane $BH_3{\cdot}SMe_2$, as some of them were not expected to yield white precipitates and in those cases it was of interest to identify the boron species present in solution. All reactions were performed using the practically easier boron to diene addition mode, as the reagent addition mode has been repeatedly shown to be of little influence during dropwise addition over a minute.

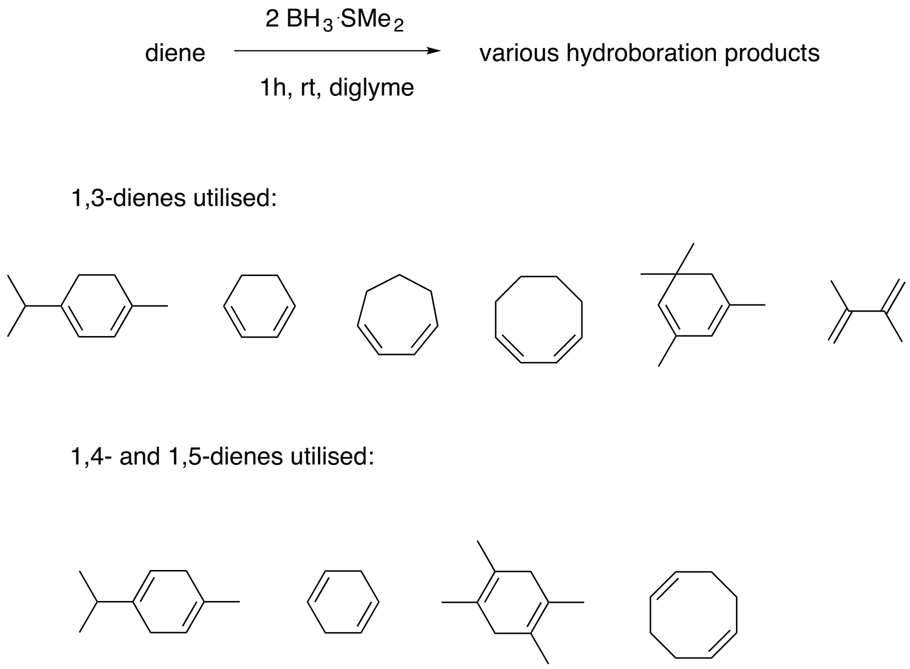

Figure S50: The hydroboration conditions and dienes used in this study.

The ^{11}B NMRs of the clear solutions, and therefore, the ones not yielding white precipitates are shown in (a) Figure S51 when one borane equivalent was utilised and (b) Figure S52 when two borane equivalents were utilised.

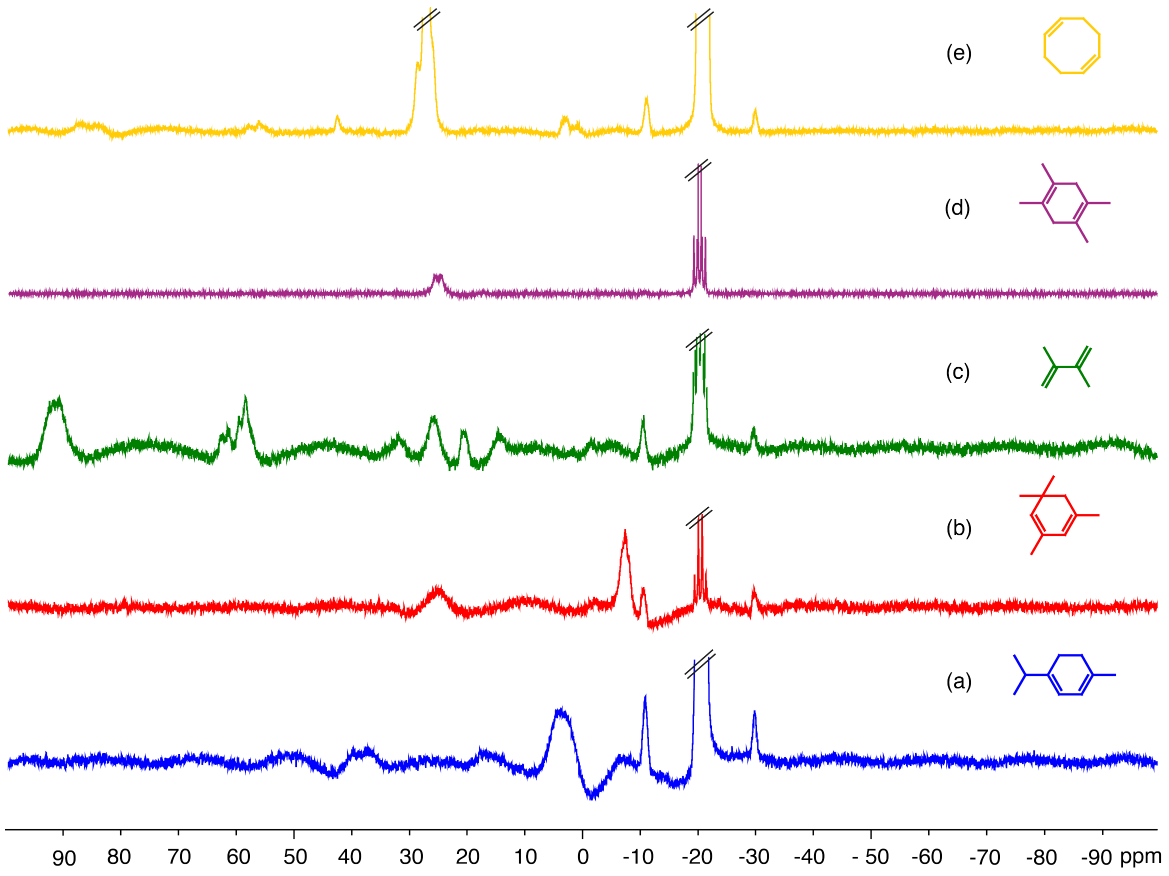

Figure S51: The ^{11}B NMR spectra of the clear solutions from the hydroborations of (a) α-terpinene, (b) 1,3,5,5-tetramethyl-1,3-cyclohexadiene, (c) 2,3-dimethyl-1,3-butadiene, (d) 1,2,4,5-tetramethyl-1,4-cyclohexadiene and (e) 1,5-cyclooctadiene with one equivalent of borane $BH_3 \cdot SMe_2$ in diglyme.

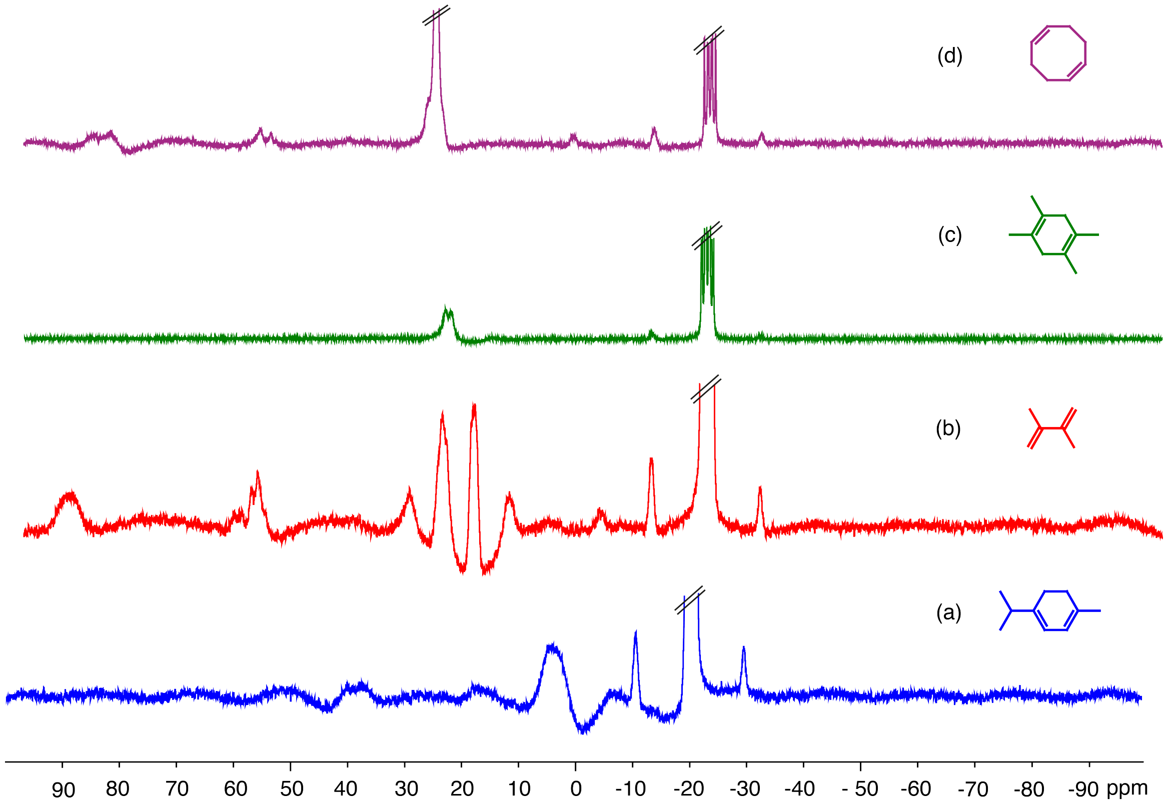

Figure S52: The ^{11}B NMR spectra of the clear solutions from the hydroborations of (a) α-terpinene, (b) 2,3-dimethyl-1,3-butadiene, (c) 1,2,4,5-tetramethyl-1,4-cyclohexadiene and (d) 1,5-cyclooctadiene with two equivalents of borane $BH_3{\cdot}SMe_2$ in diglyme.

S.10 Comparison between the reported hydroboration conditions and ours

It was clear that monohydroboration is dominant under the current literature conditions,[3] whereas full dihydroboration is observed under our conditions. Direct comparison of their conditions over ours, revealed a concentration difference by a factor of two with a concentration of 1.33M as opposed to 0.69M in our conditions. However, since our system is essentially more dilute, concentration could not explain this reactivity different as a reverse effect would be expected. Additionally, our system was also explored in a more dilute concentration (by a factor of five, 0.14M) and was observed to still promote dihydroboration.

Additionally, different equivalent ratios were used in these two studies. For a reason that remains unclear, H.C. Brown and K. Bhat utilised 10 mmol of 1,3-cyclohexadiene **1** and 3.33 mmol of borane $BH_3{\cdot}SMe_2$. Depending on the point of view and compared to our conditions, an excess of 8.335 mmol of diene were used, which equals a 6 equivalent excess resulting in a 3:1 molar ratio of diene:borane. Alternatively, their system is depleted by 16.67 mmol of borane $BH_3{\cdot}SMe_2$, which equals a 6 equivalent shortage. However, the equivalent difference in the reagents used is not expected to have such a high effect on the reaction products since in our study a range of different equivalents were explored and have all shown to promote dihydroboration irrespectively (unless a large excess of diene is used).

During our studies, it was evident that the reaction pathway is highly dependant on the reagent equivalents. When an excess of diene is used, monohydroboration is dominant, whereas when an excess of borane is used, dihydroboration is dominant, both to high selectivity. As a result, we believe that the difference between our

observations and the ones published could be due to differences in the diene/borane equivalents employed.

It is also possible, that their borane $BH_3 \cdot SMe_2$ solution was not as pure as thought, as it was commercially purchased. Despite their precautions of re-determining the molarity of the purchased solutions, it is possible that a species present in these impure solutions inhibits the formation of these materials. A similar effect was also observed in our system, specifically when commercially available borane $BH_3 \cdot THF$ was used, leading to significantly lower formation of these materials suggesting that the formation of networks is sensitive to impurities, a phenomenon that is known in some cases of polymer formation. However, full utilisation of the diene, leading to dihydroboration, was still observed.

References

[1] Moffett, R. *Organic Syntheses*, 1st ed.; Wiley, 1963.

[2] Fung, B.; Khitrin, A.; Ermolaev, K. *J. Magn. Reson.* **2000**, *142*, 97–101.

[3] Brown, H.; Bhat, K. *J. Org. Chem.* **1986**, *51*, 445–449.

[4] Freeguard, C.; Long, L. *Chem. Ind.* **1965**, 471.

[5] Kanth, J.; Brown, H. *Inorg. Chem.* **2000**, *39*, 1795–1802.

[6] Andreou, A. Synthesis and Reactivity of Boron Hydrides for the Preparation of Chiral Diboranes and Bisphosphines. Ph.D. thesis, University of Cambridge: UK, 2013.

S.11 Theoretical calculations

1. Reactivity of substituted boranes. Reaction pathways.

To understand the reaction mechanism leading to polymer formation, we quantitatively establish the following:

a. We find that the hydroboration on the first double bond is kinetically more favourable than on the second double bond. This is in good agreement with the fact that only the first double bond is substituted in case of limiting amounts of borane.
b. A key factor in the reactivity during the first steps of the hydroboration reaction is related to forming the monomeric borane intermediate. Due to this, the mono-substituted immediate reaction product (RBH_2) is a high energy species that will quickly react with double bonds, or it will be stabilized by dimer or solvent adduct formation.
c. The di-substituted borane (R_2BH) is significantly less reactive even in its monomeric form (last line in Table S1 and Figure S55).

The observations corresponding to b. and c. are consistent with the formation of a polymer that has mainly B-R-BH-R-BH-R… chains with di-substituted borane.

Table S1: Activation energies and exothermicities of hydroboration reactions. Energies are given in kcal/mol relative to the isolated reactants. Stationary points were optimized at the B3LYP/6-31G* level of theory and the energies are reported at the B3LYP/6-311++G** level of theory. Vibrational frequencies were calculated to ensure that each minimum is a true local minimum and that each transition state has a single imaginary frequency.

Reaction	TS_1 (kcal/mol)	IS (kcal/mol)	TS_2 (kcal/mol)	PS (kcal/mol)
$BH_3.SMe_2$ + **7** → **11a**	13.8	12.9	16.1	-5.2
$BH_3.SMe_2$ + **1** → **13a**	15.0[a]	14.6	16.8	-5.3[a]
$BH_3.SMe_2$ + **1** → **13b**	15.0[a]	14.6	16.9	-3.0
$BH_3.SMe_2$ + **13a** → 1-2,diboro-cyclohexane<br>Intramolecular:<br>Intermolecular	 11.0[b]<br>14.6[b]	 6.8[b]<br>13.9[b]	 15.5[b]<br>18.3	 -10.0<br>-2.4
$BH_3.SMe_2$ + **13a** → 1-3,diboro-cyclohexane<br>Intramolecular:<br>Intermolecular:	 11.0[b]<br>14.6[b]	 6.8[b]<br>13.9[b]	 18.0[b]<br>17.7	 -21.8[b]<br>-2.9
**11a** + **7** → dicyclohexyl-borane	11.1[c]	10.9	15.8	-11.7
**9** + **7** → **8**	N/A	N/A	20.4	-19.9

[a]Structure displayed in Figure S53
[b]Structure displayed in Figure S54
[c]Result obtained from reaction coordinate scanning

Table S2: Dissociation energies of the $R_3B.SMe_2$ complexes. Energies are given in kcal/mol relative to the optimized complex. Stationary points were optimized at the B3LYP/6-31G* level of theory and the energies are reported at the B3LYP/6-311++G** level of theory. Vibrational frequencies were calculated to ensure that each minimum is a true local minimum.

Molecule	ΔE_{diss}
$BH_3.SMe_2$	26.1
**13a**.SMe_2	13.1
**13b**.SMe_2	14.0
**11a**.SMe_2	13.3

Table S3: Energies and structures of the calculated transition states for the **13(a-b)** + **1** and **13a** + **13b** reactions. Energies are given in kcal/mol relative to the isolated reactants. Stationary points were optimized at the B3LYP/6-31G* level of theory. Vibrational frequencies were calculated to ensure that each minimum is a true local minimum and that each transition state has a single imaginary frequency.

Reaction	TS_2 (kcal/mol)
**13a** + **1**	1.8 (S3a)<br>2.2 (S3b)
**13b** + **1**	1.0 (S3c)<br>1.4 (S3d)
**13a** + **13b**	4.0 (S3e)<br>4.0 (S3f)<br>4.3 (S3g)

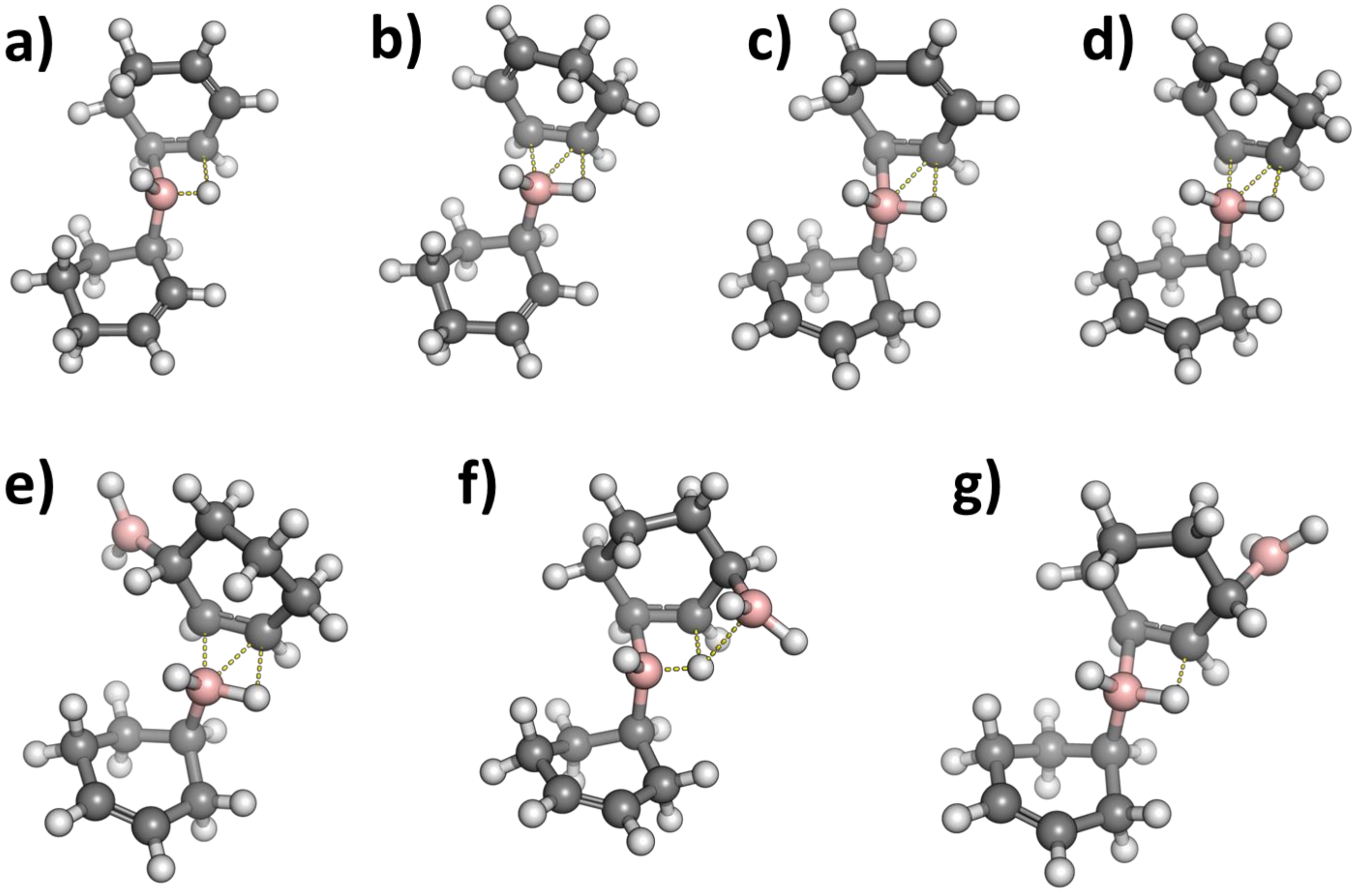

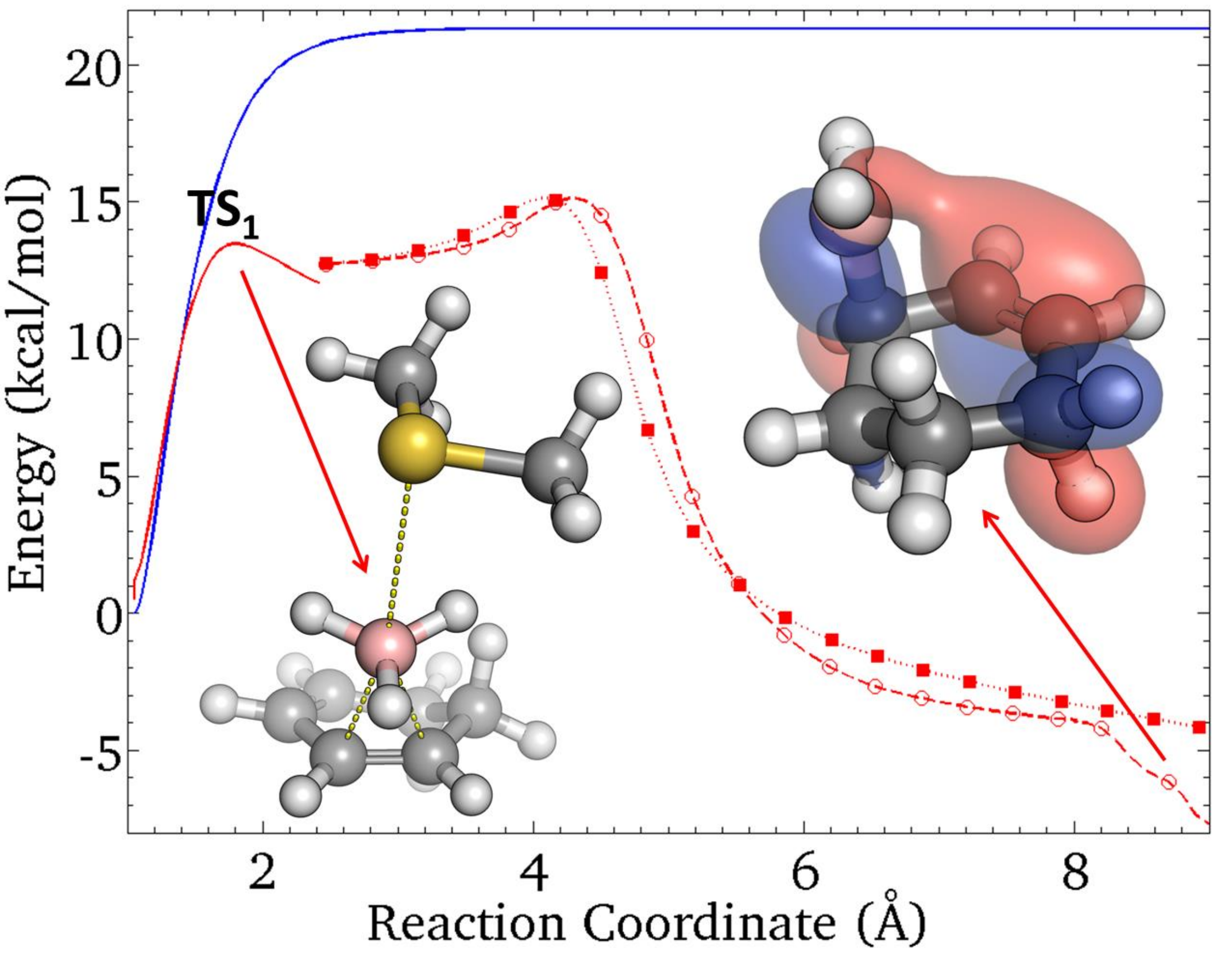

Figure S53: Dissociation of the BH3·SMe2 complex. The dissociation profile is shown in the absence of electron donors ($\Delta E^{\ddagger}$ = 21.4 kcal/mol, blue line), and in the presence of **1** (TS1 = 13.5 kcal/mol, solid red line and left inset). The second step of the hydroboration of **1** to produce **13a** (red squares) and **13b** (red open circles) is also shown. The HOMO orbital of the more stable axial **13a** product shows delocalization of the C=C double bond to the B 2p orbital (right inset). The reaction paths have been calculated by integrating the intrinsic reaction coordinate (red curves) or by scanning the B-S distance (blue curve).

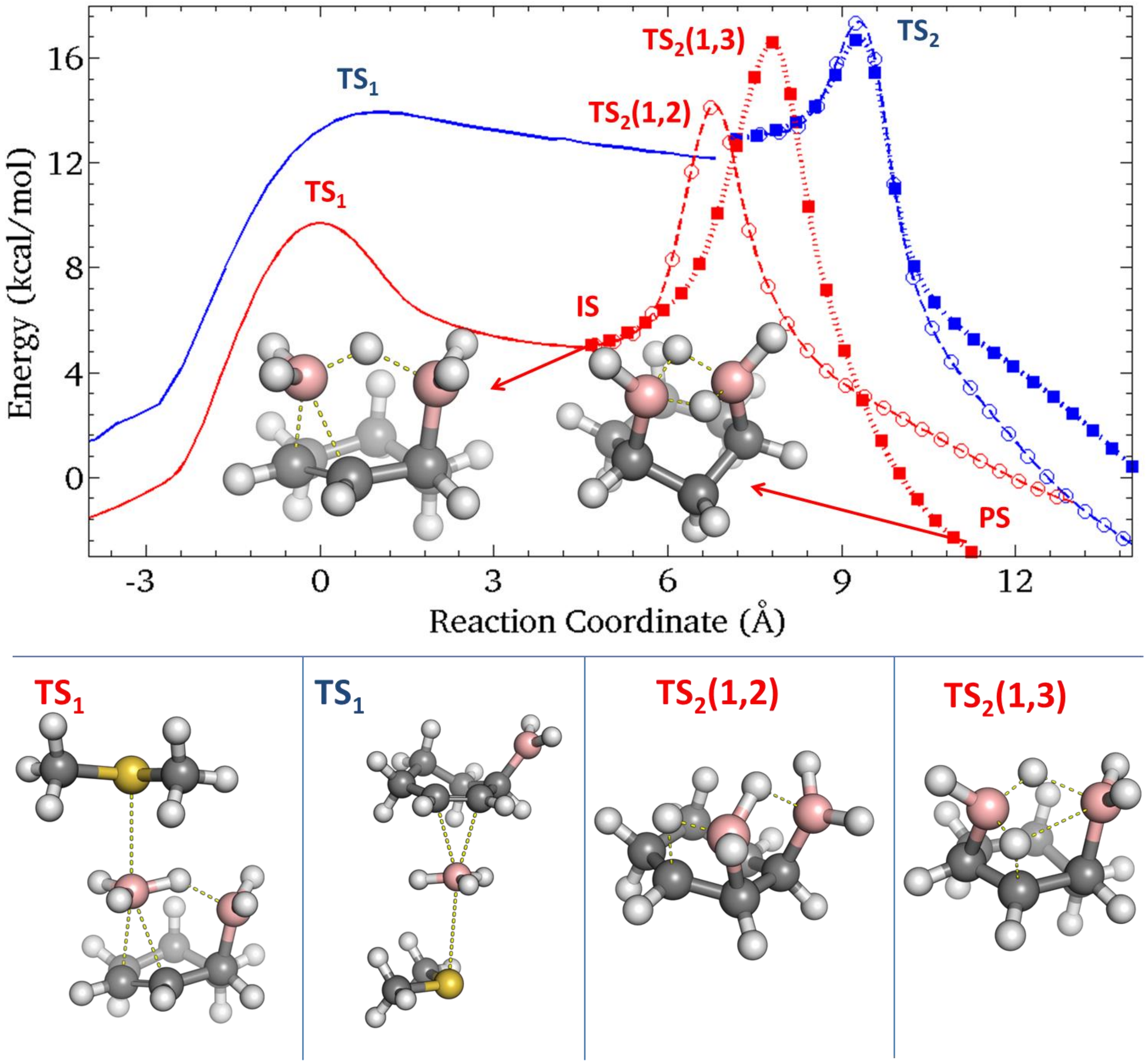

Figure S54: Hydroboration of **13a** to produce 1,2-diborane-cyclohexane (squares) and 1,3-diborane-cyclohexadiene (open circles). The hydroboration could occur via an intermolecular (blue lines) or an intramolecular mechanism stabilized by B-H-B bridges (red lines and inset). Selected transition state geometries are also shown. The reaction paths have been calculated by integrating the intrinsic reaction coordinate.

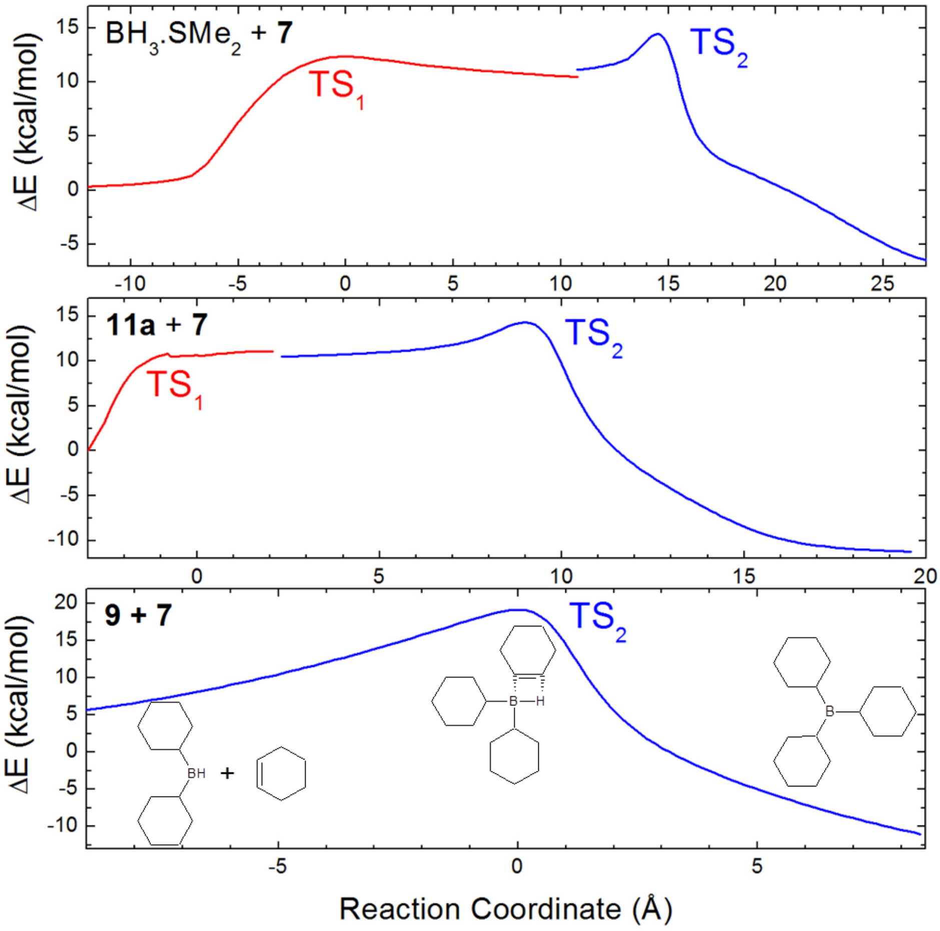

Figure S55: Hydroboration of cyclohexene (**7**) to produce **11a** (top panel), dicyclohexyl-borane (middle panel) and 8 (bottom panel). The reaction paths have been calculated by integrating the intrinsic reaction coordinate and were calculated at the B3LYP/6-31G* level of theory. The energies are referred to the isolated reactants.

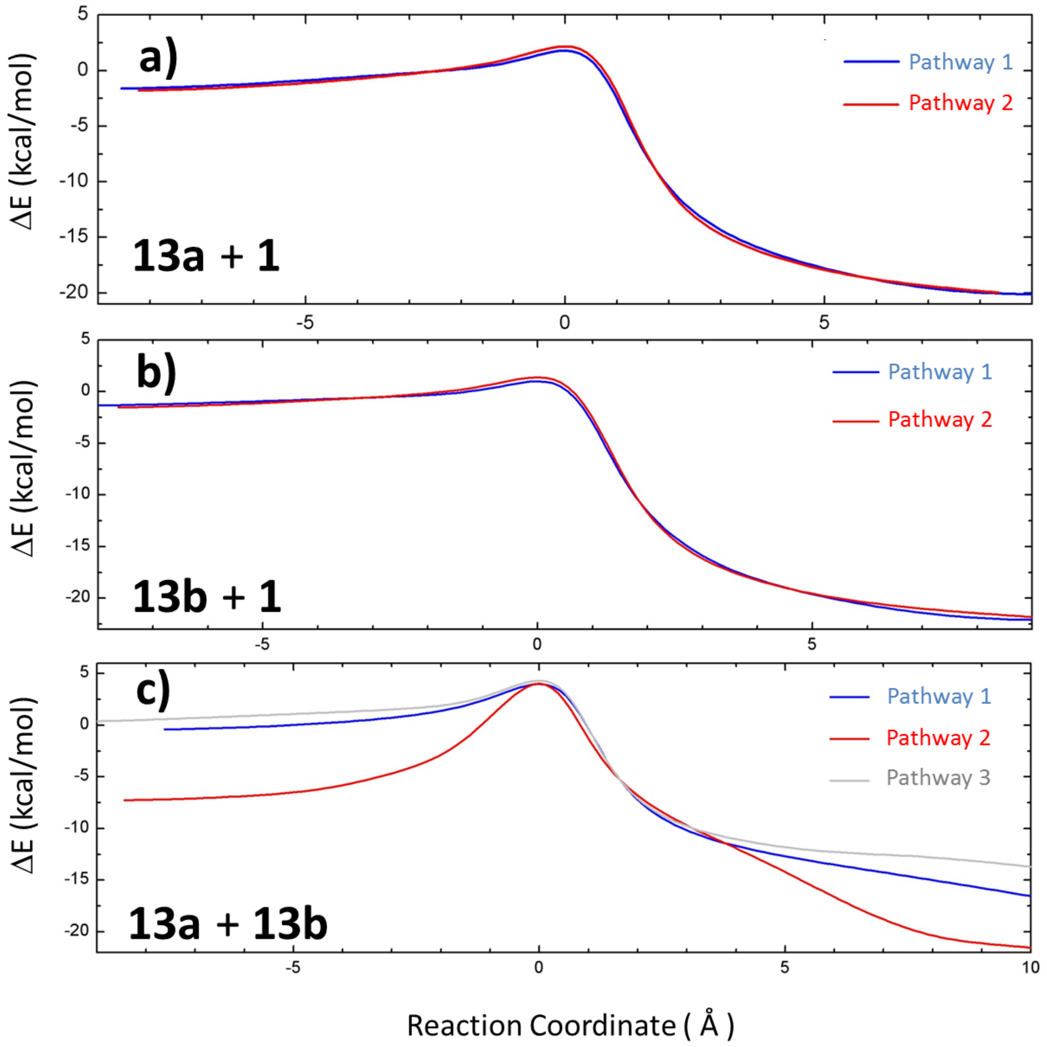

Figure S56: Reaction pathways leading to di-substituted borane products. For each panel at least two different pathways (producing different isomers) are shown. a) Reaction paths for the **13a**+ **1** reaction. Blue (red) line corresponds to transition state S3a (S3b) in Table S3. b) Reaction paths for the **13b**+ **1** reaction. Blue (red) line corresponds to transition state S3c (S3d) in Table S3. c) Reaction paths for the **13a** + **13b** reaction. Blue, red and grey lines correspond to transition states S3e, S3f, and S3g, respectively. The reaction paths have been calculated by integrating the intrinsic reaction coordinate and were calculated at the B3LYP/6-31G* level of theory. The energies are referred to the isolated reactants.

2. **Stabilization of the final polymer via dimers formed by substituted boranes.**

The final product is stabilized by the formation of B-H-B bridged dimers made up of (un)substituted boranes. To model the dimer formation enthalpies of the fully saturated polymer chain, we used our model compounds formed during the hydroboration reaction of cyclohexene. All the results have been calculated at the B3LYP/6-31++G** level of theory. The Grimme D3 correction (S. Grimme, *et. al. J. Chem. Phys.*, **132** (2010), 154104) for the dispersion was used to better described the stabilization energy of the dimers.

a. Ends of the polymer chain are made of up mono-substituted boranes best stabilized as homodimers

	**ΔE_r (kcal/mol)**	**Stabilization energy per free borane (kcal/mol)**
**R=cyclohexyl**		
**$RBH_2 + SMe_2.BH_3 \rightarrow RBH_2.BH_3 + SMe_2$**	-12.7	-12.7
**$2RBH_2 \rightarrow RBH_2.RBH_2$**	-32.4	-16.2
**$RBH_2 + R_2BH \rightarrow RBH_2.R_2BH$**	-25.5	-12.7 (slightly more stable than bound to BH_3 separately, -11.2)
**R=cyclohexenyl**		
**$2RBH_2 \rightarrow RBH_2.RBH_2$**	-29.0	-14.5

[a] RBH_2 corresponds to the 13a isomer. This result is included here to demonstrate that the reactivity of the substituted borane does not significantly depend on the actual R group (i.e., whether or not it is saturated), and the dimer formation reactions can thus take place simultaneously in the same way while the reactions on the double bonds also are in progress.

b. Middle of the chain is made up of di-substituted boranes best stabilized by dimers via RBH_2 and BH_3

**R=cyclohexyl**	**ΔE_r (kcal/mol)**	**Stabilization energy per free borane (kcal/mol)**
**$R_2BH + SMe_2.BH_3 \rightarrow R_2BH.BH_3 + SMe_2$**	-9.8	-9.8
**$RBH_2 + R_2BH \rightarrow RBH_2.R_2BH$**	-25.5	-12.7 (slightly more stable than bound to BH_3 separately, -11.2)
**$2R_2BH \rightarrow R_2BH.R_2BH$[a]**	-15.2	-7.6

c. Tri-substituted boranes

These are not the kinetically favoured products, therefore less of these molecules are expected to be present in the polymeric material.

**R=cyclohexyl**	**ΔE_r (kcal/mol)**
**$R_3B + SMe_2.BH_3 \rightarrow R_3B.BH_3 + SMe_2$**	Not stable
**$R_3B + RBH_2 \rightarrow R_3B.RBH_2$**	Not stable

Alternatively, the information in the previous three tables can be collected based on the number of substituents on the borane (second sub-column for monomers A and B).

**Monomer A**		**Monomer B**		**ΔE_r (kcal/mol)**	**Stabilization energy per free borane (kcal/mol)**
**Molecule**	# of substituents	**Molecule**	# of substituents		
RBH_2	1	$SMe_2.BH_3$	0	-12.7	-12.7
R_2BH	2	$SMe_2.BH_3$	0	-9.8	-9.8
R_3B	3	$SMe_2.BH_3$	0	Not stable	N/A
RBH_2	1	RBH_2	1	-32.4	-16.2
R_2BH	2	RBH_2	1	-25.5	-12.7

R_3B	3	RBH_2	1	Not stable	N/A
R_2BH	2	R_2BH	2	-15.2	-7.6
R_3B	3	R_2BH	2	Not stable	N/A
R_3B	3	R_3B	3	Not stable	N/A

The polymer will thus consist of mainly di-substituted borane chains (…-R-BH(.BH_3)-R-…), which are structurally diverse due to the many possible isomers that form at a similar rate. These boranes are stabilized by B-H-B bridges, mostly via dimers of the left over BH_3, but other substituted boranes also make up the B-H-B bridged dimers. Opposite to the hydroboration reaction on the double bonds, the B-H-B bridge formation is expected to be thermodynamically favoured due to the lack of a significant barrier for these association reactions.

The mono-substituted borane ends of the polymer preferentially dimerize together forming "(1|1) homodimers", whereas the di-substituted boranes are most stable in the RBH2 (2|1) and BH_3-bound (2|0) heterodimeric form. This is consistent with the observed ^{11}B NMR spectra obtained in Figure S16, which shows the heterodimeric form (2|0) of the kinetically favoured di-substituted product with BH_3, which slowly converts into the mono-substituted homodimer (1|1), with other complexes also present in smaller quantities.

3. IR calculation results.

To help assign the IR vibrational spectra, we calculated the vibrational frequencies at the B3LYP/6-311++G(d,p) level of theory on structures optimized at using B3LYP/6-31G(d). The frequencies were scaled using the recommended 0.967 factor (NIST Computational Chemistry Comparison and Benchmark Database, Release 16a, August 2013 http://cccbdb.nist.gov/). The B-H-B bridged dimers present two regions in the IR spectra. The calculated peaks attributed to the B-H-B bonds lie in the 1600-1700 cm^{-1} range. Additional significantly less intense peaks are also present at around 2000-2200 cm^{-1}. The peaks that correspond to the terminal B-H bond stretching frequencies (if present) are in the 2500-2700 cm^{-1} range.

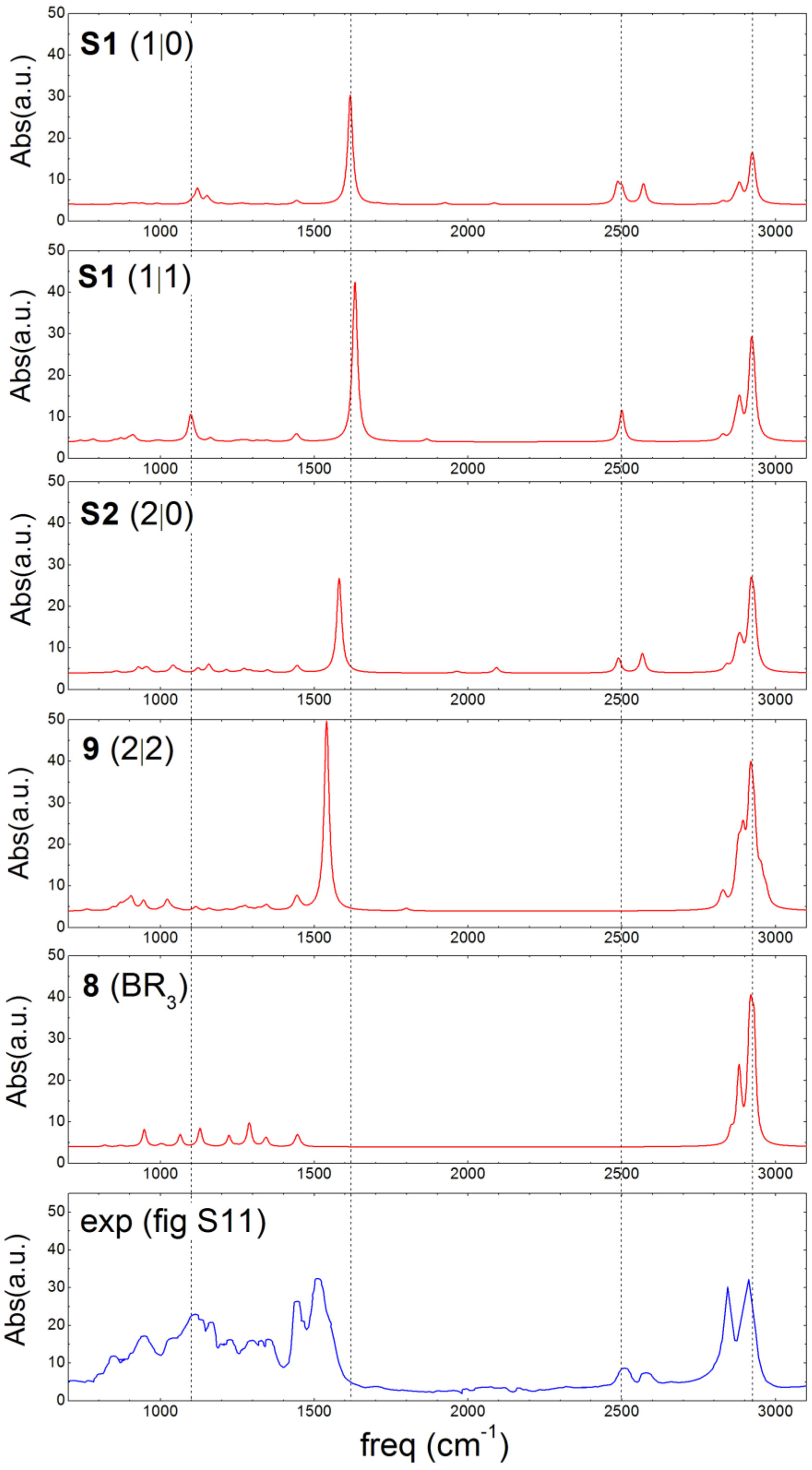

Figure S57: IR spectra of cyclohexyl substituted borane dimers, and compound **8**. Results are shown for molecules **S4** (1|0), **S1** (1|1), **S2** (2|0), **9** (2|2) and **8**. The full width at half maximum was set to 10 cm^{-1} to obtain the broadening of the calculated spectra. Experimental spectrum (blue) also shown for a reference.

Table S4: Main B-H stretching vibrational modes.

**Molecule**	Highest Intensity Peaks	
	Terminal (B-H$_t$) / cm^{-1}	Bridging (B-H$_b$) / cm^{-1}
**S4 (1|0)**	2493 (degenerate), 2570	1616
**S1 (1|1)**	2500	1632
**S2 (2|0)**	2489, 2567	1581
**9 (2|2)**	N/A	1540
**8**	N/A	N/A

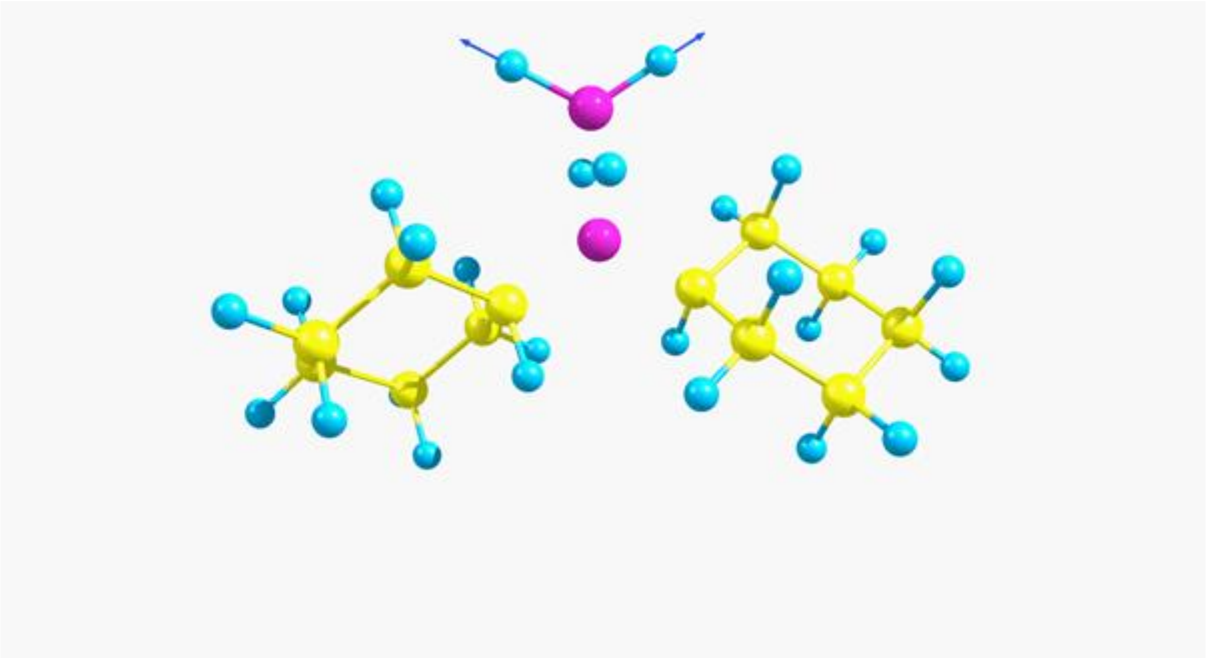

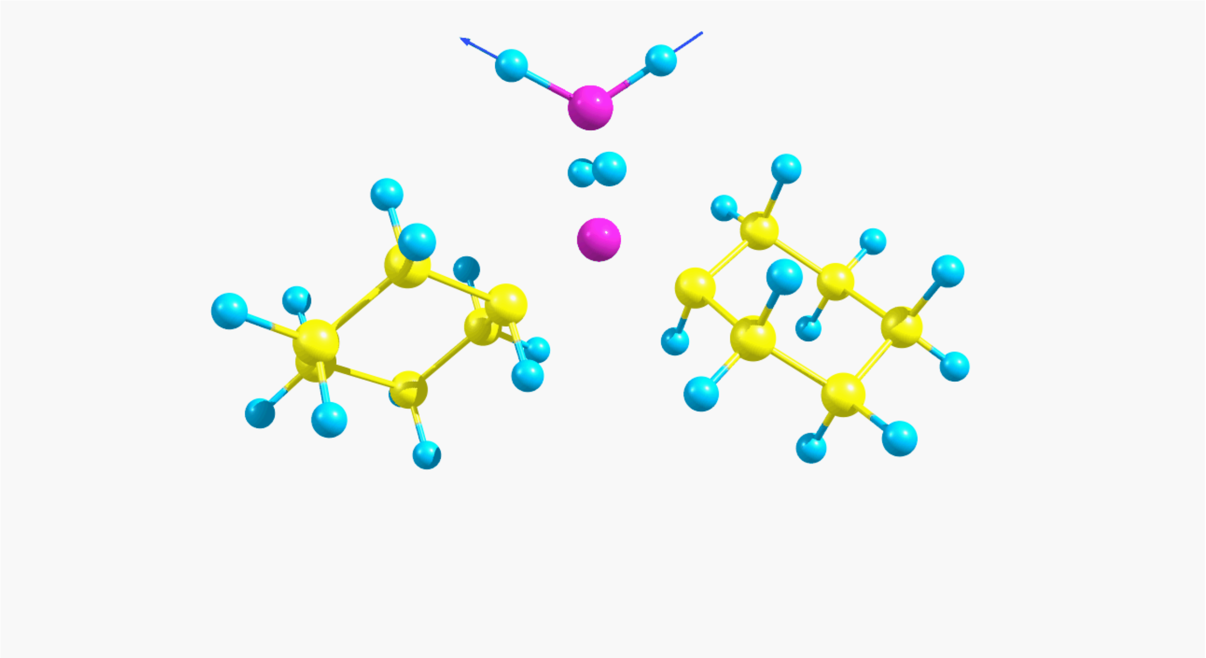

Figure S58: Terminal B-H stretching vibrations corresponding to peaks at 2574.1 cm^{-1} (top) and 2654.6 cm^{-1} (bottom) for **S2** (2|0).

4. NMR calculation results.

Table S5: NMR isotopic shieldings were computed at the B3LYP/6-311+G(d,p) level of theory within the GIAO framework on structures optimized at the B3LYP/6-31G(d) level of theory. $BF_3.O(Et)_2$ was chosen as internal standard to calculate the ^{11}B NMR chemical shift. The method was validated by computing chemical shifts for the compounds nmr1-nmr12 and found a very good agreement with errors of 1.3 ± 1.7 ppm.

	Compound	Experiment	Theory	Difference
**S1**	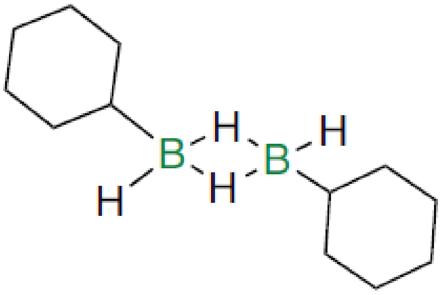	+22	21.5	0.5
**S2**	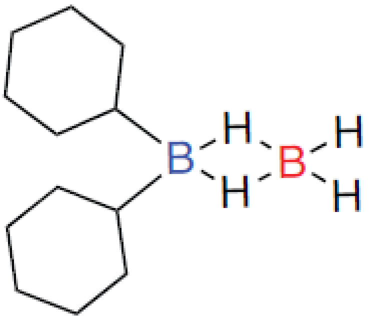	3,40	1.0,42.1	2,2.1
**S3**	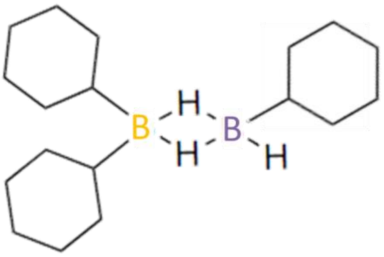	16,34	15.2,34.6	0.8,0.6
**S4**	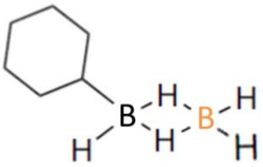	10,30	8.5,30.4	1.5,0.4
**9**	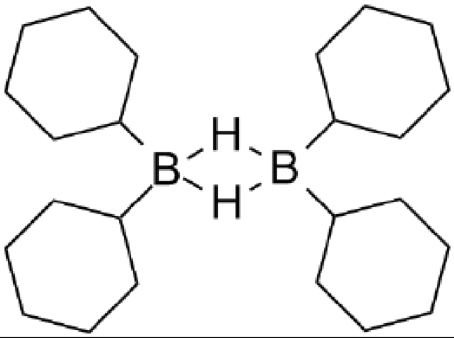	+29	29.6	+0.6

**8**	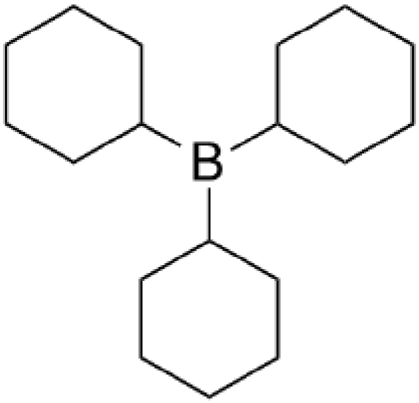	+81	84.2	+3.2
**S5**	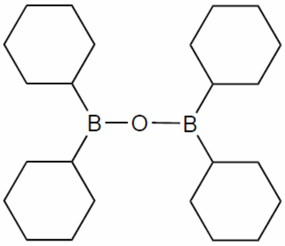		47.1	
**S6**	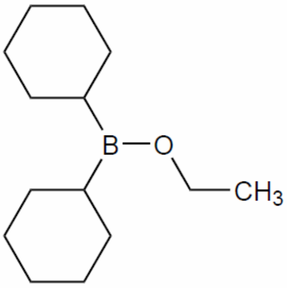		50.2	
**S7**	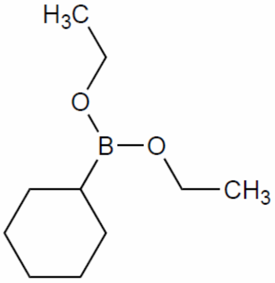		28.0	
**S8**	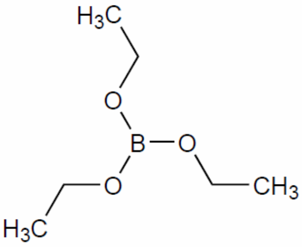		16.0	

**S9**	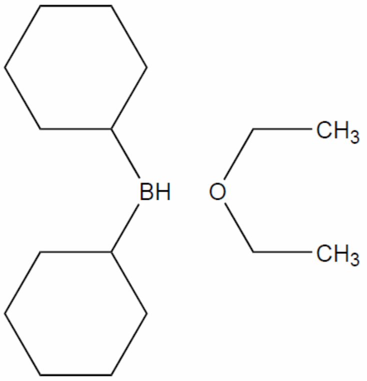		28.9	
**S10**	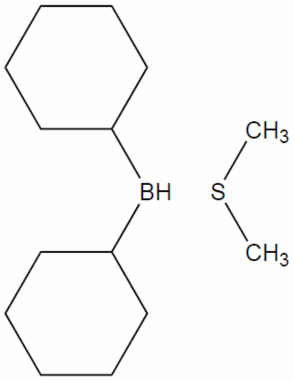		18.4	
**11a.SMe$_2$**	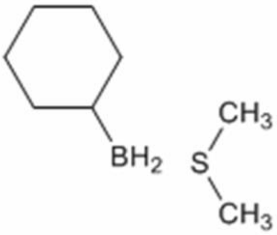		-1.5	
**ref**	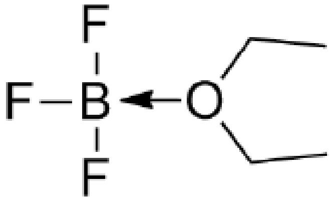	0	0	----
**nmr1**	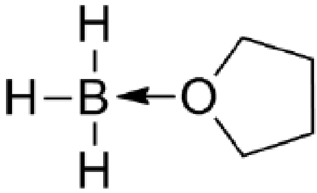	-1	-0.6	0.4
**nmr2**	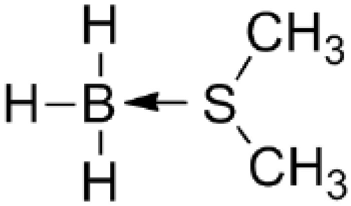	-21	-21.9	-0.9

**nmr3**	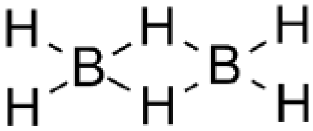	+18	+17.1	-0.8
**nmr4**	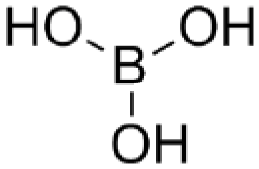	+19	+18.5	-0.5
**nmr5**	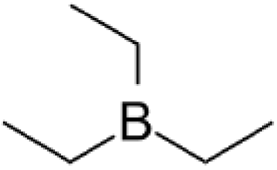	+86	+88.5	1.5
**nmr6**	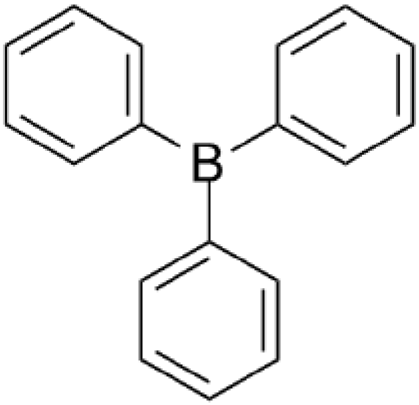	+65	63.0	-2.0
**nmr7**	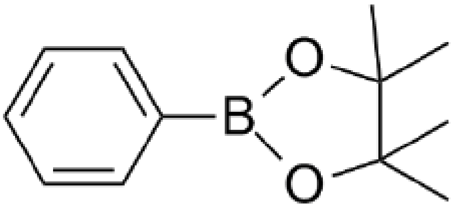	+30	+28.9	-1.1
**nmr8**	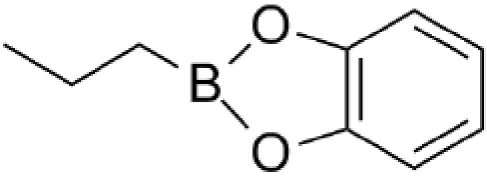	+35	+34.5	-0.5
**nmr9**	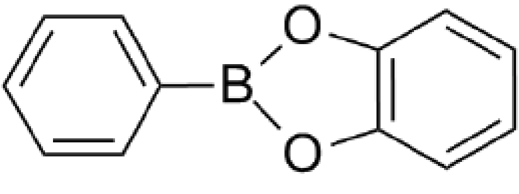	+32	+31.0	-1.0

**nmr10**	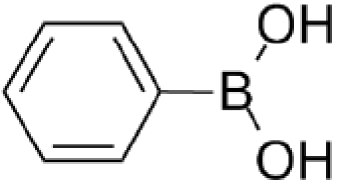	+29	+29.1	0.1
**nmr11**	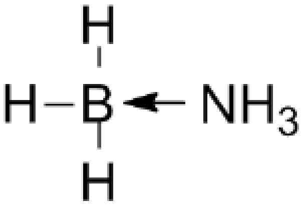	-23	-20.9	+2.1
**nmr12**	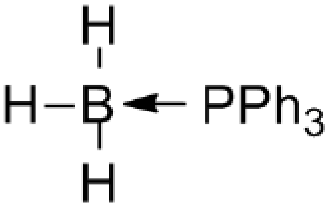	-38	-42.3	-4.3

5. Cartesian coordinates.

Table S5. B3LYP/6-31G(d) optimized geometries (Cartesian coordinates in Å) for the structures shown in Tables S1-S3 Unless otherwise stated, total energies in Hartrees at the B3LYP/6-311++G(d,p) level of theory.

BH_3

Energy= -26.61334 a. u

```
4
B          0.00000        0.00000        0.00000
H          0.00000        1.19400        0.00000
H          1.03400       -0.59700        0.00000
H         -1.03400       -0.59700        0.00000
```

SMe_2

Energy= -478.06685 a. u.

```
9
S          0.00000        0.66390        0.00000
C          1.39440       -0.51560        0.00000
H          1.38120       -1.14640       -0.89450
H          2.31460        0.07450       -0.00020
H          1.38120       -1.14620        0.89460
C         -1.39450       -0.51560        0.00000
H         -1.38110       -1.14640       -0.89450
H         -1.38110       -1.14620        0.89460
H         -2.31460        0.07460       -0.00020
```

BH_3 . SMe_2

Energy= -504.72179 a. u.

```
13

S         -0.02430       -0.00010       -0.53470
C          0.82830       -1.39540        0.26920
C          0.82180        1.39910        0.26910
H          1.87900        1.40920       -0.00750
H          0.33790        2.30950       -0.09060
H          0.69960        1.32790        1.35290
H          1.88550       -1.40090       -0.00750
H          0.70590       -1.32460        1.35300
H          0.34850       -2.30810       -0.09030
B         -1.82610       -0.00390        0.31170
H         -2.32100        1.00950       -0.12190
H         -1.60040       -0.00320        1.50090
H         -2.31690       -1.01940       -0.12160
```

SMe_2

Energy= -478.06685 a. u.

```
9
```

S	0.00000	0.66390	0.00000
C	1.39440	-0.51560	0.00000
H	1.38120	-1.14640	-0.89450
H	2.31460	0.07450	-0.00020
H	1.38120	-1.14620	0.89460
C	-1.39450	-0.51560	0.00000
H	-1.38110	-1.14640	-0.89450
H	-1.38110	-1.14620	0.89460
H	-2.31460	0.07460	-0.00020

7 (cyclohexene)

Energy= -234.71316 a. u.

16

C	0.69820	-1.19240	0.31860
C	-0.69840	-1.19220	-0.31880
H	1.24460	-2.10600	0.05390
C	-1.49890	0.04800	0.11090
H	-1.24490	-2.10580	-0.05440
H	-0.59330	-1.19220	-1.41280
C	-0.66600	1.30610	0.05710
H	-2.38660	0.16390	-0.52640
H	-1.88880	-0.09010	1.13230
C	0.66610	1.30600	-0.05720
H	-1.19940	2.25470	0.11240
C	1.49890	0.04780	-0.11060
H	1.19970	2.25450	-0.11290
H	1.88970	-0.09010	-1.13170
H	2.38630	0.16380	0.52740
H	0.59300	-1.19280	1.41260

$BH3.SMe_2$ + **7** → **11a** (TS_1)

Energy= -739.41293 a. u.

29

C	2.11430	-0.52220	-1.28560
C	1.94360	-1.35130	-0.23390
C	2.39610	-1.01750	1.16850
C	3.33300	0.20200	1.20820
C	2.82480	1.32140	0.28910
C	2.75120	0.84060	-1.16750
B	-0.27240	-0.42110	-0.79040
S	-3.44680	0.24720	-0.51040
C	-3.09440	1.45800	0.80900
C	-3.73110	-1.23970	0.50930

H	1.53080	-2.34370	-0.39980
H	2.89860	-1.89580	1.59630
H	1.51440	-0.84320	1.80050
H	3.43390	0.56250	2.23890
H	4.33770	-0.10140	0.88170
H	1.82300	1.63210	0.61340
H	3.76290	0.78730	-1.60050
H	1.86070	-0.86690	-2.28540
H	-0.12380	0.71190	-1.14470
H	-0.54940	-1.26570	-1.58870
H	-0.44450	-0.63490	0.37590
H	-4.57070	-1.09470	1.19660
H	-3.97250	-2.05690	-0.17550
H	-2.83000	-1.50510	1.07020
H	-3.94620	1.56400	1.48830
H	-2.20080	1.17150	1.37140
H	-2.90970	2.41870	0.32130
H	3.47200	2.20370	0.36090
H	2.19880	1.55970	-1.78420

$BH3.SMe_2$ + **7** → **11a** (IS)

Energy= -261.3476 a. u. (SMe_2 not included)

20

C	0.89860	-0.54220	-0.70660
C	0.80210	0.81120	-0.55460
C	-0.44850	1.49760	-0.03960
C	-1.66240	0.55320	0.02840
C	-1.26810	-0.82820	0.56760
C	-0.22760	-1.48440	-0.34910
B	2.08290	0.02280	0.87390
H	1.57040	1.44750	-0.98470
H	-0.66580	2.34320	-0.70560
H	-0.24620	1.93480	0.94460
H	-2.44640	1.00330	0.64820
H	-2.08810	0.43430	-0.97800
H	-0.84740	-0.72620	1.57600
H	-0.70420	-1.79560	-1.29230
H	1.72370	-0.95730	-1.27840
H	1.77590	-1.02430	1.38140
H	3.15760	0.08490	0.34520
H	1.76160	0.96850	1.54180
H	-2.14950	-1.47460	0.65180
H	0.17890	-2.39530	0.10350

$BH3.SMe_2$ + **7** → **11a** (TS_2)

Energy= -261.342428382 a. u. (SMe_2 not included)

20

C	-0.94573	0.66578	-0.57502

C	-0.94411	-0.75077	-0.45848
C	0.32421	-1.51684	-0.05865
C	1.59412	-0.64613	-0.06940
C	1.34130	0.74047	0.53722
C	0.29465	1.49487	-0.29308
B	-1.89544	-0.08479	0.82187
H	-1.61528	-1.28489	-1.12462
H	0.45599	-2.35339	-0.75611
H	0.19444	-1.96425	0.93249
H	2.40045	-1.16032	0.46721
H	1.94153	-0.51589	-1.10483
H	0.99401	0.64138	1.57305
H	0.72263	1.73488	-1.27847
H	-1.65805	1.11771	-1.26222
H	-1.85237	1.15836	0.72624
H	-3.04949	-0.33975	0.62848
H	-1.33969	-0.31015	1.85747
H	2.26892	1.32461	0.56634
H	0.02746	2.45141	0.17001

BH3.SMe$_2$ + **7** → **11a** (PS, 11a)

Energy= -261.37639 a. u. (SMe$_2$ not included)

20

C	-0.36440	1.27490	-0.22400
C	1.10510	1.27010	0.22660
C	1.83650	0.00120	-0.23670
C	1.10790	-1.26790	0.23050
C	-0.36140	-1.27670	-0.22030
C	-1.11840	-0.00160	0.21420
H	1.61880	2.16420	-0.15020
H	2.87070	0.00310	0.13120
H	1.89660	-0.00050	-1.33550
H	1.62380	-2.16200	-0.14340
H	1.14710	-1.32140	1.32920
H	-0.87370	-2.16710	0.16500
H	-0.87870	2.16510	0.15910
H	-0.39710	-1.35260	-1.31760
B	-2.66140	-0.00100	-0.00420
H	-3.27340	-1.02930	-0.08540
H	-3.27670	1.02860	-0.01040
H	-1.12670	0.00200	1.33340
H	1.14400	1.32710	1.32510
H	-0.40000	1.34760	-1.32150

1 (cyclohexadiene)

Energy(6-311++G**)= -233.48392 a. u.

Energy(6-31G*)= -233.4189363 a. u.

14

C	-0.11380	1.42520	0.06430
C	1.19540	0.73200	-0.23890
H	-0.11620	2.50650	0.18380
C	1.19540	-0.73200	0.23890
H	2.03670	1.27200	0.21110
H	1.36270	0.76430	-1.32930
C	-0.11380	-1.42520	-0.06430
H	2.03670	-1.27200	-0.21110
H	1.36270	-0.76430	1.32930
C	-1.26030	-0.72620	-0.10400
H	-0.11630	-2.50650	-0.18380
C	-1.26030	0.72630	0.10400
H	-2.21110	-1.22740	-0.27110
H	-2.21110	1.22740	0.27110

BH3.SMe_2 + **1** → **13a or 13b** (TS_1)

Energy= -738.18176 a. u.

27

C	-2.13950	-0.08450	-1.33120
C	-1.96530	1.08710	-0.67170
C	-2.38840	1.21490	0.77800
C	-3.58180	0.29980	1.11150
C	-3.43770	-1.06770	0.48490
C	-2.76010	-1.23520	-0.66130
B	0.22800	0.09660	-0.92900
S	3.56010	-0.35620	-0.38920
C	3.10400	-1.20420	1.16160
C	3.69460	1.35600	0.22940
H	-1.58560	1.96220	-1.18960
H	-2.63590	2.25740	1.00740
H	-1.53400	0.95220	1.41750
H	-3.68940	0.21560	2.19920
H	-4.52050	0.75290	0.75110
H	-3.94120	-1.90910	0.95630
H	-2.68470	-2.21090	-1.13330
H	-1.89520	-0.16830	-2.38600
H	0.14450	-1.09650	-0.91010
H	0.47640	0.65720	-1.95510
H	0.40100	0.66390	0.11260
H	4.46500	1.43930	1.00280
H	3.97860	1.98270	-0.62020
H	2.73430	1.70610	0.61970
H	3.88020	-1.08170	1.92390
H	2.14590	-0.83580	1.53970
H	3.00340	-2.26730	0.92790

BH3.SMe_2 + **1** → **13a or 13b** (IS)

Energy= -260.11565 a. u. (SMe_2 not included)

18

C	-0.75810	-0.70730	-0.56560
C	0.40780	-1.39750	0.12770
H	-1.42960	-1.29980	-1.17770
C	1.70740	-0.57860	0.01170
H	0.54820	-2.39710	-0.29730
H	0.16380	-1.53880	1.18670
C	1.46570	0.89220	0.25600
H	2.44850	-0.96660	0.72000
H	2.15530	-0.70250	-0.98790
C	0.28490	1.46090	-0.02710
H	2.28840	1.49530	0.63440
C	-0.79610	0.65890	-0.62330
H	0.11450	2.52240	0.12710
H	-1.52170	1.16580	-1.25190
B	-2.26390	-0.03150	0.67770
H	-3.22560	-0.05200	-0.03870
H	-2.03420	-1.01990	1.32300
H	-2.05740	0.97920	1.29760

BH3.SMe_2 + **1** → **13a** (TS_2)

Energy= -260.11217 a. u. (SMe_2 not included)

18

C	-0.79500	-0.75450	-0.50080
C	0.41000	-1.37910	0.19790
H	-1.30080	-1.33950	-1.26330
C	1.68420	-0.55380	-0.06480
H	0.53360	-2.41800	-0.12640
H	0.22870	-1.39590	1.27760
C	1.43700	0.91610	0.19140
H	2.49050	-0.92670	0.57770
H	2.03210	-0.70000	-1.10010
C	0.22950	1.45940	-0.00370
H	2.26800	1.52710	0.53730
C	-0.89950	0.65390	-0.53660
H	0.05150	2.51540	0.18400
H	-1.46680	1.10430	-1.34700
B	-2.07280	0.03220	0.61100
H	-3.16010	0.25730	0.16270
H	-2.00160	-1.20790	0.64230
H	-1.70890	0.37150	1.70020

BH3.SMe_2 + **1** → **13a** (PS, 13a)

Energy(6-311++G**)= -260.14727 a. u. (SMe_2 not included)

Energy (6-31G*)= -260.078337 a. u. (SMe_2 not included)

18

C	-0.65910	1.13680	-0.40150
C	0.61050	1.42770	-0.06660
C	1.67050	0.39230	0.20110
C	1.10430	-1.02800	0.37010
C	-0.05220	-1.30840	-0.60290
C	-1.19500	-0.28850	-0.45260
H	0.92880	2.47010	-0.05460
H	2.25440	0.67640	1.08760
H	2.38740	0.42160	-0.63560
H	1.90400	-1.76490	0.22880
H	0.74510	-1.15030	1.39800
H	-0.43180	-2.32540	-0.44370
H	-1.90980	-0.36400	-1.28150
H	-1.35190	1.94530	-0.62510
H	0.32810	-1.27240	-1.63430
B	-1.88760	-0.12040	0.94050
H	-1.38370	-0.53020	1.94520
H	-2.90620	0.50340	1.02740

13a.SMe_2

Energy= -738.23498 a. u.

27

C	1.14400	0.78150	1.03200
C	2.06590	1.38210	0.26570
C	2.90690	0.63840	-0.74530
C	2.86920	-0.87700	-0.49220
C	1.42680	-1.35730	-0.27650
C	0.74680	-0.67160	0.92940
H	2.26260	2.44710	0.39280
H	3.94230	1.00500	-0.70350
H	2.55980	0.85800	-1.76830
H	3.33710	-1.41310	-1.32820
H	3.46410	-1.10390	0.40360
H	1.39680	-2.44560	-0.14260
H	0.62280	1.37100	1.78870
H	0.85420	-1.14920	-1.19360
B	-0.84950	-0.93030	1.03490
H	-1.40490	-0.41770	1.98430
H	-1.17890	-2.08220	0.84650
H	1.11420	-1.16630	1.84950
S	-1.82150	-0.06490	-0.54650
C	-3.52800	-0.61860	-0.23310
H	-4.21580	-0.10150	-0.90670
H	-3.55570	-1.69210	-0.42880
H	-3.79020	-0.43390	0.81170
C	-1.97450	1.70290	-0.12850
H	-2.33330	1.80820	0.89860
H	-0.97310	2.12910	-0.21090
H	-2.65200	2.19370	-0.83180

BH3.SMe_2 + **1** → **13b** (TS_2)

Energy= -260.11217 a. u. (SMe_2 not included)

18

C	-0.85830	-0.69560	-0.47760
C	0.35190	-1.40460	0.14670
H	-1.34460	-1.20840	-1.30190
C	1.66040	-0.61960	-0.06260
H	0.44520	-2.40740	-0.28390
H	0.19520	-1.53820	1.22120
C	1.47470	0.85270	0.21150
H	2.44650	-1.02500	0.58610
H	2.03140	-0.74290	-1.09280
C	0.29940	1.47090	0.03100
H	2.33900	1.42700	0.54090
C	-0.84300	0.72080	-0.52980
H	0.18180	2.53460	0.22050
H	-1.42890	1.22660	-1.29410
B	-2.08450	-0.11400	0.60040
H	-3.16190	-0.34630	0.12840
H	-1.75530	-0.43150	1.70780
H	-2.03610	1.13340	0.64990

BH3.SMe_2 + **1** → **13b** (PS, 13b)

Energy(6-311++G**)= -260.14364 a. u.

Energy(6-31G*)= -260.0738871 a. u.

18

C	1.02090	-0.15580	0.36720
C	0.16520	-1.26500	-0.27380
H	0.88720	-0.18660	1.45940
C	-1.31240	-1.15090	0.12930
H	0.55180	-2.25420	0.00180
H	0.25050	-1.19250	-1.36690
C	-1.81440	0.26720	0.02280
H	-1.92840	-1.80930	-0.49910
H	-1.45350	-1.51500	1.15970
C	-1.00820	1.32610	-0.09380
H	-2.89340	0.41720	0.03680
C	0.50050	1.24870	-0.09010
H	-1.43670	2.32290	-0.19670
H	0.90690	2.02570	0.57050
B	2.53650	-0.14450	-0.01330
H	3.33210	0.44740	0.66020
H	2.92150	-0.65380	-1.02920
H	0.86980	1.49950	-1.09960

13b.SMe_2

Energy= -738.23286 a. u.

27

C	-0.68960	0.30620	0.09090
C	-1.23610	-0.83750	-0.78760

H	-0.60430	-0.08650	1.12100
C	-2.54530	-1.42320	-0.23270
H	-0.49300	-1.63960	-0.89870
H	-1.41610	-0.44630	-1.79910
C	-3.50940	-0.34310	0.19290
H	-3.02230	-2.07020	-0.98350
H	-2.33110	-2.08330	0.62450
C	-3.15160	0.93540	0.34890
H	-4.54340	-0.63940	0.37010
C	-1.74170	1.43970	0.15530
H	-3.90530	1.67130	0.63150
H	-1.48530	2.13610	0.96600
B	0.71290	0.93830	-0.40070
H	1.14580	1.84900	0.27640
H	0.79360	1.14370	-1.59430
H	-1.69860	2.04560	-0.76550
S	2.18760	-0.45570	-0.19010
C	3.65080	0.47850	-0.73940
H	3.54910	0.61990	-1.81720
H	3.67090	1.45370	-0.24650
H	4.55930	-0.08950	-0.52490
C	2.49160	-0.50580	1.60650
H	2.58130	0.51320	1.99170
H	1.62780	-0.99190	2.06490
H	3.39260	-1.08640	1.81890

$BH3.SMe_2$ + **13a** → 1-2,diboro-cyclohexane or 1-3,diboro-cyclohexane (TS_1 intramolecular)

Energy= -764.85147 a. u.

31

C	3.14480	0.87050	-0.58920
C	2.76870	-0.45320	-1.27210
H	4.13980	0.76330	-0.13100
C	2.65280	-1.60140	-0.25450
H	3.50890	-0.71240	-2.03880
H	1.81050	-0.33230	-1.79190
C	1.95700	-1.18370	1.01890
H	2.13400	-2.45920	-0.70120
H	3.65550	-1.96900	0.01600
C	1.79120	0.10720	1.38640
H	1.66960	-1.97360	1.71150
C	2.11780	1.27080	0.49090
H	1.35810	0.33010	2.35960
H	2.53840	2.08030	1.10770
B	0.69490	1.80440	-0.04250
H	-0.03580	2.32600	0.75400
H	3.22560	1.66710	-1.33820
H	0.57730	2.12930	-1.19510
B	-0.33680	-0.49520	0.35500
H	-0.10230	0.51440	-0.31130
H	-0.23840	-1.50490	-0.26620

H	-0.86960	-0.40110	1.41190
S	-2.92440	-0.08930	-0.72110
C	-3.78730	-1.36530	0.25690
H	-3.60480	-2.32520	-0.23280
H	-3.39280	-1.40620	1.27670
H	-4.86490	-1.17760	0.28010
C	-3.35130	1.39330	0.25420
H	-2.97790	1.30370	1.27870
H	-2.85990	2.24360	-0.22430
H	-4.43300	1.55810	0.26060

$BH3.SMe_2$ + **13a** → 1-2,diboro-cyclohexane or 1-3,diboro-cyclohexane (TS_1 intermolecular)

Energy= -764.84573 a. u.

31

C	-1.89730	-0.42130	-1.11940
C	-1.61630	0.89990	-1.14830
C	-1.99510	1.85460	-0.03980
C	-2.97930	1.22940	0.96340
C	-2.58490	-0.21570	1.30220
C	-2.60970	-1.08830	0.03140
B	0.50420	-0.25170	-0.75060
S	3.67640	-0.72010	-0.09330
C	3.44230	-0.22200	1.64690
C	4.11550	0.89740	-0.81570
H	-1.17300	1.32750	-2.04440
H	-2.43070	2.75970	-0.48450
H	-1.08490	2.18700	0.47800
H	-3.02480	1.84090	1.87220
H	-3.99140	1.23230	0.53300
H	-3.26260	-0.62990	2.05740
H	-2.06720	-2.03880	0.24560
H	-1.69560	-1.03200	-1.99580
H	0.28080	-1.18910	-0.04110
H	0.76610	-0.42620	-1.90350
H	0.77650	0.79710	-0.23930
H	5.02650	1.30020	-0.36160
H	4.29420	0.73450	-1.88180
H	3.29410	1.61090	-0.70160
H	4.36120	0.20060	2.06570
H	2.62380	0.49820	1.73980
H	3.18190	-1.12420	2.20660
H	-1.57530	-0.21980	1.73380
B	-3.98030	-1.72510	-0.38020
H	-4.85590	-1.86280	0.42560
H	-4.13680	-2.16600	-1.48230

$BH3.SMe_2$ + **13a** → 1-2,diboro-cyclohexane or 1-3,diboro-cyclohexane (IS intramolecular)

Energy= -286.79131 a. u. (SMe_2 not included)

22

C	0.85430	-1.45200	-0.26270
C	1.52380	-0.42120	0.65960
H	1.43470	-1.53250	-1.19440
C	1.47940	0.99300	0.05670
H	2.56570	-0.70170	0.85350
H	1.01390	-0.41830	1.63030
C	0.12360	1.33720	-0.53270
H	1.76570	1.74340	0.80160
H	2.21110	1.07250	-0.76140
C	-0.73130	0.35690	-0.98870
H	-0.00980	2.35000	-0.91030
C	-0.60330	-1.06920	-0.58610
H	-1.57700	0.63970	-1.61080
H	-1.01200	-1.71310	-1.37320
B	-1.63680	-1.07220	0.67510
H	-2.80510	-1.10300	0.41190
H	0.87410	-2.44100	0.20880
H	-1.26620	-1.62790	1.67640
B	-1.29100	1.32560	0.72560
H	-1.54740	0.22710	1.29850
H	-0.61000	1.89000	1.53710
H	-2.27770	1.87940	0.35170

BH3.SMe_2 + **13a** → 1-2,diboro-cyclohexane or 1-3,diboro-cyclohexane (IS intermolecular)

Energy= -286.78003 a. u. (SMe_2 not included)

22

C	-0.94930	0.67680	0.96810
C	-0.39980	1.73180	-0.00260
H	-0.28990	0.61000	1.84290
C	1.02360	1.37910	-0.47070
H	-0.39970	2.72360	0.46370
H	-1.06840	1.79680	-0.87360
C	1.19920	-0.09850	-0.76400
H	1.26840	1.93710	-1.38420
H	1.76000	1.69160	0.27830
C	0.26400	-1.03730	-0.43830
H	2.01340	-0.37530	-1.42780
C	-1.02450	-0.69350	0.26720
H	0.34500	-2.03780	-0.85300
H	-1.20220	-1.47830	1.03910
B	-2.29830	-0.99500	-0.59230
H	-2.28610	-1.85860	-1.42130
H	-1.94190	0.96840	1.32880
H	-3.32850	-0.43290	-0.35670
B	1.93700	-1.26120	0.79930
H	2.61090	-0.30410	1.06950
H	1.15390	-1.54800	1.66640
H	2.49220	-2.16200	0.23460

$BH3.SMe_2$ + **13a** → 1-2,diboro-cyclohexane (TS_2 intramolecular)

Energy= -286.77757 a. u. (SMe_2 not included)

22

C	0.86580	-1.39050	-0.41610
C	1.55810	-0.47120	0.60040
H	1.39270	-1.31360	-1.38030
C	1.49630	0.99320	0.14730
H	2.60810	-0.75850	0.73280
H	1.07750	-0.57790	1.58060
C	0.11870	1.41670	-0.33880
H	1.86510	1.67280	0.92460
H	2.15370	1.12990	-0.72440
C	-0.83920	0.47050	-0.84140
H	0.07220	2.41580	-0.77080
C	-0.62330	-1.03340	-0.58840
H	-1.41550	0.77300	-1.71040
H	-1.02740	-1.59190	-1.44120
B	-1.55140	-1.23790	0.70000
H	-2.73670	-1.28250	0.53210
H	0.95690	-2.43400	-0.08980
H	-1.11800	-1.66450	1.73730
B	-1.44590	1.16180	0.56880
H	-1.48250	0.30810	1.44430
H	-0.47630	1.85610	1.00440
H	-2.34210	1.93640	0.43800

$BH3.SMe_2$ + **13a** → 1-2,diboro-cyclohexane (TS_2 intermolecular)

Energy= -286.77307 a. u. (SMe_2 not included)

22

C	-0.91510	0.86320	0.86050
C	-0.15200	1.75120	-0.12980
H	-0.35360	0.81620	1.80280
C	1.27160	1.21760	-0.35940
H	-0.09520	2.78330	0.23620
H	-0.69680	1.78500	-1.08440
C	1.28930	-0.28610	-0.64880
H	1.74600	1.73560	-1.20130
H	1.88290	1.43740	0.52230
C	0.19320	-1.13240	-0.30120
H	1.91410	-0.60050	-1.48150
C	-1.10240	-0.54960	0.27020
H	0.04650	-1.99520	-0.94560
H	-1.43780	-1.21700	1.10270
B	-2.37190	-0.77450	-0.60780
H	-2.42420	-1.67570	-1.39600
H	-1.89280	1.30120	1.09100
H	-3.35910	-0.12160	-0.41840
B	1.48870	-1.44900	0.76760
H	2.45280	-0.74110	0.40260
H	1.22910	-1.01670	1.85520
H	1.89680	-2.55670	0.56610

BH3.SMe_2 + **13a** → 1-2,diboro-cyclohexane (PS intramolecular)

Energy= -286.77757 a. u. (SMe_2 not included)

22

C	-0.77630	-1.46770	0.29910
C	-1.72040	-0.59520	-0.54120
H	-1.25310	-1.61480	1.27960
C	-1.77290	0.83480	0.01240
H	-2.72170	-1.04430	-0.55060
H	-1.38380	-0.56270	-1.58870
C	-0.39370	1.49730	-0.10490
H	-2.51660	1.43120	-0.53210
H	-2.09560	0.80930	1.06400
C	0.73220	0.72340	0.62990
H	-0.42060	2.52880	0.26770
C	0.61880	-0.85310	0.51470
H	0.78190	1.06570	1.67140
H	1.02210	-1.27920	1.44600
B	1.81930	-0.94470	-0.53380
H	2.81490	-0.32090	0.12480
H	-0.68280	-2.46740	-0.14630
H	2.31690	-1.81370	-1.18520
B	2.09130	0.76550	-0.20620
H	1.74440	0.13550	-1.34180
H	-0.16560	1.57730	-1.17930
H	2.88100	1.61500	-0.48960

BH3.SMe_2 + **13a** → 1-2,diboro-cyclohexane (PS intermolecular)

Energy= -286.77307 a. u. (SMe_2 not included)

22

C	1.73120	-0.87340	-0.32320
C	1.92590	0.51890	0.29060
H	1.78000	-0.80060	-1.41930
C	0.75820	1.45430	-0.05730
H	2.87510	0.95170	-0.05030
H	2.00070	0.42370	1.38410
C	-0.60040	0.86150	0.34240
H	0.89830	2.42520	0.43630
H	0.76030	1.65620	-1.13850
C	-0.82090	-0.57380	-0.30810
H	-0.63990	0.70220	1.42990
C	0.38380	-1.49300	0.08020
H	0.26960	-2.47510	-0.39810
H	2.54530	-1.54340	-0.01720
B	-1.90070	1.50610	-0.20310
H	-1.92630	2.14760	-1.21490
H	-0.79860	-0.47290	-1.40360
H	-2.94850	1.22770	0.32110
H	0.36820	-1.66330	1.16550
B	-2.13860	-1.26810	0.19180
H	-2.34410	-1.34130	1.37030
H	-2.91090	-1.79440	-0.55680

$BH3.SMe_2$ + **13a** → 1-3,diboro-cyclohexane (TS_2 intramolecular)

Energy= -286.77356 a. u. (SMe_2 not included)

22

C	1.10090	-1.19000	-0.46650
C	1.64640	-0.18080	0.55190
H	1.55740	-1.00710	-1.45150
C	1.22850	1.25620	0.20420
H	2.74110	-0.23690	0.59500
H	1.28120	-0.44650	1.54940
C	-0.21240	1.38860	-0.29520
H	1.38580	1.91950	1.06280
H	1.88100	1.63320	-0.59710
C	-0.86480	0.28710	-0.96490
H	-0.41310	2.35970	-0.75000
C	-0.44340	-1.11460	-0.56070
H	-1.40380	0.45410	-1.89620
H	-0.80680	-1.83260	-1.30530
B	-1.19770	-1.38160	0.85650
H	-2.35230	-1.69830	0.79310
H	1.38480	-2.20760	-0.17320
H	-0.57550	-1.78050	1.80300
B	-1.52850	0.94980	0.60960
H	-1.27890	0.04840	1.44280
H	-2.19840	1.86110	1.00050
H	-2.30310	0.41310	-0.21550

$BH3.SMe_2$ + **13a** → 1-3,diboro-cyclohexane (TS_2 intermolecular)

Energy= -286.77401 a. u. (SMe_2 not included)

22

C	-0.94620	0.75050	0.93470
C	-0.32880	1.70740	-0.09430
H	-0.32760	0.72890	1.84020
C	1.10330	1.29230	-0.47900
H	-0.32560	2.73460	0.28930
H	-0.96690	1.71300	-0.99170
C	1.28000	-0.22270	-0.65390
H	1.37970	1.78510	-1.41940
H	1.80440	1.66320	0.27610
C	0.25570	-1.13810	-0.27210
H	1.88100	-0.51830	-1.50910
C	-1.05680	-0.66050	0.32170
H	0.20730	-2.08670	-0.80190
H	-1.37600	-1.37260	1.11670
B	-2.26800	-0.88970	-0.64730
H	-2.23340	-1.73150	-1.49800
H	-1.94270	1.09700	1.23120
H	-3.28550	-0.28480	-0.47260
B	1.83000	-1.10970	0.71250
H	1.93120	-0.36340	1.64290
H	0.90130	-1.87720	1.04770
H	2.69960	-1.86350	0.38030

BH3.SMe_2 + **13a** → 1-3,diboro-cyclohexane (PS intramolecular)

Energy= -286.83685 a. u. (SMe_2 not included)

22

C	1.07430	1.29390	0.00860
C	1.58010	0.00010	-0.67070
H	1.66800	1.48040	0.91520
C	1.07450	-1.29380	0.00840
H	2.67710	0.00020	-0.68860
H	1.27730	0.00020	-1.72600
C	-0.42110	-1.21700	0.42720
H	1.25400	-2.14850	-0.65710
H	1.66820	-1.48030	0.91500
C	-0.58270	-0.00010	1.36740
H	-0.69710	-2.15760	0.92060
C	-0.42140	1.21690	0.42720
H	0.15790	-0.00000	2.18080
H	-0.69740	2.15730	0.92090
B	-1.38080	0.88660	-0.81630
H	-2.31870	0.00020	-0.45800
H	1.25390	2.14860	-0.65690
H	-1.91320	1.66590	-1.54740
B	-1.38080	-0.88690	-0.81650
H	-0.85200	0.00020	-1.64770
H	-1.91420	-1.66460	-1.54850
H	-1.57770	-0.00040	1.83300

BH3.SMe_2 + **13a** → 1-3,diboro-cyclohexane (PS intermolecular)

Energy= -286.80678 a. u. (SMe_2 not included)

22

C	-1.26810	1.04620	0.27590
C	-0.03560	1.82210	-0.20970
H	-1.28400	1.05400	1.37620
C	1.26870	1.10590	0.16860
H	-0.04260	2.83890	0.20440
H	-0.08560	1.93060	-1.30380
C	1.31480	-0.34420	-0.35510
H	2.12850	1.67100	-0.21450
H	1.37410	1.09420	1.26380
C	0.05810	-1.13620	0.14370
H	1.24620	-0.32980	-1.45490
C	-1.26640	-0.41960	-0.21210
H	0.05050	-2.14960	-0.27580
H	-2.18950	1.54560	-0.04800
B	2.53110	-1.20710	0.11290
H	3.07040	-0.99440	1.16360
H	0.10840	-1.25250	1.23690
H	2.89570	-2.15270	-0.52770
B	-2.57060	-1.23420	0.05070
H	-3.63000	-0.67670	0.11930
H	-2.55250	-2.43190	0.10250
H	-1.32160	-0.38570	-1.32800

11a.SMe_2

Energy= - 739.46278 a. u.

29

C	-0.65820	-0.45920	-0.21250
C	-1.02170	1.01520	0.06920
H	-0.67880	-0.59480	-1.30880
C	-2.43480	1.39250	-0.40790
H	-0.94500	1.20500	1.15170
H	-0.29550	1.68750	-0.41390
C	-3.49800	0.45570	0.18340
H	-2.66020	2.43630	-0.14860
H	-2.47200	1.32940	-1.50600
C	-3.16890	-1.01370	-0.11600
H	-3.54000	0.60050	1.27370
H	-4.49260	0.71410	-0.20410
C	-1.75720	-1.38390	0.36740
H	-3.91580	-1.67190	0.34880
H	-3.23570	-1.18350	-1.20140
H	-1.73120	-1.32700	1.46690
H	-1.53650	-2.42830	0.11120
B	0.76390	-0.95500	0.36690
H	0.96780	-0.75540	1.54750
H	1.08660	-2.06780	0.00640
S	2.30100	0.09740	-0.50040
C	3.72330	-0.95320	-0.06150
H	3.67480	-1.21380	0.99890
H	3.64470	-1.86190	-0.66060
H	4.65620	-0.43380	-0.29460
C	2.62080	1.51330	0.60430
H	2.64330	1.16920	1.64130
H	3.56380	1.99300	0.32990
H	1.79940	2.22000	0.47480

11a +7 → dicyclohexyl-borane (TS_1)

Energy= -974.15826 a. u.

45

C	-0.97010	1.80600	1.14330
C	-0.74620	3.32450	1.07340
C	-0.96500	3.86390	-0.34830
C	-0.08870	3.12320	-1.36960
C	-0.30980	1.60350	-1.30920

C	-0.10740	1.03540	0.11550
H	-1.41020	3.83910	1.78080
H	-0.76080	4.94210	-0.38240
H	-2.02290	3.73740	-0.62250
H	-0.28930	3.49570	-2.38300
H	0.97000	3.34120	-1.16070
H	0.36020	1.09380	-2.01380
H	-0.76280	1.43790	2.15640
H	-1.33430	1.37660	-1.64020
B	-0.21160	-0.52580	0.22400
H	-0.50410	-1.05040	1.26330
H	-0.02980	-1.22880	-0.73100
H	0.95460	1.21970	0.38740
S	-4.28340	-1.22050	-0.33810
C	-3.56670	-2.67970	-1.17210
H	-4.17850	-3.57130	-1.00160
H	-3.55250	-2.46270	-2.24340
H	-2.54170	-2.86440	-0.83650
C	-4.22830	-1.80280	1.39250
H	-3.19980	-1.99730	1.71110
H	-4.64480	-1.00520	2.01350
H	-4.83410	-2.70480	1.52470
C	2.86380	-0.92650	1.41230
C	2.59670	-2.04990	0.73220
C	3.27030	-2.41780	-0.56770
C	4.50960	-1.55030	-0.84260
C	4.22580	-0.07320	-0.53770
C	3.84310	0.11990	0.93860
H	2.36540	-0.74570	2.36410
H	1.86870	-2.75500	1.13040
H	3.54870	-3.48060	-0.54860
H	5.33920	-1.89430	-0.20920
H	5.09460	0.54800	-0.78720
H	3.41820	1.12210	1.09240
H	3.39760	0.27150	-1.17240
H	4.74460	0.08610	1.57130
H	-2.03130	1.59100	0.94800
H	0.28350	3.55150	1.39020
H	2.54670	-2.31900	-1.39170
H	4.83460	-1.67490	-1.88270

11a +7 → dicyclohexyl-borane (IS)

Energy= -974.15865 a. u.

45

C	0.44670	1.72930	-0.42680
C	0.43300	2.34820	0.98670
H	1.18740	2.27530	-1.03430
C	0.02190	3.82960	0.98460
H	-0.26510	1.77930	1.61940
H	1.42210	2.25020	1.45570
C	-1.33580	4.03670	0.29930
H	-0.00760	4.21630	2.01200
H	0.78690	4.41550	0.45280
C	-1.33960	3.44370	-1.11680
H	-2.12050	3.54860	0.89650

H	-1.58820	5.10470	0.26530
C	-0.93550	1.95880	-1.11130
H	-2.33110	3.55820	-1.57490
H	-0.63510	4.00630	-1.74740
H	-1.70800	1.38340	-0.57930
H	-0.91650	1.57640	-2.14030
B	0.69360	0.16640	-0.50510
H	0.12890	-0.54830	0.28010
H	1.04560	-0.33160	-1.53770
C	2.96460	-2.77990	0.02170
C	4.23040	-2.15070	-0.57620
H	3.13780	-3.83390	0.26980
C	3.98070	-0.69050	-0.99040
H	4.58000	-2.73020	-1.43880
H	5.03530	-2.18170	0.17160
C	3.20570	0.07370	0.05650
H	4.93550	-0.17800	-1.17020
H	3.43760	-0.65150	-1.94520
C	2.56020	-0.51900	1.08120
H	3.24150	1.16000	0.00670
C	2.50970	-2.01410	1.27400
H	2.10790	0.09460	1.85700
H	3.15140	-2.27280	2.13140
H	1.49400	-2.31300	1.56130
H	2.16120	-2.75570	-0.72560
S	-3.77030	-1.71150	0.47430
C	-2.63280	-2.52110	-0.70260
H	-2.94010	-2.33900	-1.73740
H	-1.64450	-2.08100	-0.54640
H	-2.57580	-3.59930	-0.52110
C	-5.32630	-2.55400	0.02300
H	-5.25600	-3.63410	0.18750
H	-6.11070	-2.15080	0.66910
H	-5.59510	-2.35920	-1.02020

11a +7 → dicyclohexyl-borane (TS$_2$)

Energy= -496.08393 a. u. (SMe_2 not included)

36

C	-1.20470	-0.25410	-0.18080
C	-1.78000	1.16670	0.01940
H	-1.23800	-0.46910	-1.26590
C	-3.24680	1.28710	-0.42600
H	-1.70700	1.43140	1.08580
H	-1.16990	1.90550	-0.51870
C	-4.13600	0.24950	0.27440
H	-3.62140	2.30160	-0.23240
H	-3.30660	1.13560	-1.51470
C	-3.59240	-1.17250	0.07180
H	-4.16980	0.47300	1.35150
H	-5.16910	0.32110	-0.09120
C	-2.12380	-1.28510	0.51200
H	-4.21120	-1.89730	0.61820
H	-3.67060	-1.43780	-0.99380
H	-2.06430	-1.14070	1.60200
H	-1.75470	-2.30220	0.32100

B	0.32730	-0.36870	0.32870
H	0.64940	-0.12160	1.46340
H	0.64760	-1.57100	0.23460
C	3.42540	0.84980	0.85480
C	4.06550	-0.31940	0.09770
H	4.18660	1.58630	1.13900
C	3.02500	-1.41090	-0.20730
H	4.88910	-0.75670	0.67480
H	4.50050	0.05040	-0.84160
C	1.74420	-0.83480	-0.81180
H	3.43380	-2.14810	-0.90880
H	2.79410	-1.95710	0.71350
C	1.40530	0.53580	-0.67940
H	1.32280	-1.38070	-1.65260
C	2.35190	1.52630	-0.00970
H	0.80910	0.95820	-1.48630
H	2.85190	2.10310	-0.80300
H	1.78170	2.24990	0.58410
H	2.97750	0.47990	1.78610

11a +7 → dicyclohexyl-borane (PS, dicyclohexyl-borane)

Energy= -496.12773 a. u. (SMe_2 not included)

36

B	-0.00000	-0.00040	0.76800
C	1.37030	0.19350	0.02100
C	2.36780	1.14730	0.71480
C	2.00090	-1.22990	-0.10530
H	1.20120	0.55020	-1.00940
C	3.73290	1.18540	0.01050
H	2.50540	0.82270	1.75700
H	1.94730	2.16080	0.76450
C	3.36630	-1.18620	-0.81260
H	2.13270	-1.66300	0.89790
H	1.32440	-1.90230	-0.64960
C	4.33760	-0.22050	-0.11820
H	4.42100	1.84620	0.55380
H	3.61130	1.61950	-0.99330
H	3.79660	-2.19570	-0.85250
H	3.21850	-0.86600	-1.85440
H	5.28750	-0.18030	-0.66680
H	4.57300	-0.60580	0.88530
C	-1.37040	-0.19380	0.02100
C	-2.36810	-1.14750	0.71460
C	-2.00060	1.22970	-0.10520
H	-1.20130	-0.55040	-1.00950
C	-3.73320	-1.18510	0.01030
H	-2.50560	-0.82300	1.75680
H	-1.94790	-2.16110	0.76420
C	-3.36600	1.18650	-0.81250
H	-2.13230	1.66280	0.89800
H	-1.32390	1.90200	-0.64940
C	-4.33750	0.22090	-0.11820
H	-4.42160	-1.84580	0.55350
H	-3.61180	-1.61910	-0.99350
H	-3.79600	2.19610	-0.85230

H	-3.21830	0.86640	-1.85430
H	-5.28750	0.18110	-0.66680
H	-4.57280	0.60620	0.88530
H	0.00000	-0.00070	1.97420

9 (dimer of dicyclohexyl-borane)

Energy= -992.25559 a. u.

72

H	0.00010	-0.00010	-0.96290
H	-0.00000	-0.00030	0.96280
B	-0.00010	0.93300	0.00000
C	1.32990	1.84950	0.13600
C	2.45030	1.46470	1.12680
C	1.92420	2.25200	-1.23890
H	0.91500	2.78630	0.54930
C	3.49500	2.58620	1.25780
H	2.96120	0.55110	0.80680
H	2.01760	1.24430	2.11280
C	2.98990	3.35270	-1.10830
H	2.37070	1.37220	-1.72080
H	1.12420	2.59440	-1.91010
C	4.08890	2.96480	-0.10770
H	4.29280	2.28070	1.94760
H	3.02010	3.47340	1.70300
H	3.42950	3.57110	-2.09070
H	2.50680	4.28130	-0.76910
H	4.80820	3.78660	0.00400
H	4.65430	2.10840	-0.50510
C	-1.33030	1.84920	-0.13590
C	-2.45070	1.46420	-1.12660
C	-1.92460	2.25170	1.23890
H	-0.91550	2.78610	-0.54930
C	-3.49540	2.58570	-1.25780
H	-2.96160	0.55070	-0.80640
H	-2.01810	1.24360	-2.11260
C	-2.99030	3.35250	1.10830
H	-2.37110	1.37200	1.72080
H	-1.12460	2.59420	1.91010
C	-4.08930	2.96450	0.10760
H	-4.29320	2.28010	-1.94760
H	-3.02040	3.47290	-1.70310
H	-3.43000	3.57090	2.09060
H	-2.50730	4.28100	0.76900
H	-4.80860	3.78630	-0.00410
H	-4.65480	2.10810	0.50510
B	0.00010	-0.93340	-0.00010
C	-1.32990	-1.84990	0.13580
C	-2.45000	-1.46510	1.12710
C	-1.92470	-2.25180	-1.23900
H	-0.91510	-2.78690	0.54870
C	-3.49490	-2.58630	1.25800
H	-2.96060	-0.55120	0.80750
H	-2.01690	-1.24520	2.11300
C	-2.99060	-3.35240	-1.10840
H	-2.37100	-1.37190	-1.72060

H	-1.12480	-2.59430	-1.91040
C	-4.08930	-2.96430	-0.10740
H	-4.29250	-2.28070	1.94810
H	-3.02010	-3.47380	1.70280
H	-3.43050	-3.57040	-2.09070
H	-2.50770	-4.28110	-0.76950
H	-4.80870	-3.78600	0.00420
H	-4.65450	-2.10770	-0.50450
C	1.33030	-1.84970	-0.13610
C	2.45050	-1.46460	-1.12700
C	1.92480	-2.25180	1.23880
H	0.91560	-2.78670	-0.54920
C	3.49560	-2.58570	-1.25790
H	2.96090	-0.55070	-0.80720
H	2.01760	-1.24460	-2.11300
C	2.99090	-3.35220	1.10830
H	2.37100	-1.37180	1.72060
H	1.12490	-2.59440	1.91010
C	4.08970	-2.96390	0.10760
H	4.29320	-2.27990	-1.94780
H	3.02100	-3.47320	-1.70300
H	3.43060	-3.57030	2.09070
H	2.50820	-4.28090	0.76920
H	4.80930	-3.78540	-0.00400
H	4.65480	-2.10720	0.50490

9 +7 → 8 (IS, dicyclohexyl-borane)

Energy= -496.12773 a. u. *(already displayed)*

9 +7 → 8 (TS_2)

Energy= -730.80832 a. u. (one monomer of dicyclohexyl-borane is not included)

52

B	-0.12330	-0.15700	-0.27640
C	-1.71120	0.26310	-0.30680
C	-2.67250	-0.68760	-1.05470
C	-2.29290	0.57420	1.09340
H	-1.74490	1.20860	-0.87140
C	-4.10770	-0.13810	-1.12890
H	-2.70820	-1.66790	-0.55020
H	-2.30470	-0.87370	-2.07400
C	-3.72490	1.13180	1.03010
H	-2.30380	-0.34370	1.70210
H	-1.64520	1.27970	1.62970
C	-4.66270	0.18690	0.26520
H	-4.76370	-0.85440	-1.64210
H	-4.10740	0.77750	-1.73890
H	-4.11040	1.31110	2.04310
H	-3.70880	2.10960	0.52550
H	-5.66650	0.62460	0.18370
H	-4.77670	-0.74790	0.83550
C	0.99600	0.97250	0.15420
C	2.39590	0.79090	-0.47360
C	0.50600	2.40540	-0.18390

H	1.12790	0.94630	1.25030
C	3.40590	1.86840	-0.04430
H	2.29590	0.81130	-1.57070
H	2.80560	-0.19230	-0.22520
C	1.50010	3.49500	0.25260
H	0.34320	2.48440	-1.27030
H	-0.46320	2.60550	0.28480
C	2.89080	3.27890	-0.35650
H	4.37330	1.69640	-0.53590
H	3.58730	1.78160	1.03790
H	1.11400	4.48610	-0.02210
H	1.58220	3.48710	1.35010
H	3.59540	4.03710	0.01020
H	2.83180	3.40850	-1.44780
H	0.17150	-0.46170	-1.46440
C	0.03780	-1.68470	0.62280
C	0.20150	-2.01290	-0.74250
C	1.19200	-1.93500	1.59360
C	1.46770	-2.64080	-1.30420
C	2.04820	-3.12980	1.13880
H	1.83180	-1.05110	1.69800
H	0.77720	-2.12620	2.58970
C	2.59180	-2.90040	-0.27710
H	1.84430	-2.08480	-2.16900
H	1.12780	-3.60830	-1.70180
H	2.88070	-3.28970	1.83500
H	1.43700	-4.04370	1.15970
H	3.17430	-3.76830	-0.60950
H	3.29020	-2.05730	-0.25680
H	-0.95080	-1.86650	1.03590
H	-0.69120	-2.25940	-1.30730

9 +7 → 8 (PS, 8)

Energy= -730.87256 a. u. (one monomer of dicyclohexyl-borane is not included)

52

C	1.56470	0.30370	-0.00170
C	2.25960	-0.25660	1.26940
C	3.77560	0.00210	1.26450
C	4.44040	-0.55480	-0.00280
C	3.77140	-0.00570	-1.27130
C	2.25600	-0.26600	-1.27030
C	-1.04500	1.20050	0.00430
C	-0.90770	2.07890	-1.26910
C	-1.89160	3.26070	-1.26730
C	-1.74080	4.12000	-0.00360
C	-1.87970	3.27070	1.26770
C	-0.89550	2.08900	1.26940
C	-0.51670	-1.50590	-0.00050
C	-1.35910	-1.82070	-1.26750
C	-1.89330	-3.26240	-1.26590
C	-2.70230	-3.56400	0.00400
C	-1.88540	-3.26560	1.26970
C	-1.35060	-1.82410	1.27150
B	0.00140	-0.00030	0.00020
H	1.74300	1.39140	-0.00580

H	1.81560	0.18570	2.17180
H	2.08310	-1.33980	1.33740
H	3.95660	1.08550	1.32190
H	4.23370	-0.43870	2.15990
H	5.51270	-0.32000	-0.00510
H	4.36120	-1.65250	0.00090
H	4.22790	-0.45060	-2.16550
H	3.95100	1.07770	-1.33450
H	2.08020	-1.34990	-1.33030
H	1.80930	0.17000	-2.17440
H	-2.07580	0.81000	0.01100
H	0.11820	2.46980	-1.33710
H	-1.06640	1.47080	-2.17020
H	-1.74250	3.87490	-2.16540
H	-2.91970	2.87290	-1.32030
H	-0.75110	4.60150	-0.01040
H	-2.48100	4.93070	-0.00330
H	-2.90730	2.88330	1.33310
H	-1.72280	3.89200	2.15950
H	-1.04620	1.48770	2.17650
H	0.13090	2.47970	1.32490
H	0.33410	-2.20660	-0.00450
H	-2.20910	-1.12490	-1.32500
H	-0.76040	-1.65230	-2.17320
H	-2.50860	-3.43540	-2.15890
H	-1.04630	-3.96150	-1.32940
H	-3.03640	-4.60970	0.00370
H	-3.61240	-2.94530	0.00740
H	-1.03830	-3.96520	1.32620
H	-2.49530	-3.44060	2.16600
H	-0.74570	-1.65840	2.17360
H	-2.20000	-1.12860	1.33650

13a + 1 (TS a)

Energy(6-31G*)= -493.49442 a. u.

32

C	1.90300	0.87900	0.96100
C	2.61600	1.39300	-0.29900
H	2.64300	0.78900	1.77000
C	3.73700	0.43300	-0.72600
H	3.02300	2.39800	-0.12700
H	1.88600	1.48000	-1.11400
C	3.29500	-1.00900	-0.65600
H	4.07100	0.67300	-1.74500
H	4.62300	0.57900	-0.08600
C	2.18800	-1.41000	-0.02000
H	3.92200	-1.74500	-1.15800
C	1.23700	-0.49700	0.71500
H	1.92300	-2.46800	-0.03900
H	1.03300	-0.95500	1.70000
B	-0.19300	-0.39500	-0.06500
H	-0.55800	-1.56900	-0.30100
H	1.16000	1.61300	1.30000
H	-0.31200	0.09400	-1.15700
C	-3.53300	-0.33700	-0.87700
C	-3.02900	1.08300	-0.90000

H	-4.37100	-0.58900	-1.52500
C	-2.25900	1.46300	0.38000
H	-3.86500	1.77600	-1.05100
H	-2.38000	1.20000	-1.77900
C	-1.39000	0.34000	0.93500
H	-1.65300	2.35600	0.19300
H	-2.98600	1.73600	1.16000
C	-1.81900	-1.00000	0.74600
H	-0.93600	0.56100	1.89900
C	-2.96300	-1.29800	-0.13600
H	-1.54900	-1.75300	1.48100
H	-3.31800	-2.32500	-0.17600

13a + 1 (TS b)

Energy(6-31G*)= -493.49379 a. u.

32

C	1.93600	0.93100	0.89800
C	2.67800	1.34700	-0.38000
H	2.66500	0.86500	1.72000
C	3.77100	0.32800	-0.73800
H	3.11600	2.34600	-0.26200
H	1.95900	1.41100	-1.20700
C	3.28300	-1.09200	-0.58600
H	4.12100	0.49500	-1.76700
H	4.65600	0.48500	-0.10000
C	2.15800	-1.42000	0.06000
H	3.89300	-1.87700	-1.03300
C	1.22600	-0.43400	0.72600
H	1.86300	-2.46900	0.10500
H	1.00200	-0.82700	1.73400
B	-0.17900	-0.34500	-0.08700
H	-0.57900	-1.49500	-0.33300
H	1.21600	1.70900	1.18300
H	-0.27300	0.19700	-1.15100
C	-3.37000	-0.29700	-1.09700
C	-3.19300	1.14200	-0.67400
H	-2.74000	-0.49600	-1.97600
C	-2.27400	1.48800	0.23500
C	-1.41900	0.48300	0.91400
C	-1.76200	-0.87600	0.80400
H	-0.88700	0.80900	1.80400
C	-3.00000	-1.27900	0.03200
H	-1.43100	-1.56500	1.57800
H	-3.80800	-1.29200	0.78000
H	-2.12200	2.53000	0.50700
H	-3.79700	1.90200	-1.16500
H	-4.40200	-0.48700	-1.41400
H	-2.91200	-2.30100	-0.35300

13a + 1 (TS c)

Energy(6-31G*)= -493.49122 a. u.

32

C	1.97700	0.70400	1.17600
C	2.40800	1.51800	-0.05600
H	2.86800	0.50300	1.79000
C	3.11100	0.66000	-1.07700
H	3.06600	2.34400	0.25200
H	1.53200	1.99600	-0.52200
C	3.09200	-0.67700	-1.04700
C	2.35500	-1.47900	-0.00100
H	3.63700	-1.23800	-1.80600
C	1.31700	-0.63700	0.78100
H	1.86600	-2.33900	-0.48000
H	1.09000	-1.18100	1.71300
B	-0.07800	-0.44900	-0.03400
H	-0.44900	-1.59100	-0.37800
H	1.30100	1.30500	1.79800
H	-0.17200	0.13700	-1.08000
C	-3.40100	-0.28000	-0.91300
C	-2.87800	1.13200	-0.83200
H	-4.22500	-0.47700	-1.59700
C	-2.13200	1.41700	0.48600
H	-3.70200	1.84600	-0.95500
H	-2.20900	1.29600	-1.68700
C	-1.29700	0.24600	0.98900
H	-1.50800	2.30900	0.36900
H	-2.87100	1.65500	1.26600
C	-1.73700	-1.06900	0.70400
H	-0.85300	0.39900	1.97100
C	-2.86100	-1.29400	-0.22200
H	-1.48600	-1.87400	1.38900
H	-3.22700	-2.31100	-0.33800
H	3.09600	-1.91400	0.69300
H	3.65400	1.17300	-1.87100

13a + 1 (TS d)

Energy(6-31G*)= -493.4906 a. u.

32

C	-2.01200	0.80800	-1.09400
C	-2.50100	1.48000	0.20100
H	-2.88200	0.63000	-1.74500
C	-3.17600	0.49700	1.12300
H	-3.19400	2.29800	-0.04200
H	-1.65700	1.95200	0.72600
C	-3.09800	-0.82800	0.96400
C	-2.31600	-1.49200	-0.14400
H	-3.62700	-1.48300	1.65700
C	-1.29900	-0.53600	-0.81700
H	-1.80200	-2.37900	0.25400
H	-1.04000	-0.97900	-1.79400
B	0.06500	-0.37900	0.04300
H	0.46400	-1.50200	0.38300
H	-1.35500	1.49700	-1.64100
H	0.13500	0.24400	1.06300
C	3.23700	-0.24400	1.12700

C	3.06500	1.16800	0.61900
H	2.58700	-0.39500	2.00100
C	2.16800	1.45700	-0.33100
C	1.33800	0.41000	-0.97500
C	1.68000	-0.93800	-0.77900
H	0.81900	0.68200	-1.88900
C	2.89800	-1.29100	0.04800
H	1.36900	-1.67100	-1.51800
H	3.72600	-1.34600	-0.67700
H	2.01800	2.48100	-0.66400
H	3.65200	1.95800	1.08200
H	4.26200	-0.41000	1.47900
H	2.80100	-2.28800	0.48900
H	-3.02900	-1.88100	-0.89300
H	-3.74900	0.90700	1.95400

13a + 13b (TS e)

Energy(6-31G*)= -520.14587 a. u.

36

C	-2.10000	1.04600	-0.87600
C	-2.54700	1.46100	0.53500
H	-2.96800	1.11200	-1.55000
C	-3.35000	0.37900	1.21200
H	-3.14000	2.38600	0.48400
H	-1.67100	1.70600	1.15600
C	-3.40800	-0.87800	0.76100
C	-2.66900	-1.36100	-0.46500
H	-4.02300	-1.60900	1.28600
C	-1.54100	-0.39500	-0.90500
H	-2.26000	-2.36300	-0.27100
H	-1.29800	-0.63600	-1.95400
B	-0.18900	-0.58000	-0.02500
H	0.06200	-1.79400	0.01800
H	-1.36000	1.76300	-1.25600
H	-0.11300	-0.26500	1.13000
C	2.98400	-1.01500	1.09700
C	3.30200	0.42000	0.65800
H	2.22200	-1.00500	1.88500
C	2.05300	1.15300	0.10800
C	1.17600	0.26200	-0.78000
C	1.39400	-1.12600	-0.88000
H	0.75700	0.74600	-1.66000
C	2.48300	-1.83500	-0.09900
H	1.06500	-1.63100	-1.78600
H	3.31100	-1.98200	-0.80900
H	1.45100	1.45200	0.99800
H	3.72700	0.99600	1.48800
H	3.87600	-1.49400	1.51800
H	2.15600	-2.83500	0.20700
H	-3.39800	-1.49700	-1.28300
H	-3.90100	0.65500	2.11100
H	4.07800	0.38300	-0.12100
B	2.30700	2.59800	-0.41400
H	1.60900	3.09300	-1.25200
H	3.15800	3.26900	0.09600

13a + 13b (TS f)

Energy(6-31G*)= -520.145814 a. u.

36

C	2.30700	-0.99900	-0.97900
C	2.79300	-1.41800	0.41800
H	3.18500	-0.85400	-1.62600
C	3.35500	-0.25200	1.19100
H	3.55500	-2.20600	0.32800
H	1.96800	-1.87200	0.99000
C	3.18200	1.02100	0.82400
C	2.39500	1.44200	-0.39400
H	3.63200	1.81600	1.42000
C	1.48800	0.31400	-0.94100
H	1.79600	2.33100	-0.14900
H	1.23100	0.56900	-1.98300
B	0.11000	0.13000	-0.11500
H	-0.42700	1.33100	-0.16100
H	1.72100	-1.81100	-1.42900
H	0.05900	-0.04700	1.06900
C	-3.59400	-0.04100	0.43400
C	-2.71200	-1.15200	1.01900
H	-4.23400	0.38800	1.21400
C	-1.85800	-1.80300	-0.07700
H	-3.34000	-1.91300	1.49800
H	-2.06300	-0.73900	1.80100
C	-1.05600	-0.78100	-0.88200
H	-1.17800	-2.54200	0.36000
H	-2.52100	-2.35500	-0.76100
C	-1.55600	0.57000	-0.99600
H	-0.64300	-1.16900	-1.81400
C	-2.77000	1.08700	-0.22600
H	-1.29900	1.13300	-1.89100
H	-3.38900	1.62900	-0.95200
H	3.10200	1.77400	-1.17300
H	3.92500	-0.48000	2.09200
H	-4.26800	-0.48400	-0.31300
B	-2.01700	2.10800	0.71900
H	-1.68900	3.16900	0.27300
H	-1.78700	1.84300	1.85900

13a + 13b (TS g)

Energy(6-31G*)= -520.145343 a. u.

36

C	2.34900	0.45500	1.27600
C	2.92700	1.36400	0.17800
H	3.18300	0.06600	1.88000
C	3.56300	0.56700	-0.93300
H	3.66700	2.05300	0.61300

H	2.13700	2.00600	-0.24000
C	3.38200	-0.74800	-1.08500
C	2.51200	-1.58400	-0.17600
H	3.88700	-1.26800	-1.89900
C	1.54900	-0.73000	0.68600
H	1.94600	-2.30700	-0.77900
H	1.22600	-1.36000	1.53300
B	0.22300	-0.26500	-0.12400
H	-0.27900	-1.28600	-0.64200
H	1.72500	1.04900	1.95700
H	0.26100	0.46600	-1.07800
C	-3.31400	0.29300	-0.92200
C	-2.36800	1.50500	-0.88500
H	-3.69100	0.10800	-1.93100
C	-1.68900	1.67000	0.47800
H	-2.93100	2.41100	-1.13900
H	-1.60200	1.37100	-1.65700
C	-0.98000	0.40000	0.93000
H	-0.97500	2.50000	0.43500
H	-2.44400	1.94700	1.23000
C	-1.45900	-0.86200	0.50200
H	-0.54700	0.44400	1.92800
C	-2.68200	-1.03900	-0.36400
H	-1.21100	-1.72600	1.11500
H	3.16700	-2.19400	0.47100
H	4.19700	1.10900	-1.63500
H	-4.21500	0.57000	-0.32800
H	-2.51000	-1.74400	-1.18000
B	-3.98800	-1.28300	0.45300
H	-4.07500	-0.88800	1.57900
H	-4.93900	-1.77200	-0.08000

S48(reactant)

Energy(6-31G*)= -753.862073366 a. u.

50

C	-4.76157	-1.50665	0.19363
C	-4.13019	-0.69728	-0.97219
C	-5.07815	0.49573	-1.28064
C	-4.96407	1.43789	-0.02788
C	-3.95936	0.72380	0.92007
C	-4.65278	-0.56386	1.44562
H	-5.80144	-1.78353	-0.02534
H	-4.76668	1.02029	-2.19249
H	-4.59596	2.42991	-0.31756
H	-5.63620	-0.33955	1.87977
H	-5.93424	1.59209	0.46263
H	-3.58357	1.37110	1.71743
H	-6.10848	0.15562	-1.44897
H	-3.90179	-1.30659	-1.85181
H	-4.21498	-2.44274	0.36224
B	-2.99451	0.08664	-0.17548
H	-4.05858	-1.03464	2.23858
C	-0.82390	-1.23717	-0.09202
C	-1.44393	0.15440	-0.41827
C	-0.69185	1.26162	0.34811
C	0.82392	1.23711	0.09209
C	1.44396	-0.15451	0.41837

C	0.69189	-1.26170	-0.34804
H	-1.00926	-1.48284	0.96520
H	-1.28185	0.30936	-1.50061
H	-1.09321	2.24805	0.07826
H	1.31618	2.01903	0.68434
H	1.09322	-2.24815	-0.07821
H	-0.87335	1.14170	1.42617
H	1.00931	1.48273	-0.96513
H	1.28181	-0.30942	1.50071
H	0.87340	-1.14176	-1.42609
H	-1.31614	-2.01910	-0.68427
B	2.99452	-0.08662	0.17557
C	5.07830	-0.49567	1.28051
C	4.13025	0.69730	0.97219
C	4.76147	1.50673	-0.19369
C	4.65280	0.56389	-1.44563
C	3.95929	-0.72372	-0.92010
C	4.96393	-1.43792	0.02784
H	6.10866	-0.15551	1.44853
H	4.21467	2.44271	-0.36230
H	4.05875	1.03465	-2.23871
H	5.93404	-1.59238	-0.46272
H	3.90192	1.30660	1.85185
H	4.76709	-1.02017	2.19248
H	4.59561	-2.42984	0.31763
H	5.80128	1.78383	0.02526
H	5.63628	0.33952	-1.87963
H	3.58343	-1.37099	-1.71745

S48(product)

Energy(6-31G*)= -780.474330663a. u.

54

C	-5.17876	-1.46943	0.26550
C	-4.56897	-0.69874	-0.93695
C	-5.48647	0.52390	-1.22259
C	-5.28596	1.47746	0.01061
C	-4.26771	0.73947	0.92595
C	-4.98388	-0.51517	1.49797
H	-6.23606	-1.71199	0.09443
H	-5.19599	1.02500	-2.15430
H	-4.89544	2.45172	-0.30849
H	-5.93960	-0.25040	1.96964
H	-6.22826	1.67260	0.53929
H	-3.83609	1.38375	1.69705
H	-6.53430	0.21853	-1.34230
H	-4.39889	-1.32661	-1.81675
H	-4.65888	-2.42231	0.42452
B	-3.37478	0.06091	-0.20573
H	-4.37365	-0.99692	2.27194
C	-1.22855	-1.26076	-0.01173
C	-1.83529	0.08473	-0.51485
C	-1.04712	1.27006	0.07977
C	0.46013	1.17502	-0.20350
C	1.06560	-0.15897	0.30137
C	0.28143	-1.34802	-0.29049

H	-1.39381	-1.35597	1.07251
H	-1.69572	0.08839	-1.61015
H	-1.43778	2.21862	-0.31341
H	0.97993	2.02689	0.25379
H	0.67050	-2.29618	0.10543
H	-1.20748	1.30110	1.16745
H	0.62511	1.25908	-1.28950
H	0.91586	-0.17483	1.39676
H	0.43885	-1.38306	-1.37896
H	-1.74264	-2.10906	-0.48200
B	2.64392	-0.20079	0.07721
C	4.69933	0.09256	1.63560
C	3.67467	0.83583	0.73509
C	4.46838	1.60308	-0.35700
C	5.34386	0.62742	-1.20088
C	5.17802	-0.83352	-0.70331
C	5.58617	-0.87340	0.79327
H	5.32949	0.83400	2.14472
H	3.77853	2.16174	-1.00207
H	5.08354	0.69487	-2.26470
H	6.64308	-0.58860	0.88414
H	3.12880	1.56316	1.35132
H	4.17516	-0.45450	2.42973
H	5.51459	-1.89898	1.17650
H	5.10463	2.35539	0.12804
H	6.40173	0.91346	-1.12858
H	5.85324	-1.48283	-1.27420
B	3.66235	-1.31605	-0.88121
H	3.04770	-1.47351	0.27010
H	2.86338	-0.33886	-1.24835
H	3.31362	-2.25309	-1.53801

S48(reactant) + BH_3 → **S48(product)** (TS2)

Energy(6-31G*)= -780.474330663a. u.

54

C	-4.91350	0.12040	1.42430
C	-3.54220	-0.54550	1.19500
C	-3.79820	-1.85650	0.38330
C	-4.08830	-1.36320	-1.07830
C	-4.24170	0.20090	-0.95820
C	-5.45260	0.42060	-0.00850
H	-5.59660	-0.52390	1.99370
H	-2.93800	-2.53130	0.40750
H	-3.28320	-1.63940	-1.76750
H	-6.27140	-0.25950	-0.27830
H	-5.01700	-1.77930	-1.48660
H	-4.36330	0.62920	-1.95740
H	-4.65230	-2.40800	0.79580
H	-2.97150	-0.71630	2.11220
H	-4.79810	1.04930	1.99540
B	-2.89600	0.25660	-0.02580
H	-5.85270	1.43430	-0.08010
C	-0.66740	-1.04370	-0.42500
C	-1.34080	0.35730	-0.36260
C	-0.57720	1.21760	0.68050
C	0.92950	1.30680	0.39150

C	1.59980	-0.08000	0.27620
C	0.83530	-0.95730	-0.73670
H	-0.79690	-1.55570	0.53960
H	-1.18950	0.82480	-1.34640
H	-0.99730	2.22930	0.71410
H	1.42090	1.90030	1.17260
H	1.26370	-1.96730	-0.76230
H	-0.72780	0.78570	1.68160
H	1.07680	1.85690	-0.55040
H	1.48990	-0.56830	1.27070
H	0.96760	-0.54210	-1.74730
H	-1.15290	-1.67340	-1.18110
B	3.16130	-0.05820	0.11730
C	5.16020	-0.01160	1.45780
C	4.26310	0.96420	0.64850
C	4.98970	1.26110	-0.69370
C	4.93170	-0.09050	-1.49300
C	4.18440	-1.07960	-0.55420
C	5.11280	-1.36320	0.65870
H	6.18380	0.37330	1.55690
H	4.48500	2.06340	-1.24600
H	4.39110	0.03850	-2.43890
H	6.11080	-1.68500	0.33280
H	3.99510	1.86800	1.20290
H	4.77450	-0.14670	2.47590
H	4.70920	-2.17110	1.28140
H	6.02140	1.59620	-0.52350
H	5.93240	-0.46230	-1.75020
H	3.84830	-1.98640	-1.06480
B	-3.46920	2.48890	-0.84230
H	-2.59410	2.55660	-1.64550
H	-4.55800	2.88930	-1.10850
H	-3.19790	2.33750	0.32290

Table S6 .B3LYP/6-31G(d) optimized geometries (cartesian coordinates in Å) for the structures shown in Table S4. Total energies in Hartrees at the B3LYP/6-311+G(d,p) level of theory.

S1

Energy= -522.796420191 a.u.

^{11}B chemical shift (ppm) = (B3) 80.0997 (B21) 80.0995

40

H	-0.00001	-0.00004	0.47415
H	0.00002	0.00028	-1.46907
B	0.61305	-0.65782	-0.49821
C	2.18874	-0.43990	-0.49474
C	2.69862	1.01416	-0.57537
C	2.82508	-1.16127	0.72211
H	2.55695	-0.96798	-1.39365
C	4.23397	1.08981	-0.59500
H	2.32213	1.58013	0.28993
H	2.28948	1.51325	-1.46344
C	4.35983	-1.08247	0.70743
H	2.45365	-0.69791	1.64993
H	2.50257	-2.20999	0.75123
C	4.85227	0.36916	0.61205
H	4.56029	2.13782	-0.62005
H	4.60520	0.62533	-1.52098
H	4.77065	-1.56583	1.60372
H	4.73910	-1.65045	-0.15526
H	5.94807	0.39626	0.55281
H	4.57642	0.90552	1.53254
B	-0.61305	0.65805	-0.49800
C	-2.18874	0.44010	-0.49464
C	-2.69859	-1.01394	-0.57581
C	-2.82511	1.16101	0.72247
H	-2.55694	0.96850	-1.39335
C	-4.23394	-1.08961	-0.59548
H	-2.32211	-1.58022	0.28930
H	-2.28943	-1.51270	-1.46405
C	-4.35986	1.08218	0.70774
H	-2.45368	0.69732	1.65012
H	-2.50262	2.20973	0.75197
C	-4.85227	-0.36941	0.61182
H	-4.56023	-2.13762	-0.62092
H	-4.60517	-0.62479	-1.52130
H	-4.77070	1.56521	1.60421
H	-4.73913	1.65048	-0.15474
H	-5.94808	-0.39651	0.55254
H	-4.57644	-0.90611	1.53212
H	-0.17987	1.77235	-0.49584
H	0.17987	-1.77210	-0.49642

S2

Energy= -522.794096623 a.u.

^{11}B chemical shift (ppm) = (B3) 100.5552 (B4) 59.4720

40

H	0.14894	1.83695	0.96129
H	-0.14895	1.83688	-0.96141
B	0.00000	2.68917	-0.00009
B	-0.00000	0.88583	-0.00003
C	1.36847	0.07648	-0.18554
C	2.52027	0.79139	-0.92500
C	1.87629	-0.44448	1.18737
H	1.11201	-0.82005	-0.77712
C	3.75759	-0.10788	-1.07635
H	2.80418	1.69792	-0.37225
H	2.18301	1.13166	-1.91344
C	3.11572	-1.34241	1.04424
H	2.13107	0.41668	1.82487
H	1.08124	-0.99210	1.71041
C	4.24408	-0.63159	0.28307
H	4.56313	0.44088	-1.58167
H	3.50806	-0.96168	-1.72433
H	3.46607	-1.65998	2.03523
H	2.83442	-2.25835	0.50354
H	5.09759	-1.30861	0.14871
H	4.60805	0.21377	0.88610
C	-1.36847	0.07650	0.18554
C	-2.52029	0.79150	0.92490
C	-1.87626	-0.44463	-1.18731
H	-1.11202	-0.81996	0.77724
C	-3.75762	-0.10775	1.07633
H	-2.80419	1.69796	0.37204
H	-2.18305	1.13189	1.91330
C	-3.11570	-1.34254	-1.04410
H	-2.13102	0.41644	-1.82492
H	-1.08120	-0.99232	-1.71026
C	-4.24407	-0.63163	-0.28304
H	-4.56317	0.44108	1.58156
H	-3.50811	-0.96147	1.72441
H	-3.46603	-1.66023	-2.03505
H	-2.83441	-2.25842	-0.50328
H	-5.09759	-1.30862	-0.14862
H	-4.60803	0.21366	-0.88619
H	1.02267	3.27660	-0.16601
H	-1.02266	3.27662	0.16578

S3

Energy= -757.529818878 a.u.

^{11}B chemical shift (ppm) = (B3) 66.9773 (B38) 86.4449

56

H	-0.21578	-0.83765	0.62038

H	-0.19017	-0.59036	-1.29608
B	-0.82817	-0.01517	-0.24946
C	-2.41537	-0.25965	-0.31163
C	-2.96774	-1.52521	-1.00007
C	-3.03009	-0.13482	1.10895
H	-2.79935	0.60289	-0.88715
C	-4.50442	-1.52120	-1.05114
H	-2.63547	-2.41981	-0.45662
H	-2.55854	-1.61686	-2.01525
C	-4.56739	-0.14774	1.08124
H	-2.67497	-0.97595	1.72479
H	-2.67897	0.78019	1.60428
C	-5.11147	-1.38546	0.35297
H	-4.86876	-2.43784	-1.53341
H	-4.84584	-0.68266	-1.67682
H	-4.96329	-0.10302	2.10458
H	-4.92232	0.75877	0.56869
H	-6.20681	-1.34092	0.29444
H	-4.86669	-2.28460	0.93821
C	-0.34785	1.50579	-0.02347
C	0.58015	1.75502	1.18941
C	0.23950	2.15746	-1.30173
H	-1.27565	2.06275	0.19262
C	0.89341	3.24795	1.38163
H	1.52335	1.20731	1.06042
H	0.11796	1.35356	2.10218
C	0.55297	3.64858	-1.09967
H	1.16057	1.63413	-1.59133
H	-0.46002	2.03681	-2.14100
C	1.47801	3.87249	0.10587
H	1.58511	3.38319	2.22361
H	-0.03229	3.77865	1.65015
H	1.00576	4.06496	-2.00928
H	-0.38731	4.19698	-0.93956
H	1.65621	4.94537	0.25474
H	2.45904	3.41946	-0.10202
B	0.23717	-1.48488	-0.43288
C	1.81951	-1.73300	-0.42834
C	2.77202	-0.64109	-0.95786
C	2.28068	-2.20967	0.97474
H	1.94965	-2.60005	-1.09894
C	4.23586	-1.11287	-0.96340
H	2.69601	0.26088	-0.33653
H	2.47651	-0.34202	-1.97263
C	3.74702	-2.67028	0.97865
H	2.16142	-1.38283	1.69199
H	1.63391	-3.02254	1.33143
C	4.68305	-1.58408	0.42845
H	4.89116	-0.30755	-1.32065
H	4.34382	-1.94308	-1.67752
H	4.05035	-2.95535	1.99481
H	3.83954	-3.57501	0.35957
H	5.71573	-1.95459	0.39187
H	4.68459	-0.72564	1.11712
H	-0.44053	-2.46054	-0.54692

S4

Energy= -288.051116741 a.u.

^{11}B chemical shift (ppm) = (B3) 93.0543 (B4) 71.2350

24

H	2.90765	0.28561	0.81553
H	2.52184	-0.43600	-0.95132
B	3.33172	0.42987	-0.40988
B	2.09041	-0.63856	0.30020
C	0.55548	-0.27739	0.48044
C	0.12243	1.13626	0.03759
C	-0.33233	-1.35060	-0.20341
H	0.35978	-0.35984	1.56564
C	-1.37633	1.38560	0.27094
H	0.34386	1.26469	-1.03284
H	0.71324	1.89847	0.56212
C	-1.83077	-1.09682	0.02681
H	-0.13360	-1.34670	-1.28692
H	-0.05948	-2.35092	0.15613
C	-2.24050	0.31620	-0.41304
H	-1.65280	2.38531	-0.08863
H	-1.57929	1.37586	1.35251
H	-2.42562	-1.84984	-0.50666
H	-2.05500	-1.22183	1.09669
H	-3.30284	0.48836	-0.19724
H	-2.12589	0.40242	-1.50411
H	4.44580	0.01464	-0.48302
H	3.04503	1.52114	-0.79161
H	2.49874	-1.70745	0.64615

9

Energy= -992.255151106 a.u.

^{11}B chemical shift (ppm) = (B3) 71.9680, (B38) 71.9501

Cartesian Coordinates displayed in Table S5.

8

Energy= -730.872258486 a.u.

^{11}B chemical shift (ppm) = 17.3968

Cartesian Coordinates displayed in Table S5.

S5

Energy= -1066.42912394 a.u.

^{11}B chemical shift (ppm) = (B3) 54.4667, (B38) 54.4710

71

B	0.00026	-1.35224	-0.00057
C	-1.05446	-2.13708	0.89798
C	-1.50967	-1.41786	2.18475
C	-2.29231	-2.49514	0.02583
H	-0.59536	-3.09719	1.19051
C	-2.55571	-2.22777	2.96796
H	-1.93866	-0.43966	1.92077
H	-0.64455	-1.20749	2.82762
C	-3.33594	-3.30744	0.80954
H	-2.76202	-1.56690	-0.33463
H	-1.98260	-3.05267	-0.86833
C	-3.76711	-2.58363	2.09344
H	-2.88005	-1.66897	3.85581
H	-2.09186	-3.15529	3.33610
H	-4.20901	-3.51003	0.17497
H	-2.90512	-4.28524	1.07174
H	-4.47776	-3.20108	2.65806
H	-4.30146	-1.66001	1.82415
C	1.05561	-2.13618	-0.89913
C	1.51108	-1.41679	-2.18565
C	2.29323	-2.49330	-0.02617
H	0.59722	-3.09661	-1.19180
C	2.55821	-2.22605	-2.96810
H	1.93920	-0.43825	-1.92155
H	0.64620	-1.20715	-2.82907
C	3.33801	-3.30486	-0.80911
H	2.76198	-1.56465	0.33449
H	1.98340	-3.05101	0.86783
C	3.76939	-2.58082	-2.09283
H	2.88263	-1.66714	-3.85585
H	2.09530	-3.15399	-3.33634
H	4.21086	-3.50663	-0.17396
H	2.90816	-4.28303	-1.07145
H	4.48088	-3.19772	-2.65698
H	4.30281	-1.65674	-1.82327
B	-0.00050	1.35169	-0.00011
C	-1.05539	2.13609	-0.89883
C	-1.51089	1.41692	-2.18547
C	-2.29304	2.49355	-0.02603
H	-0.59671	3.09640	-1.19134
C	-2.55764	2.22657	-2.96803
H	-1.93934	0.43850	-1.92149
H	-0.64598	1.20708	-2.82879
C	-3.33739	3.30553	-0.80910
H	-2.76217	1.56505	0.33450
H	-1.98314	3.05108	0.86805
C	-3.76882	2.58174	-2.09293
H	-2.88214	1.66780	-3.85584
H	-2.09436	3.15435	-3.33620
H	-4.21026	3.50760	-0.17408
H	-2.90713	4.28355	-1.07131
H	-4.47998	3.19896	-2.65715
H	-4.30266	1.65785	-1.82355
C	1.05397	2.13636	0.89884
C	1.50983	1.41711	2.18531
C	2.29151	2.49495	0.02637
H	0.59464	3.09628	1.19165
C	2.55578	2.22733	2.96836
H	1.93914	0.43911	1.92108

H	0.64499	1.20629	2.82841
C	3.33509	3.30753	0.80989
H	2.76143	1.56688	-0.33423
H	1.98145	3.05247	-0.86766
C	3.76685	2.58370	2.09360
H	2.88050	1.66853	3.85607
H	2.09169	3.15465	3.33669
H	4.20793	3.51048	0.17512
H	2.90399	4.28514	1.07230
H	4.47743	3.20133	2.65811
H	4.30143	1.66030	1.82404
O	-0.00011	-0.00027	-0.00034

S6

Energy= -650.080077274 a.u.

^{11}B chemical shift (ppm) = 51.4443

43

B	-0.08360	0.34070	0.03350
C	1.25140	-0.54230	0.04170
C	2.17680	-0.42610	-1.19460
C	2.06930	-0.33720	1.34750
H	0.91080	-1.59150	0.06850
C	3.36980	-1.39220	-1.10790
H	2.56400	0.59730	-1.28710
H	1.60750	-0.62190	-2.11360
C	3.27130	-1.29320	1.42700
H	2.43510	0.69890	1.40330
H	1.42470	-0.48030	2.22540
C	4.17000	-1.18190	0.18650
H	4.02260	-1.26730	-1.98210
H	2.99940	-2.42760	-1.14000
H	3.85270	-1.09290	2.33690
H	2.90120	-2.32550	1.51340
H	4.99320	-1.90550	0.24860
H	4.63410	-0.18400	0.16540
C	-1.48580	-0.36050	0.32310
C	-2.60740	0.58340	0.80310
C	-1.94830	-1.13250	-0.94530
H	-1.32240	-1.12430	1.10520
C	-3.93970	-0.15040	1.02200
H	-2.74750	1.37960	0.05890
H	-2.30250	1.08910	1.72890
C	-3.28070	-1.86710	-0.72490
H	-2.06890	-0.42010	-1.77620
H	-1.17840	-1.84970	-1.26140
C	-4.37920	-0.91070	-0.23790
H	-4.71970	0.56080	1.32540
H	-3.82990	-0.86420	1.85270
H	-3.59400	-2.36650	-1.65170
H	-3.13300	-2.65990	0.02370
H	-5.30800	-1.46450	-0.04740
H	-4.60540	-0.18690	-1.03550
O	-0.15350	1.68120	-0.20570
C	0.90660	2.58750	-0.51360
H	1.81130	2.32960	0.04780
H	1.14180	2.50850	-1.58230

C	0.45100	3.99860	-0.17380
H	1.23390	4.72350	-0.42420
H	-0.45330	4.25540	-0.73490
H	0.22630	4.08490	0.89440

S7

Energy= -569.286914295 a.u.

^{11}B chemical shift (ppm) = 73.5993

43

B	-0.08360	0.34070	0.03350
C	1.25140	-0.54230	0.04170
C	2.17680	-0.42610	-1.19460
C	2.06930	-0.33720	1.34750
H	0.91080	-1.59150	0.06850
C	3.36980	-1.39220	-1.10790
H	2.56400	0.59730	-1.28710
H	1.60750	-0.62190	-2.11360
C	3.27130	-1.29320	1.42700
H	2.43510	0.69890	1.40330
H	1.42470	-0.48030	2.22540
C	4.17000	-1.18190	0.18650
H	4.02260	-1.26730	-1.98210
H	2.99940	-2.42760	-1.14000
H	3.85270	-1.09290	2.33690
H	2.90120	-2.32550	1.51340
H	4.99320	-1.90550	0.24860
H	4.63410	-0.18400	0.16540
C	-1.48580	-0.36050	0.32310
C	-2.60740	0.58340	0.80310
C	-1.94830	-1.13250	-0.94530
H	-1.32240	-1.12430	1.10520
C	-3.93970	-0.15040	1.02200
H	-2.74750	1.37960	0.05890
H	-2.30250	1.08910	1.72890
C	-3.28070	-1.86710	-0.72490
H	-2.06890	-0.42010	-1.77620
H	-1.17840	-1.84970	-1.26140
C	-4.37920	-0.91070	-0.23790
H	-4.71970	0.56080	1.32540
H	-3.82990	-0.86420	1.85270
H	-3.59400	-2.36650	-1.65170
H	-3.13300	-2.65990	0.02370
H	-5.30800	-1.46450	-0.04740
H	-4.60540	-0.18690	-1.03550
O	-0.15350	1.68120	-0.20570
C	0.90660	2.58750	-0.51360
H	1.81130	2.32960	0.04780
H	1.14180	2.50850	-1.58230
C	0.45100	3.99860	-0.17380
H	1.23390	4.72350	-0.42420
H	-0.45330	4.25540	-0.73490
H	0.22630	4.08490	0.89440

S8

Energy= -488.492052349 a.u.

^{11}B chemical shift (ppm) = 85.6238

43

B	-0.08360	0.34070	0.03350
C	1.25140	-0.54230	0.04170
C	2.17680	-0.42610	-1.19460
C	2.06930	-0.33720	1.34750
H	0.91080	-1.59150	0.06850
C	3.36980	-1.39220	-1.10790
H	2.56400	0.59730	-1.28710
H	1.60750	-0.62190	-2.11360
C	3.27130	-1.29320	1.42700
H	2.43510	0.69890	1.40330
H	1.42470	-0.48030	2.22540
C	4.17000	-1.18190	0.18650
H	4.02260	-1.26730	-1.98210
H	2.99940	-2.42760	-1.14000
H	3.85270	-1.09290	2.33690
H	2.90120	-2.32550	1.51340
H	4.99320	-1.90550	0.24860
H	4.63410	-0.18400	0.16540
C	-1.48580	-0.36050	0.32310
C	-2.60740	0.58340	0.80310
C	-1.94830	-1.13250	-0.94530
H	-1.32240	-1.12430	1.10520
C	-3.93970	-0.15040	1.02200
H	-2.74750	1.37960	0.05890
H	-2.30250	1.08910	1.72890
C	-3.28070	-1.86710	-0.72490
H	-2.06890	-0.42010	-1.77620
H	-1.17840	-1.84970	-1.26140
C	-4.37920	-0.91070	-0.23790
H	-4.71970	0.56080	1.32540
H	-3.82990	-0.86420	1.85270
H	-3.59400	-2.36650	-1.65170
H	-3.13300	-2.65990	0.02370
H	-5.30800	-1.46450	-0.04740
H	-4.60540	-0.18690	-1.03550
O	-0.15350	1.68120	-0.20570
C	0.90660	2.58750	-0.51360
H	1.81130	2.32960	0.04780
H	1.14180	2.50850	-1.58230
C	0.45100	3.99860	-0.17380
H	1.23390	4.72350	-0.42420
H	-0.45330	4.25540	-0.73490
H	0.22630	4.08490	0.89440

S9

Energy= -729.862240714 a.u.

^{11}B chemical shift (ppm) = 72.6744

51

B	-0.17010	0.07390	-0.64740
C	-1.58590	-0.33220	0.02490
C	-2.70370	0.72120	-0.15520
C	-2.06890	-1.68240	-0.56770
H	-1.47510	-0.50090	1.11230
C	-4.05840	0.27480	0.41880
H	-2.82340	0.92990	-1.23000
H	-2.41670	1.67520	0.30950
C	-3.42370	-2.13800	0.00000
H	-2.15400	-1.58430	-1.66130
H	-1.32130	-2.46550	-0.38980
C	-4.50670	-1.06550	-0.17980
H	-4.82140	1.04530	0.24100
H	-3.97120	0.16950	1.51120
H	-3.73670	-3.07740	-0.47580
H	-3.30850	-2.35550	1.07290
H	-5.45120	-1.39270	0.27510
H	-4.70690	-0.93110	-1.25360
C	1.09780	-0.91180	-0.46400
C	2.41250	-0.40620	-1.09730
C	1.38490	-1.46820	0.94990
H	0.79070	-1.78330	-1.07450
C	3.52380	-1.46960	-1.09730
H	2.76800	0.47790	-0.54510
H	2.22300	-0.06890	-2.12530
C	2.48240	-2.54600	0.94920
H	1.71500	-0.65090	1.61170
H	0.46800	-1.87430	1.39710
C	3.77870	-2.02430	0.31210
H	4.45220	-1.05550	-1.51470
H	3.22740	-2.29720	-1.75940
H	2.67670	-2.89800	1.97180
H	2.12710	-3.41880	0.38090
H	4.53700	-2.81810	0.28030
H	4.19310	-1.22450	0.94550
H	-0.27870	0.46940	-1.78960
O	0.29410	1.66200	0.07650
C	0.07900	2.85170	-0.73390
H	-0.78810	2.64700	-1.36390
H	-0.17220	3.67400	-0.05590
C	1.30150	3.17920	-1.57290
H	1.09130	4.05860	-2.19330
H	2.17280	3.39790	-0.94760
H	1.54820	2.34270	-2.23140
C	0.25650	1.86900	1.50910
H	0.14280	0.87400	1.93660
H	-0.64140	2.44910	1.75530
C	1.51760	2.54470	2.02570
H	1.64380	3.55030	1.61050
H	1.45890	2.63970	3.11620
H	2.40370	1.95240	1.77800

51

S10

Energy= -974.198257833 a.u.

^{11}B chemical shift (ppm) = 83.2267

51

B	-0.17010	0.07390	-0.64740
C	-1.58590	-0.33220	0.02490
C	-2.70370	0.72120	-0.15520
C	-2.06890	-1.68240	-0.56770
H	-1.47510	-0.50090	1.11230
C	-4.05840	0.27480	0.41880
H	-2.82340	0.92990	-1.23000
H	-2.41670	1.67520	0.30950
C	-3.42370	-2.13800	0.00000
H	-2.15400	-1.58430	-1.66130
H	-1.32130	-2.46550	-0.38980
C	-4.50670	-1.06550	-0.17980
H	-4.82140	1.04530	0.24100
H	-3.97120	0.16950	1.51120
H	-3.73670	-3.07740	-0.47580
H	-3.30850	-2.35550	1.07290
H	-5.45120	-1.39270	0.27510
H	-4.70690	-0.93110	-1.25360
C	1.09780	-0.91180	-0.46400
C	2.41250	-0.40620	-1.09730
C	1.38490	-1.46820	0.94990
H	0.79070	-1.78330	-1.07450
C	3.52380	-1.46960	-1.09730
H	2.76800	0.47790	-0.54510
H	2.22300	-0.06890	-2.12530
C	2.48240	-2.54600	0.94920
H	1.71500	-0.65090	1.61170
H	0.46800	-1.87430	1.39710
C	3.77870	-2.02430	0.31210
H	4.45220	-1.05550	-1.51470
H	3.22740	-2.29720	-1.75940
H	2.67670	-2.89800	1.97180
H	2.12710	-3.41880	0.38090
H	4.53700	-2.81810	0.28030
H	4.19310	-1.22450	0.94550
H	-0.27870	0.46940	-1.78960
O	0.29410	1.66200	0.07650
C	0.07900	2.85170	-0.73390
H	-0.78810	2.64700	-1.36390
H	-0.17220	3.67400	-0.05590
C	1.30150	3.17920	-1.57290
H	1.09130	4.05860	-2.19330
H	2.17280	3.39790	-0.94760
H	1.54820	2.34270	-2.23140
C	0.25650	1.86900	1.50910
H	0.14280	0.87400	1.93660
H	-0.64140	2.44910	1.75530
C	1.51760	2.54470	2.02570
H	1.64380	3.55030	1.61050
H	1.45890	2.63970	3.11620
H	2.40370	1.95240	1.77800

11a.SMe_2

Energy= -739.462449432 a.u.

^{11}B chemical shift (ppm) = 103.0477

Cartesian Coordinates displayed in Table S5.

ref

Energy= -558.417226032 a.u.

^{11}B chemical shift (ppm) = 101.5949

19

B	-1.33131	0.15813	-0.08739
F	-1.68785	-1.08633	-0.52811
F	-1.51659	1.17404	-0.98194
F	-1.74548	0.44348	1.19062
O	0.33156	0.05208	0.03420
C	0.84315	-1.16551	0.68356
C	1.02466	1.29855	0.36833
C	1.27834	-2.17942	-0.35386
H	1.66706	-0.85818	1.32871
H	0.03458	-1.54187	1.30960
C	2.36922	1.38492	-0.32647
H	0.35322	2.08284	0.02780
H	1.10761	1.35343	1.45671
H	1.63410	-3.08053	0.15451
H	2.08888	-1.79206	-0.97474
H	2.82822	2.34886	-0.08994
H	3.05810	0.60242	0.00039
H	0.44025	-2.45312	-0.99475
H	2.24924	1.31906	-1.40946

nmr1

Energy= -557.205242259 a.u.

^{11}B chemical shift (ppm) = 102.1464

17

B	-1.47019	0.00006	-0.02080
F	-1.87217	-1.15652	-0.62505
F	-1.87226	1.15928	-0.61990
F	-1.58867	-0.00299	1.34923
O	0.17551	0.00070	-0.28368
C	0.93136	-1.17597	0.14496
C	0.93166	1.17436	0.15126
C	2.39007	-0.77739	-0.08839
H	0.70166	-1.35302	1.19843

H	0.57493	-2.00797	-0.45603
C	2.38776	0.77900	-0.09727
H	0.56990	2.01181	-0.43891
H	0.70973	1.34070	1.20826
H	2.74700	-1.16358	-1.04361
H	3.03010	-1.18536	0.69483
H	2.72837	1.15499	-1.06253
H	3.03805	1.19857	0.67122

nmr2

Energy= -504.721529593 a.u.

^{11}B chemical shift (ppm) = 121.6515

13

S	-0.02428	-0.00015	-0.53472
C	0.82834	-1.39537	0.26915
C	0.82182	1.39905	0.26908
H	1.87901	1.40923	-0.00745
H	0.33794	2.30949	-0.09064
H	0.69958	1.32792	1.35292
H	1.88553	-1.40090	-0.00749
H	0.70587	-1.32463	1.35301
H	0.34853	-2.30808	-0.09032
B	-1.82613	-0.00393	0.31174
H	-2.32104	1.00952	-0.12194
H	-1.60038	-0.00323	1.50086
H	-2.31690	-1.01945	-0.12162

nmr3

Energy= -53.3044086633 a.u.

^{11}B chemical shift (ppm) = (B1) 84.4795 (B5) 84.4721

8

B	-0.87728	0.00004	-0.00004
H	-1.44825	1.03834	0.00037
H	-0.00001	-0.00187	0.97280
H	-1.45002	-1.03732	-0.00021
B	0.87728	0.00004	-0.00002
H	1.44822	1.03836	0.00034
H	1.45006	-1.03730	-0.00025
H	-0.00004	-0.00063	-0.97279

nmr4

Energy= -252.585642094 a.u.

^{11}B chemical shift (ppm) = 83.0906

7

B	-0.00008	-0.00021	0.00016
O	-1.36972	0.00233	-0.00002
H	-1.74328	-0.88387	-0.00001
O	0.68290	-1.18691	-0.00008
H	1.63711	-1.06781	0.00052
O	0.68693	1.18479	-0.00014
H	0.10568	1.95101	0.00056

nmr5

Energy= -262.616999641 a.u.

^{11}B chemical shift (ppm) = 13.0776

22

B	-0.12109	-0.09438	0.29592
C	0.15130	1.40135	-0.13131
H	-0.16310	1.47734	-1.18607
H	-0.57691	2.04446	0.38455
C	1.56392	1.98273	0.02930
H	1.61884	3.01636	-0.32545
H	1.87946	1.98198	1.07703
H	2.30613	1.40675	-0.53066
C	1.05276	-1.09336	0.65398
H	0.70486	-1.89512	1.31662
H	1.87966	-0.58966	1.16720
C	1.60653	-1.74231	-0.64233
H	2.00837	-0.99350	-1.33125
H	2.41288	-2.44571	-0.41682
H	0.82905	-2.29657	-1.17697
C	-1.59889	-0.65356	0.34383
H	-1.60421	-1.69362	-0.01263
H	-1.80753	-0.75860	1.42392
C	-2.73793	0.13632	-0.31798
H	-2.58784	0.22366	-1.39860
H	-3.70811	-0.34526	-0.16335
H	-2.81226	1.15240	0.07992

nmr6

Energy= -720.004814513 a.u.

^{11}B chemical shift (ppm) = 38.5605

34

B	-0.00018	-0.00124	-0.00100
C	0.38390	1.51920	0.00001
C	1.52713	1.98983	0.67803
C	-0.39842	2.47616	-0.67847
C	1.86178	3.34145	0.69384
H	2.15390	1.28526	1.21359
C	-0.05041	3.82442	-0.69520
H	-1.28510	2.15371	-1.21310
C	1.07725	4.26162	-0.00092
H	2.73654	3.67727	1.24020

H	-0.66070	4.53531	-1.24171
H	1.34315	5.31321	-0.00133
C	1.12473	-1.09378	-0.00138
C	0.95708	-2.32293	0.66883
C	2.34896	-0.88987	-0.67054
C	1.96112	-3.28766	0.68609
H	0.02997	-2.51708	1.19686
C	3.34380	-1.86410	-0.68563
H	2.51537	0.04220	-1.19925
C	3.15490	-3.06327	0.00089
H	1.81183	-4.21631	1.22628
H	4.26819	-1.68712	-1.22477
H	3.93353	-3.81843	0.00209
C	-1.50922	-0.42768	-0.00105
C	-2.48675	0.32838	0.67785
C	-1.94873	-1.58373	-0.67819
C	-3.82495	-0.05634	0.69578
H	-2.18825	1.22373	1.21196
C	-3.29064	-1.95548	-0.69243
H	-1.22762	-2.19101	-1.21421
C	-4.23152	-1.19604	0.00251
H	-4.55190	0.53455	1.24251
H	-3.60270	-2.83950	-1.23800
H	-5.27543	-1.49062	0.00373

nmr7

Energy= -643.114937188 a.u.

^{11}B chemical shift (ppm) = 72.6651

32

B	-0.12881	-0.00031	-0.00008
C	-1.68091	-0.00036	-0.00008
C	-2.40202	1.19331	0.16725
C	-2.40253	-1.19367	-0.16731
C	-3.79474	1.19606	0.16866
H	-1.86075	2.12421	0.29737
C	-3.79527	-1.19581	-0.16859
H	-1.86172	-2.12483	-0.29753
C	-4.49307	0.00027	0.00008
H	-4.33657	2.12642	0.30015
H	-4.33749	-2.12593	-0.30003
H	-5.57783	0.00048	0.00014
O	0.63359	-1.12697	-0.19474
O	0.63321	1.12662	0.19462
C	2.02212	-0.78502	0.09097
C	2.02199	0.78503	-0.09105
C	2.29831	-1.23133	1.53103
H	3.34102	-1.06299	1.81114
H	1.65773	-0.70545	2.24197
H	2.08842	-2.29970	1.61481
C	2.91806	-1.55066	-0.87756
H	3.96673	-1.27245	-0.73889
H	2.82512	-2.62267	-0.68973
H	2.64156	-1.36517	-1.91514
C	2.29808	1.23170	-1.53100
H	3.34076	1.06339	-1.81126

H	1.65742	0.70604	-2.24206
H	2.08828	2.30010	-1.61450
C	2.91754	1.55081	0.87770
H	2.64128	1.36467	1.91522
H	3.96637	1.27332	0.73881
H	2.82390	2.62284	0.69032

nmr8

Energy= -525.134350987 a.u.

^{11}B chemical shift (ppm) = 67.0602

23

C	2.55657	1.28648	0.00020
C	3.61555	0.36920	0.00018
C	3.38576	-1.00919	0.00002
C	2.08623	-1.53262	-0.00013
C	1.05218	-0.61688	-0.00012
C	1.28055	0.75679	0.00004
H	2.72357	2.35617	0.00033
H	4.63418	0.73870	0.00029
H	4.22916	-1.68948	0.00000
H	1.89654	-2.59854	-0.00026
O	0.06790	1.41207	0.00002
O	-0.30841	-0.84382	-0.00026
B	-0.90709	0.41403	-0.00019
C	-2.44194	0.68139	-0.00046
H	-2.67920	1.31363	0.86680
H	-2.67905	1.31191	-0.86902
C	-3.33750	-0.56940	0.00065
H	-3.10034	-1.18570	0.87431
H	-3.10000	-1.18751	-0.87162
C	-4.83211	-0.23714	0.00003
H	-5.44253	-1.14466	0.00095
H	-5.10742	0.34980	0.88224
H	-5.10712	0.34782	-0.88359

nmr9

Energy= -638.271771499 a.u.

^{11}B chemical shift (ppm) = 70.5934

24

C	3.02868	1.42916	-0.00002
C	4.22405	0.69872	-0.00003
C	4.22405	-0.69872	-0.00001
C	3.02868	-1.42916	0.00002
C	1.85735	-0.69666	0.00003
C	1.85735	0.69666	0.00000
H	3.01720	2.51172	-0.00002
H	5.16794	1.23082	-0.00003
H	5.16794	-1.23082	-0.00002
H	3.01720	-2.51172	0.00002
O	0.55462	1.14536	0.00003

O	0.55462	-1.14536	-0.00001
B	-0.24039	-0.00000	0.00001
C	-1.77950	-0.00000	0.00000
C	-2.49960	-1.20706	-0.00001
C	-2.49959	1.20705	0.00001
C	-3.89143	-1.20831	-0.00002
H	-1.96074	-2.14825	0.00001
C	-3.89143	1.20831	0.00000
H	-1.96073	2.14825	-0.00000
C	-4.58870	0.00000	-0.00001
H	-4.43352	-2.14742	-0.00003
H	-4.43351	2.14742	0.00001
H	-5.67327	0.00000	-0.00002

nmr10

Energy= -408.390248579 a.u.

^{11}B chemical shift (ppm) = 72.5310

16

B	1.76992	-0.00010	0.00003
O	2.50519	-1.07701	-0.41289
H	1.97289	-1.78330	-0.78655
O	2.50498	1.07714	0.41291
H	1.97222	1.78334	0.78614
C	0.19373	-0.00021	0.00005
C	-0.53645	-1.18006	0.22231
C	-0.53614	1.17984	-0.22220
C	-1.92954	-1.18344	0.22972
H	-0.01210	-2.11157	0.41680
C	-1.92925	1.18358	-0.22978
H	-0.01156	2.11125	-0.41657
C	-2.62928	0.00016	-0.00009
H	-2.46886	-2.10569	0.41560
H	-2.46828	2.10599	-0.41569
H	-3.71359	0.00027	-0.00014

nmr11

Energy= -83.2500176761 a.u.

^{11}B chemical shift (ppm) = 122.5068

8

B	0.00000	0.00000	-0.93464
H	-1.01141	0.58394	-1.24440
H	0.00000	-1.16788	-1.24440
H	1.01141	0.58394	-1.24440
N	0.00000	0.00000	0.73025
H	0.00000	0.94885	1.09822
H	-0.82173	-0.47443	1.09822
H	0.82173	-0.47443	1.09822

nmr12

Energy= -1063.16748383 a.u.

^{11}B chemical shift (ppm) = 143.8923

38

B	-0.00146	0.00042	2.89550
H	-0.75021	-0.89359	3.21005
H	-0.40120	1.09539	3.21156
H	1.14668	-0.20175	3.21139
P	-0.00107	0.00063	0.94909
C	0.51200	-1.60825	0.22389
C	1.53625	-2.32170	0.86136
C	-0.08159	-2.14239	-0.92550
C	1.96735	-3.54117	0.34556
H	1.98414	-1.92755	1.76574
C	0.35050	-3.36582	-1.43599
H	-0.88696	-1.61126	-1.41845
C	1.37679	-4.06472	-0.80407
H	2.75882	-4.08616	0.84752
H	-0.11892	-3.77281	-2.32464
H	1.71009	-5.01714	-1.20089
C	-1.65060	0.36112	0.22298
C	-1.81508	1.13488	-0.93171
C	-2.78185	-0.15990	0.86574
C	-3.09047	1.37371	-1.44197
H	-0.95189	1.56012	-1.42956
C	-4.05339	0.07829	0.35050
H	-2.66574	-0.73942	1.77381
C	-4.21023	0.84375	-0.80437
H	-3.20696	1.97751	-2.33503
H	-4.92196	-0.32783	0.85657
H	-5.20158	1.03228	-1.20103
C	1.13672	1.24767	0.22266
C	1.25856	2.48547	0.86853
C	1.88301	1.00383	-0.93622
C	2.10116	3.46628	0.35158
H	0.70363	2.67401	1.77979
C	2.72835	1.98742	-1.44793
H	1.81452	0.04490	-1.43591
C	2.83604	3.22013	-0.80754
H	2.18954	4.41988	0.85978
H	3.30466	1.78693	-2.34424
H	3.49561	3.98332	-1.20528
